# Controlled Anchoring of (Phenylureido)sulfonamide-Based Receptor Moieties: An Impact of Binding Site Multiplication on Complexation Properties

**DOI:** 10.3390/molecules26185670

**Published:** 2021-09-18

**Authors:** Karolína Salvadori, Alena Krupková, Lucie Červenková Šťastná, Monika Müllerová, Václav Eigner, Tomáš Strašák, Petra Cuřínová

**Affiliations:** 1 Institute of Chemical Process Fundamentals of CAS, v.v.i., Rozvojová 135, 16502 Prague 6, Czech Republic; lottesalvadori@gmail.com (K.S.); krupkova@icpf.cas.cz (A.K.); stastna@icpf.cas.cz (L.Č.Š.); mullerovam@icpf.cas.cz (M.M.); strasak@icpf.cas.cz (T.S.); 2 Department of Solid State Chemistry, University of Chemistry and Technology Prague, Technická 5, 16828 Prague 6, Czech Republic; vaclav.eigner@vscht.cz

**Keywords:** host-guest chemistry, dendrimers, supramolecular chemistry

## Abstract

The repetition of urea-based binding units within the receptor structure does not only lead to monomer properties multiplication. As confirmed by spectroscopic studies, UV-Vis and ^1^H-NMR in classical or competitive titration mode, the attachment to a carrier allocates the active moieties to mutual positions predetermining the function of the whole receptor molecule. Bivalent receptors form self-aggregates. Dendritic receptors with low dihydrogen phosphate loadings offer a cooperative complexation mode associated with a positive dendritic effect. In higher dihydrogen phosphate concentrations, the dendritic branches act independently and the binding mode changes to 1:1 anion: complexation site. Despite the anchoring, the dendritic receptors retain the superior efficiency and selectivity of a monomer, paving the way to recyclable receptors, desirable for economic and ecological reasons.

## 1. Introduction

Shortly after synthesis of the first compounds with dendritic structure [1,2], features accompanying the defined multiplication of structural subunits were recognized. The fascination of the scientific community with observed unexpected generation-dependent properties of dendrimers led to a broad range of reports on so-called “dendritic effect” [3,4,5]. After a deeper examination of its origin, Tomalia published a study relating structure parameters to prediction of dendrimer properties [6,7]. Then, advantageously, the concept of formation of microenvironment with locally modified characteristics was employed in drug delivery [8,9], catalysis [10,11,12] or material [13,14] and supramolecular chemistry [15,16]. Although all these applications are based on an interaction of the dendritic molecule with a guest, the studies concerning dendritic effects in anion recognition are rather rare. In this context, Astruc et al. observed an enhancement in affinity of ferrocene-based dendritic molecules toward anions in comparison with monovalent receptors [17,18]. Dendritic ureas and thioureas prepared by Boas showed an increase in carboxylate binding affinity with increasing number of binding units [19]. Vögtle’s ureas in higher generation showed positive dendritic effect in ATP binding [20]. In our recent work we studied dihydrogen phosphate recognition in a series of carbosilane dendrimers with isophthalamide-based binding sites and we observed an enhanced, generation-dependent complexation ability at higher receptor concentrations [21]. The dendrimers prepared by Losada et al. with amidoferrocenyl units attached poly(propylene imine) dendrimers showed generation-dependent electrochemical responses to inorganic anions [22,23].

Here we report a systematic study of dendritic effects associated with anion recognition by receptor series with increasing number of the active units. We focused on the molecules carrying potent binding sites based on urea moieties conjugated to sulfonamide group. Such moiety has a superior affinity to anions with preferential 1:1 binding stoichiometry [24], even when two binding sites are present in close proximity within one molecule [25]. To induce a multiplication effect, the number of complexation sites within the receptor molecule was gradually increased, finally using their attachment to the periphery of carbosilane dendrimers. This study attempts to exploit the phenomenon of the multivalency in design of potent and possibly recyclable receptors.

## 2. Results

**Scheme 1:**
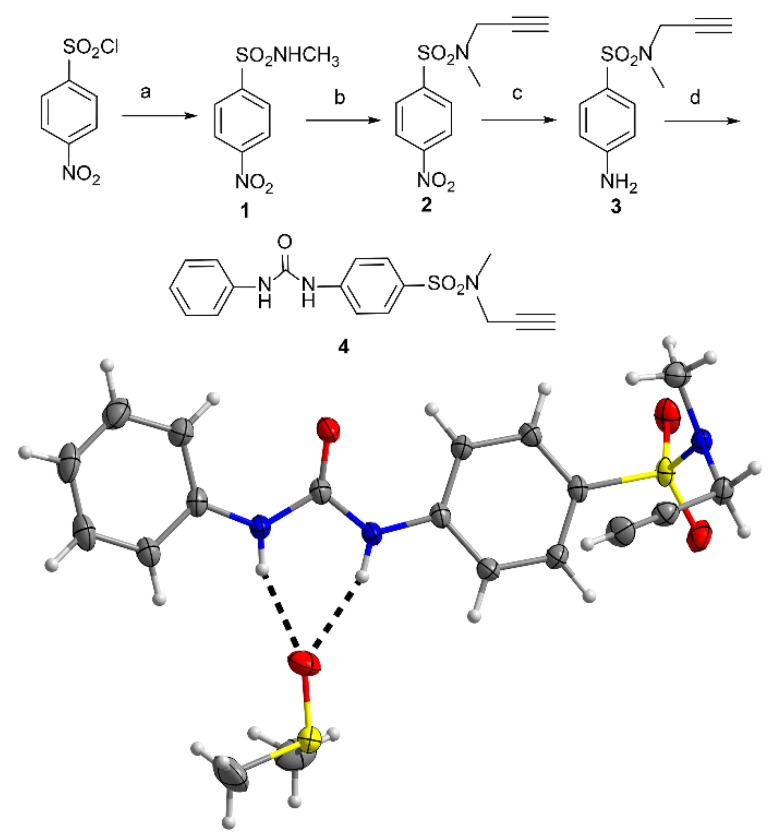
Synthesis of receptor moiety **4** and its crystal structure; (a) CH_3_NH_2_, pyridine, 93 %; (b) propargyl bromide, K_2_CO_3_, 33 %; (c) SnCl_2_ 2H_2_O, EtOH, quantitative, (d) PhNCO, 42 %.

The influence of binding sites accumulation on anion complexation was studied in a series of receptors differing in the number of receptor moieties. A receptor moiety **4** was synthesized, comprising following structural elements: (i) a urea-based complexation site, capable of donation of highly directional hydrogen bonds, (ii) a sulfonamide group, promoting an electron withdrawal from the complexation sites and (iii) a triple bond, serving as an anchoring group. Although the synthesis of amine **3** was previously described [26], we used an alternative method (Scheme 1). 

4-Nitrophenylsulfonylchloride was reacted with methylamine in water in the presence of pyridine providing **1** in almost quantitative yield. The propargyl group was introduced to the molecule via alkylation with propargyl bromide in the presence of base resulting in the formation of **2**. The nitro group was reduced using tin(II) chloride in ethanol, leaving the triple bond intact. The ureido group, serving as a complexation site, was formed in the reaction of the amino group of **3** with phenyl isocyanate providing compound **4** as orange crystals suitable for single crystal X-ray diffraction analysis. Apart from the structure approval, the crystallography confirmed the L-shape of **4**, typical for sulfonamides. The hydrogen-binding capability of the ureido moiety is suggested by a non-covalently bound molecule of dimethylsulfoxide (DMSO).

**Scheme 2:**
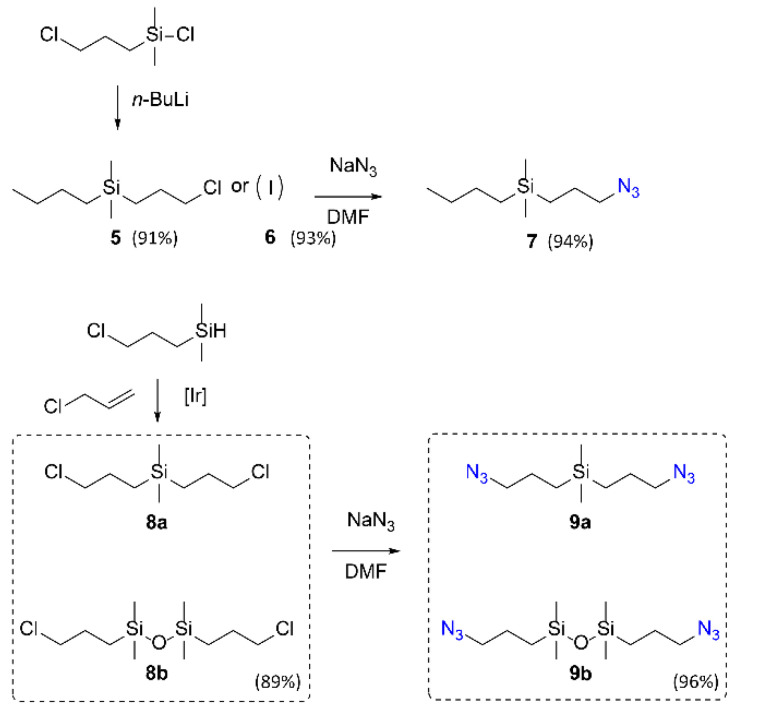
Synthesis of azide carriers **7**, **9a** and **9b**.

The carriers for attachment of the receptor moieties were based on carbosilane molecules differing in the number of azido groups within the structure (Scheme 2). The respective starting azides were prepared according to published procedures [27,28,29], except **7** and **9a**. The synthesis of compound **7** started with reaction of butyllithium with chloro(3-chloropropyl)dimethylsilane, giving **5** in 91% yield. The silane **5** was either converted to iodo-derivative **6** by Finkelstein reaction or directly reacted with sodium azide to obtain **7**. The compound **9a** was obtained in two steps from (3-chloromethyl)dimethyl silane. Its Ir-catalyzed addition to allylchloride gave **8a** together with **8b** in approx. 1:1 ratio. The subsequent treatment of the obtained mixture with sodium azide under the conditions of nucleophilic substitution yielded a mixture of the corresponding azides **9a** and **9b**.

**Scheme 3:**
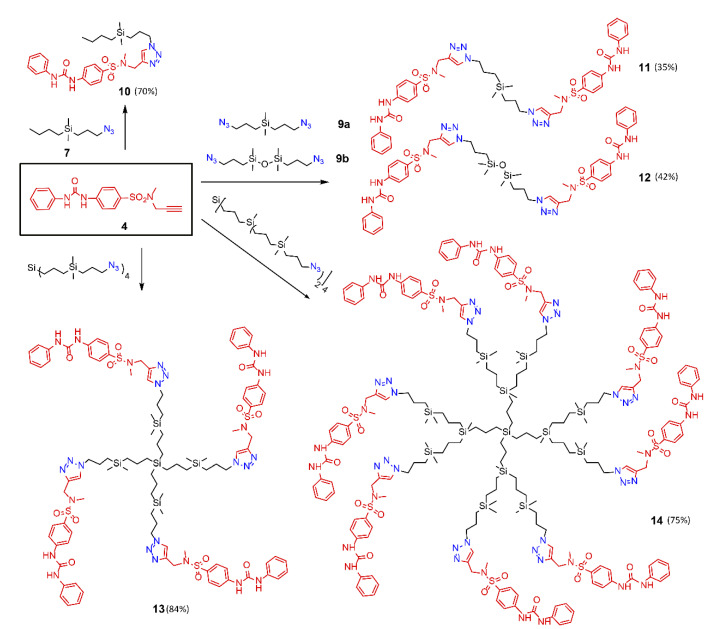
Synthesis of receptors **10**–**14**.

The prepared azide-derivatized carriers were reacted with intermediate **4** providing receptors **11**–**14** (Scheme 3). In all cases, the copper(I) catalyzed click reaction was employed [30,31]. In the case of bivalent carriers, the mixture of **9a** and **9b** was subjected to click reaction with **4.** The resulting mixture of receptors **11** and **12** in 1:1 ratio was easily separable by preparative thin layer chromatography (TLC).

The insight into the complexation behavior of prepared receptors when exposed to anions was provided by UV-Vis and/or NMR titration experiments [32,33]. To determine the selectivity and efficiency of the concerned complexation site, the monovalent receptor **10** offering the simplest complexation mode with 1:1 stoichiometry was titrated with a series of anions in the form of their tetrabutylammonium (TBA^+^) salts using ^1^H-NMR technique; the association constants [34] with higher uncertainty were specified also by UV-Vis titration. (Table 1).

The influence of binding site multiplication on anion complexation was studied on the series of receptor stock solutions with the same concentration of binding sites. Based on the above-mentioned preliminary study, the ^1^H-NMR titration experiments were performed with TBA^+^H_2_PO_4_^−^, as a strongly bound anion, and TBA^+^Cl^−^, as a weakly bound one. All receptors showed significant complexation-caused shifts of signals in ^1^H-NMR spectra, which were used to construct binding isotherms. In the case of bivalent receptors **11** and **12**, the binding isotherms did not have smooth shapes (see Supporting Material, Figures S52 and S53), revealing the presence of multiple ongoing processes. The signals of N*H* groups were located unexpectedly high (10.60 and 10.14 for **12,** compare to 9.43 and 9.06 for **10**) and shifted with dilution; with 10-fold dilution the N*H* signals of **12** moved to 9.36 and 9.19, respectively. The receptor **11** showed similar behavior. On the other hand, in the case of receptors **10, 13,** and **14** the dilution-induced changes in respective ^1^H-NMR spectra were negligible.

**Figure 1:**
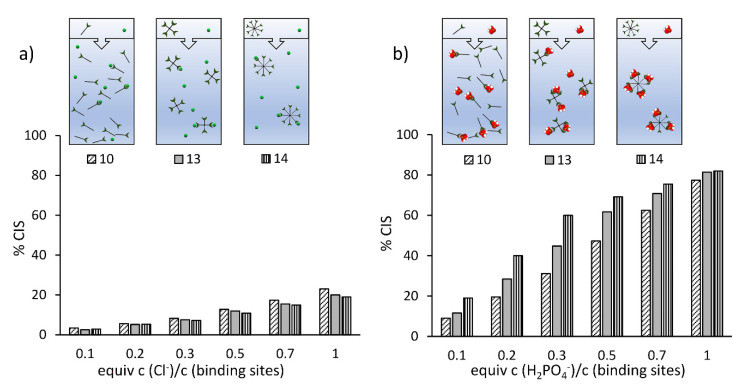
Comparison of complexation progress in DMSO solutions of receptors **10**, **13** and **14** with respect to concentration of binding sites with (**a**) TBA^+^Cl^−^, shift of N*H* (H at N15 in the crystal structure, Figure S64), (**b**) TBA^+^H_2_PO_4_^−^, shift of Ar*H* (H at C3/5 in the crystal structure, Figure S64).

As the complexation constants of systems with higher stoichiometry are not conveniently available, individual systems were compared considering the progress of complexation (expressed as percentage of maximal complexation induced shift (*CIS*)) at the same level of added anion with respect to concentration of available binding sites. The value of *CIS* for strong dihydrogen phosphate complexes was easily determined as a shift value reached in equilibrium and was similar for all the tested compounds. For weaker complexes with chloride, the value of *CIS* was approximated based on the calculation of association constant for **10**/Cl^−^ and adjusted to 600 Hz for N*H* signal (Figure 1).

To further investigate the given systems, we used a competitive approach recently introduced by Haav et al. [35]. The stock solutions were prepared, containing **10**/**13,** and **10**/**14**, with equal amount of monovalent and dendrimer-bound complexation sites, respectively. These mixtures were titrated with an anion according to the standard procedure. The complexation properties of both involved receptors were compared within the same titration experiment, excluding many of the possible uncertainty sources. The shifts of selected signals belonging to receptor **10** in the mixture were followed during the titration experiment and the results were compared to the receptor responses observed during standard titration.

**Figure 2:**
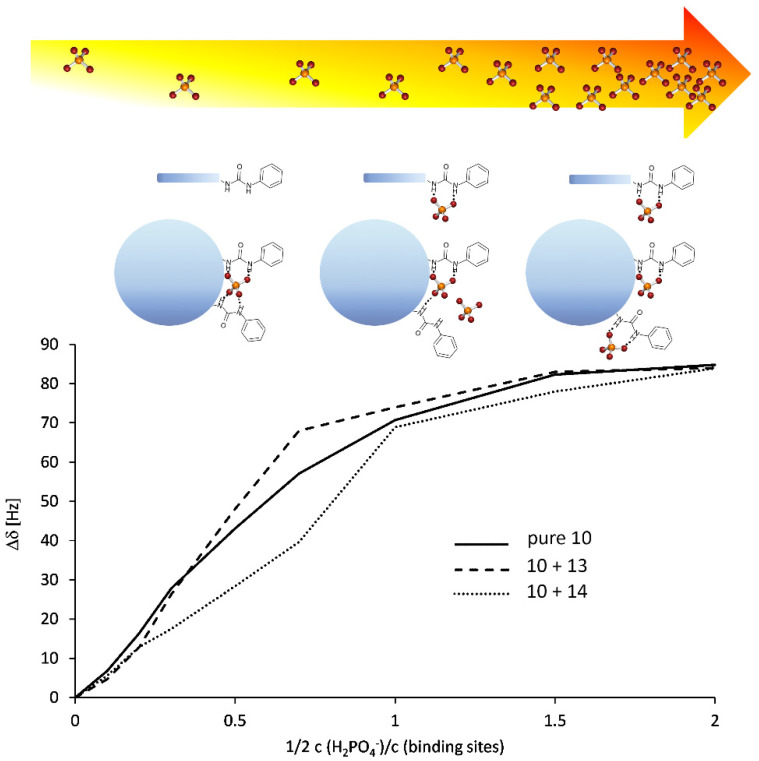
Comparison of H_2_PO_4_^−^ binding isotherm of pure **10** with the corresponding binding isotherms of **10** in the presence of **13** and **14,** respectively (shift of Ar*H*; H at C3/5, Figure S64). For clarity, lines are used to connect the experimental data points.

In the case of weakly bound chloride, the differences between curves measured for **10** alone and **10** in the mixture with **13** or **14** are negligible. When **10** is titrated by dihydrogen phosphate in the presence of **13** or **14** (Figure 2), notable differences can be found when compared to sole **10**. At lower concentrations of H_2_PO_4_^−^, **10** seems to have less than one half of the whole amount of anions available for complexation. At higher levels of a guest, the receptor **10** returns to the binding isotherm obtained by the standard methodology or even higher. The addition of more H_2_PO_4_^−^, beyond the concentration of available binding sites leads to the same equilibria in all the monitored cases.

## 3. Discussion

The receptor **10** showed a remarkable selectivity toward dihydrogen phosphate anion and carboxylates over halides, as expressed by two orders of magnitude difference in binding constants. Addition of the second complexation site to the structure of receptor and formation of bivalent hosts **11** and **12** significantly altered the complexation behavior. From the high positions of N*H* signals in ^1^H-NMR spectra, which changed with dilution, the intermolecular hydrogen bonding can be deduced. Such behavior cannot be attributed to the presence of disiloxane oxygen in **12**, as the receptor **11** formed similar aggregates. The inclusion of two urea moieties into the structure of **11** or **12** probably enables the development of enthalpically preferred folded structures, where both the binding sites are involved in the intramolecular interaction. As the anion complexation has to compete with this self-association, the bivalent receptors **11** and **12** were excluded from further studies.

The dilution-induced changes in ^1^H-NMR spectra of **10**, **13,** and **14** were negligible from structural reasons; in monovalent **10**, these interactions should be much weaker than in **11** or **12**, due to the absence of the second interacting moiety. In the case of **13** and **14**, the steric hindrance plays the role preventing the molecules from mutual interaction. Therefore, all the changes in ^1^H-NMR signal positions during titration experiments can be attributed to complexation.

The comparable changes of the signal positions for receptors **10**, **13** and **14** at low concentrations of dihydrogen phosphate anion indicate that the role of lower homogeneity of binding sites or salt accumulation in the localized areas is negligible and the establishment of equilibria is quick (Figure 1). In the case of **14,** a significantly different behavior can be observed compared to **10**; after addition of 0.1 equiv. of H_2_PO_4_^−^, the % *CIS* corresponds to 20% binding sites occupancy. This implies that until the concentration of anion reaches one half of the available complexation sites, two sites cooperate on binding of one anion. This effect is not so pronounced for the dendrimer **13**, but the stoichiometry of the H_2_PO_4_^−^ complexation also exceeds the 1:1 binding site:anion ratio. This behavior is evident, although the cooperation between two binding sites comprising the sulfonamide group conjugated to urea is reported to be quite unusual [25]. The assumption of cooperative binding of dihydrogen phosphate in **13** and **14** is supported by the shape of binding isotherms (see Supporting Material, Figure S43) exhibiting a plateau after addition of two or four receptor concentration equivalents of H_2_PO_4_^−^, respectively. Moreover, in the ^1^H-NMR spectra of **13** and **14**, the signals of triazole proton and the nearby protons move significantly with the complexation to the higher field, indicating an increased shielding caused by dendritic branches coming closer upon complexation. Naturally, no such shielding is visible in the case of **10** (Appendix A).

Considering the uncertainty in *CIS* determination, the titration of receptors **10**, **13** and **14** with TBA^+^Cl^−^ led to very similar results in all cases, regardless the count of binding sites per one molecule (Figure 1). The weakly bound chloride probably does not have a convenient shape and size for cooperative bonding to the dendritic receptors and no effects similar to those observed for dihydrogen phosphate can be followed. The same results were obtained by the competitive experiments; the binding isotherms for sole **10** did not differ much from those measured for **10** in the mixture with a dendritic receptor, confirming low influence of binding sites cumulation on chloride complexation.

On the contrary, in the case of dihydrogen phosphate at low loadings, the dendritic molecules offer a possibility of advantageous cooperative binding of one H_2_PO_4_^−^ anion to two branches. This is consistent with competitive study results, where complexation of H_2_PO_4_^−^ by **10** was initially hindered by the presence of dendritic molecule (Figure 2). This feature, although also present in **13**, is particularly pronounced in the case of **14**, which is in accordance with the titration data obtained for the sole receptors. At higher levels of a guest the complexation sites of the dendrimer offering possibility of 1:2 binding mode become saturated. Adding more anion, the dendritic complexes undergo a dissociation step, where the hydrogen bonds of a branch toward the anion are disrupted and another anion is bound forming 1:1 complex. Receptor **10** does not undergo such a process and profits from the dendritic receptor binding mode change, using more than a half of total present H_2_PO_4_^−^. When the concentration of H_2_PO_4_^−^ exceeds the concentration of available binding sites, both the monovalent and dendrimer-bound complexes are equally stable.

To regenerate the dendritic receptors, the methodology of nanofiltration was used. After addition of methanol, which destabilized the complexes and lowered the viscosity of feed solutions, the pressure of nitrogen was used to filtrate the mother liqueurs through the corresponding membranes; 1 kDa cut-off for **13** and 3 kDa for **14**, with 60–70% receptor recovery.

In conclusion, the (phenylureido)sulfonamidic moieties were successfully anchored to carbosilane carriers providing multivalent receptors for anions. Notwithstanding the bivalent receptors, which formed self-aggregates in DMSO solution, the multivalent host molecules proved to retain the selectivity and efficiency of monovalent ones. Moreover, the attachment to dendritic scaffold fits the binding sites into a suitable position for cooperative binding of H_2_PO_4_^−^ anion, particularly pronounced in the second generation of dendrimer. At low molar fractions of dihydrogen phosphate, a positive dendritic effect was observed due to advantageous formation of 2:1 complexes between anchored receptor moieties and dihydrogen phosphate. Despite the weak negative dendritic effect observed at H_2_PO_4_^−^ molar fractions between 0.5 and 1 caused by the change of binding mode, the overall affinity and selectivity of the binding sites is not affected by the anchoring. No dendritic effects were observed for weakly bound chloride and all binding sites work similarly, regardless the carrier. Such knowledge opens access to potent receptors of anions suitable for multiple use, considering the feasible recycling by nanofiltration.

## 4. Materials and Methods

### 4.1. General

The reagents were purchased from commercial sources and used without further purification. The starting carbosilane dendrimers were prepared by the standard iterative procedure [36,37]. n-Butyllithium solution (2.5 M in hexanes), bis(1,5-cyclooctadiene)diiridium(I) dichloride ([{IrCl(COD)}_2_]), (1*Z*,5*Z*)-cycloocta-1,5-diene (COD) were purchased from Merck KGaA. Chloro(3-chloropropyl)dimethylsilane was purchased from Gelest, Inc. The solvents used for synthesis and chromatography were purchased from commercial sources and distilled before use. Anhydrous solvents were dried by standard procedures; DCM was dried over calcium hydride, pyridine was stored above NaOH(s), and DMF was stored over molecular sieves. 

The ^1^H (400.1 MHz), ^13^C (100.6 MHz) and ^29^Si (79.5 MHz) NMR spectra were recorded using a Bruker Avance 400 spectrometer (Bruker Biospin, Rheinstetten, Germany) at 25 °C. Used solvents (DMSO-*d*_6_, chloroform-*d*) were stored over molecular sieves. The ^1^H and ^13^C-NMR spectra were referenced to the line of the solvent (δ/ppm; δ_H_/δ_C_: DMSO-*d*_6_, 2.50/39.52, chloroform-*d*, 7.26/77.16). The ^29^Si spectra were referenced to the line of external standard hexamethyldisilane (δ/ppm; −19.79). To assign all proton and carbon signals, a combination of 1D and 2D experiments (H,H-COSY, H,C-HSQC and H,C-HMBC) was used. The high-resolution mass spectra (HRMS) were measured on a MicrOtof III spectrometer (Bruker Daltonic, Bremen, Germany) with electrospray (ESI) and atmospheric pressure chemical ionization (APCI) source in positive or negative mode. For calibration of accurate masses, ESI-APCI Low Concentration Tuning Mix (Agilent, Santa Clara, CA, USA) was used. The samples were delivered into the ion source in methanol solution; the ionization in **14** had to be enhanced by addition of ammonium formate buffer. The FTIR analysis was performed on a Nicolet 6700 spectrometer (Thermo-Scientific, Waltham, MA, USA) connected with a GladiATR diamond ATR adapter (PIKE-Technologies, Madison, WI, USA), reflectance measurement, DTGS KBr detector, with following parameters: spectral range: 4000–400 cm^−1^, resolution: 4 cm^−1^, number of spectra accumulations: 64, apodization: Happ-Genzel. The spectra were collected and processed by Omnic 9 (Thermo-Scientific, Waltham, MA, USA) including baseline correction. Diffraction data were collected on a Bruker D8 VENTURE Kappa Duo PHOTON 100 CMOS with the monochromatic Cu-Kα radiation (Bruker AXS, Karlsruhe, Germany). The crystal structure of **4** was solved by charge flipping methods in Superflip [38] and refined by full-matrix least-squares on F^2^ values in Crystals [39]. All non-hydrogen atoms were refined anisotropically. All hydrogen atoms could be localized from electron density maps, but according to common practice, hydrogen atoms bonded to carbon were repositioned geometrically, initially refined with soft restraints and afterwards refined using riding constraints. Hydrogen atoms bonded to nitrogen were localized from electron density maps and refined with restrained geometry. Mercury [40] was used for structure visualization. The crystallographic data have been deposited in the Cambridge Crystallographic Data Centre as a supplementary publication. These data are provided free of charge by the joint Cambridge Crystallographic Data Centre and Fachinformationszentrum Karlsruhe Access Structures service www.ccdc.cam.ac.uk/structures (accessed on 12 August 2021). 

Nanofiltration: for purification and recycling of dendritic receptors, a solvent resistant stirred cell (Merck Millipore, Burlington, MA, USA) equipped with 1 kDa or 3 kDa MWCO regenerated cellulose ultrafiltration membrane disk (Merck Millipore) and sealed with fluorinated ethylene propylene (FEP) coated O-rings (Eriks, Rychnov nad Kněžnou, ČR) was used. The filtration was driven by nitrogen under a pressure of 4.5 bar. Solutions of complexes were diluted by methanol to 20 mL total volume and filtered to a target volume of 1 mL of retentate; this run was repeated five times. Evaporation of the retentate gave the dendritic receptor.

### 4.2. Determination of Complexation Constants

Complexation constants were measured in DMSO-*d*_6_ by standard ^1^H-NMR titration methodology. A solution of tetrabutylammonium salt of a selected anion (purchased from commercial sources, stored in a dry box) was gradually added in aliquots into a solution of a given receptor to reach at least a 1:5 ratio of binding group to anion. Concentrations of respective receptors were about 4 mmol/L and were kept constant during the titrations to avoid the effects of dilution. The corresponding complexation constants were calculated based on the analysis of binding isotherms obtained from the complexation induced shifts of N*H* or aromatic protons. For non-linear curve fitting of experimental data, the freely available software Bindfit [34] was used.

The competition experiments were carried out in the mixtures of receptors with concentration of binding sites of 4 mmol/L of monovalent **10** and 4 mmol/L of respective dendritic molecule **13** or **14**, i.e., the mixture contained 4-fold molar excess of **10** compared to **13** or 8-fold molar excess of **10** compared to **14**, respectively. The concentration of TBA^+^ salt of an anion was twofold higher than in the case of a sole receptor.

The UV-Vis titrations were performed in DMSO using double beam UV-1800 spectrophotometer Shimadzu. All UV spectra were taken in the wavelength region from 260 to 800 nm, with steps of 1 nm using cuvettes with pathlength of 1 mm. The stability constants of the resulting complexes were evaluated by employing the freeware program Bindfit using the whole parts of the absorption curves where the changes in absorbance were the most significant.

### 4.3. Synthesis

#### 4.3.1. Synthesis of Precursors

*N*-methyl-4-nitrobenzene sulfonamide **1**

*p-*Nitrophenylsulfonylchloride (1 g, 4.5 mmol) was added into a stirred solution of methylamine (0.4 mL, 40% aqueous solution) in pyridine (20 mL). The reaction was stirred for 2 days at ambient temperature. After this time period, the reaction mixture was poured into aqueous HCl (5 M, 100 mL) and the product was extracted to ethyl acetate (2 × 50 mL). The combined organic layers were dried over magnesium sulfate, filtered and the filtrate was evaporated to give a title compound as a yellow solid (0.9 g, 93% yield). 

^1^H-NMR (400 MHz, DMSO-*d_6_*): 8.43 (d, *J* = 8.8 Hz, 2H), 8.03 (d, *J* = 8.8 Hz, 2H), 1.98 (s, 3H) ppm. ^13^C-NMR (100 MHz, DMSO-*d*_6_): 150.2, 145.2, 128.7, 125.1, 29.1 ppm. HRMS (ESIneg) calc for [C_7_H_7_N_2_O_4_S]^−^: 215.0132 found 215.0136, [M-H]^−^.

*N*-methyl-*N*-propargyl-4-nitrobenzene sulfonamide **2**

*N*-methyl-4-nitrobenzene sulfonamide **1** (0.8 g, 3.6 mmol) was dissolved in 30 mL of acetonitrile and 0.6 g (4.3 mmol) of anhydrous potassium carbonate was added. The mixture was stirred for 0.5 h before propargyl bromide (0.6 mL, 4.3 mmol, 60% solution in toluene) was added. Resulting mixture was refluxed for 2 days. After cooling, the solvent was removed in vacuo. The residue was taken up with 50 mL of ethyl acetate, aqueous HCl (1 M, 200 mL) was added and the product was extracted into ethyl acetate (3 × 50 mL). The combined organic layers were dried over magnesium sulfate, filtered and the filtrate was evaporated to give a title compound as a yellow solid (0.3 g, 33% yield).

^1^H-NMR (400 MHz, DMSO-*d_6_*): 8.43 (d, *J* = 8.9 Hz, 2H), 8.08 (d, *J* = 8.9 Hz, 2H), 4.10 (d, *J* = 2.5 Hz, 2H), 3.11 (t, *J* = 2.5 Hz, 1H), 2.81 (s, 3H) ppm. ^13^C-NMR (100 MHz, DMSO-*d*_6_): 150.1, 142.4, 129.3, 124.5, 77.0, 76.2, 39.3 (from HSQC), 34.3 ppm. HRMS (APCIpos) calc for [C_10_H_11_N_2_O_4_S]^+^: 255.0434, found 255.0436, [M + H]^+^.

*N*-methyl-*N*-propargyl-4-aminobenzene sulfonamide **3**

*N*-methyl-*N*-propargyl-4-nitrobenzene sulfonamide **2** (0.3 g, 1.2 mmol) was suspended in ethanol (50 mL). Tin(II) chloride dihydrate (2.65 g, 12 mmol) was added and the reaction mixture was refluxed overnight. After cooling, the solvent was removed in vacuo, the residue was taken up with ethyl acetate (100 mL) and aqueous potassium hydroxide (5 M, 100 mL) was added. The product was extracted to ethyl acetate (2 × 50 mL). The combined organic layers were washed with brine (100 mL) and, after separation, dried over magnesium sulfate. After filtration, the solvent was evaporated to give a title compound (0.27 g, quantitative yield) as a yellow syrup.

^1^H-NMR (400 MHz, DMSO-*d*_6_): 7.39 (d, *J* = 8.7 Hz, 2H), 6.63 (d, *J* = 8.7 Hz, 2H), 6.04 (s, 2H), 3.87 (d, *J* = 2.5 Hz, 2H), 3.13 (t, *J* = 2.5 Hz, 1H), 2.62 (s, 3H) ppm. ^13^C-NMR (100 MHz, DMSO-*d*_6_): 153.2, 129.6, 120.9, 112.6, 77.4, 76.3, 39.3 (from HSQC), 34.1 ppm. HRMS (APCIpos) calc for [C_10_H_13_N_2_O_2_S]^+^: 225.0692, found 225.0695, [M + H]^+^.

*N*-propargyl-*N*-methyl-4-(phenylureido)benzene sulfonamide **4**

To a stirred solution of *N*-methyl-*N*-propargyl-4-aminobenzene sulfonamide **3** (0.27 g, 1.2 mmol) in dichloromethane phenylisocyanate (0.2 mL, 1.8 mmol) was added dropwise. The reaction mixture was stirred overnight and then poured into water. The product was extracted to chloroform and organic phase was dried over magnesium sulfate, filtered and the filtrate was evaporated. The crude product was filtered through the silica (20 g) where the impurities were eluted by ethyl acetate/hexane 1/4 (3 column volumes). The product was then eluted using ethyl acetate. After evaporation, the title compound (0.17 g, 42%) was obtained as a yellow slowly crystalizing syrup.

^1^H-NMR (400 MHz, DMSO-*d*_6_): 9.17 (s, 1H), 8.81 (s, 1H), 7.74–7.63 (m, 4H), 7.51–7.41 (m, 2H), 7.36–7.26 (m, 2H), 7.02–6.98 (m, 1H), 3.98 (d, *J =* 2.5 Hz, 2H), 3.16 (t, *J* = 2.5 Hz, 1H), 2.71 (s, 3H) ppm. ^13^C-NMR (100 MHz, DMSO-*d*_6_): 152.2, 144.2, 139.2, 128.9, 128.8, 128.6, 122.3, 118.5, 117.5, 76.9, 76.5, 39.4 (from HSQC), 34.2 ppm. IR: 3337; 3298; 3255; 3143; 3111; 3067; 3038; 2114; 1707; 1310; 1152 cm^−1^. HRMS (APCIpos) calc for [C_17_H_18_N_3_O_3_S]^+^: 344.1063 found 344.1068, [M + H]^+^, CCDC code 2092890.

Butyl(3-Chloropropyl)dimethylsilane **5**

Chloro(3-chloropropyl)dimethylsilane (2 g, 11.7 mmol) was dissolved in dry pentane (10 mL) under argon atmosphere in Schlenk tube and cooled to −78 °C. To this solution was added 2.5 M solution of n-butyllithium in hexane solution (5.14 mL, 12.8 mmol). The reaction mixture was stirred at cooling for 30 min. After this time period, the reaction mixture was warmed to room temperature, stirred overnight and then poured into ice-cold saturated aqueous NH_4_Cl. The aqueous layer was extracted twice with diethyl ether, the combined organic layers were washed twice with water, once with saturated aqueous NaCl and dried over anhydrous MgSO_4_. The volatiles were removed on the rotary evaporator and finally under high vacuum giving title compound (2.05 g, 91%) as a yellow liquid.

^1^H-NMR (400 MHz, chloroform-*d*): 3.49 (t, *J* = 7.1 Hz, 2H), 1.79–1.71 (m, 2H), 1.35–1.24 (m, 4H), 0.88 (t, *J* = 7.0 Hz, 3H), 0.61–0.57 (m, 2H), 0.53–0.49 (m, 2H), −0.02 (s, 6H) ppm. ^13^C-NMR (100 MHz, chloroform-*d*): 48.2, 27.9, 26.7, 26.2, 14.9, 13.9, 13.1, −3.4 ppm. ^29^Si-NMR (79 MHz, chloroform-*d*): 2.72 ppm.

Butyl(3-iodopropyl)dimethylsilane **6**

Mixture of butyl(3-chloropropyl)dimethylsilane **5** (1.00 g, 5.19 mmol) and sodium iodide (3.11 g, 20.75 mmol) in 30 mL of butan-2-one was heated to reflux for 2 days. The reaction mixture was then cooled to room temperature. The product was extracted to diethyl ether (2 × 30 mL), the extract filtered through silica gel and dried over anhydrous MgSO_4_. The volatiles were removed on the rotary evaporator and finally under high vacuum. Yield: 1.37 g (93 %) of a yellow liquid.

^1^H-NMR (400 MHz, chloroform-*d*): 3.18 (t, *J* = 7.3 Hz, 2H), 1.85–1.77 (m, 2H), 1.33–1.23 (m, 4H), 0.88 (t, *J* = 7.1 Hz, 3H), 0.60–0.56 (m, 2H), 0.52–0.47 (m, 2H), −0.03 (s, 6H) ppm. ^13^C-NMR (100 MHz, chloroform-*d*): 29.1, 26.7, 26.2, 17.6, 15.0, 13.9, 11.5, −3.3 ppm. ^29^Si-NMR (79 MHz, chloroform-*d*): 2.17 ppm.

Butyl(3-azidopropyl)dimethylsilane **7**

Butyl(3-chloropropyl)dimethylsilane **5** (1 g, 5.20 mmol) and sodium azide (0.674 g, 10.37 mmol) in 20 mL of DMF were heated to 90 °C for 12 h. The reaction mixture was allowed to cool to room temperature. The crude product was passed twice through a short column filled with silica gel using diethyl ether as an eluent. The resulting solution was dried over anhydrous MgSO_4_. The solvent was removed on the rotary evaporator and the rest of volatiles under high vacuum giving the title compound (0.97 g, 94%) as a colorless liquid.

^1^H-NMR (400 MHz, chloroform-*d*): 3.21 (t, *J* = 7.1 Hz, 2H), 1.61–1.53 (m, 2H), 1.29–1.22 (m, 4H), 0.87 (t, *J* = 7.0 Hz, 3H), 0.54–0.47 (m, 4H), −0.0 (s, 6H) ppm. ^13^C-NMR (100 MHz, chloroform-*d*): 54.7, 26.7, 26.2, 23.8, 14.9, 13.9, 12.5, −3.4 ppm. ^29^Si-NMR (79 MHz, chloroform-*d*): 2.74 ppm.

bis(3-Chloropropyl)dimethylsilane **8a** and bis(3-chloropropyl)dimethyldisiloxane **8b**

A mixture of allylchloride (0.56 g, 7.32 mmol), [{IrCl(COD)}_2_] and COD was stirred at 40 °C for 10 min in a reaction flask equipped with a reflux condenser. At this temperature, (3-chloropropyl)dimethylsilane (1 g, 7.32 mmol) was added dropwise. The reaction mixture is further stirred for next 10 h at 40 °C and then passed through short column of silica gel with dichloromethane as an eluent. Solvents and unreacted substrates were evaporated using rotary evaporator giving the 1:1 mixture of **8a** and **8b** (1.48 g, 89%) as colorless liquid.

Data for **8a**

^1^H-NMR (400 MHz, chloroform-*d*): 3.51 (t, *J* = 6.7 Hz, 4H), 1.82–1.71 (m, 4H), 0.65–0.60 (m, 4H), 0.01 (s, 6H) ppm. ^13^C-NMR (100 MHz, chloroform-*d*): 48.1, 27.7, 12.9, −3.5 ppm. ^29^Si-NMR (79 MHz, chloroform-*d*): 3.25 ppm. NMR data for **8b** are in accordance with literature [35].

bis(3-azidopropyl)dimethylsilane **9a** and bis(3-azidopropyl)dimethyldisiloxane **9b**

Mixture of **8a** and **8b** (1.48 g) from previous reaction and sodium azide (1.77 g, 11.82 mmol) in 30 mL of DMF was heated at 90 °C for 12 h. The reaction mixture was allowed to cool to room temperature. The crude product was passed twice through a short column filled with silica gel using diethyl ether as an eluent. Resulting solution was dried over anhydrous MgSO_4_. The solvent was removed on the rotary evaporator and the rest of volatiles under high vacuum giving the 1:1 mixture of **9a** and **9b** (1.49 g 96%) as a colorless liquid.

Data for **9a**

^1^H-NMR (400 MHz, chloroform-*d*): 3.23 (t, *J* = 7.0 Hz, 4H), 1.63–1.54 (m, 4H), 0.58–0.53 (m, 4H), 0.01 (s, 6H) ppm. ^13^C-NMR (100 MHz, chloroform-*d*): 54.6, 23.7, 12.3, −3.5 ppm. ^29^Si-NMR (79 MHz, chloroform-*d*): 3.28 ppm.

Data for **9b**

^1^H-NMR (400 MHz, chloroform-*d*): 3.24 (t, *J* = 7.0 Hz, 4H), 1.68–1.57 (m, 4H), 0.58–0.54 (m, 4H), 0.07 (s, 6H) ppm. ^13^C-NMR (100 MHz, chloroform-*d*): 54.4, 23.2, 15.5, −0.4 ppm. ^29^Si-NMR (79 MHz, chloroform-*d*): 7.63 ppm.

#### 4.3.2. Preparation of Receptors—A General Procedure

In the 10 mL microwave vial was contacted an azide-carrier molecule with intermediate **4** (1.2 equivalents per azide group) in DMF (6 mL) in the presence of copper(I) iodide (0.5 equiv. per azide group) and DIPEA (10 equiv. per azide group). The vial was sealed and irradiated by microwaves for 1.5 h at 120 °C. After cooling, the contents of vial were poured to 1M HCl and the mixture was extracted with ethyl acetate (3 × 30 mL). The combined organic layers were dried over magnesium sulfate, filtered and the filtrate was evaporated giving the crude products.

Receptor **10**

The receptor **10** was prepared following the general procedure. The crude product was purified using preparative TLC (ethyl acetate/hexane 1/1) giving the receptor **10** as a yellow semi-solid in 70% yield.

^1^H-NMR (400 MHz, DMSO-*d*_6_): 9.43 (s, 1H), 9.06 (s, 1H), 8.02 (s, 1H), 7.71–7.66 (m, 4H), 7.48 (d, *J* = 8.5 Hz, 2H), 7.29 (dd, *J* = 8.5, 7.3 Hz, 2H), 6.99 (t, *J* = 7.3 Hz, 1H), 4.26 (t, *J* = 7.1 Hz, 2H), 4.21 (s, 2H), 2.59 (s, 3H), 1.80–1.73 (m, 2H), 1.29–1.17 (m, 4H), 0.83 (t, *J* = 7.0 Hz, 3H), 0.47–0.43 (m, 2H), 0.41–0.36 (m, 2H), −0.07 (s, 6H) ppm. ^13^C-NMR (100 MHz, DMSO-*d*_6_): 152.25, 144.17, 141.56, 139.33, 128.82, 128.66, 127.96, 123.87, 122.21, 118.40, 117.60, 52.24, 44.96, 34.59, 25.97, 25.55, 24.64, 14.20, 13.67, 11.36, −3.50 ppm. ^29^Si-NMR (79 MHz, DMSO-*d*_6_): 2.69 ppm. HRMS (ESIpos) calc for [C_26_H_39_N_6_O_3_SSi]^+^: 543.2568, found 543.2572, [M + H]^+^, calc for [C_26_H_37_N_6_O_3_SSiNa]^+^: 565.2387 found 565.2388, [M + Na]^+^.

Receptor **11**

The receptor **11** was prepared form the reaction mixture of intermediates **9a** and **9b** following the general procedure. The crude product was purified using preparative TLC (ethyl acetate/hexane 1/3) giving the receptor **11** as a more polar fraction in the form of a yellow syrup in 35% yield.

^1^H-NMR (400 MHz, DMSO-*d*_6_): 9.80 (s, 2H), 9.42 (s, 2H), 7.99 (s, 2H), 7.68 (s, 8H), 7.49 (d, *J* = 8.5 Hz, 4H), 7.28 (dd, *J* = 85, 7.3 Hz, 4H), 6.98 (t, *J* = 7.3 Hz, 2H), 4.24 (t, *J* = 7.1 Hz, 4H), 4.20 (s, 4H), 2.59 (s, 6H), 1.76–1.68 (m, 4H), 0.40–0.36 (m, 4H), −0.07 (s, 6H) ppm. ^13^C-NMR (100 MHz, DMSO-*d*_6_): 152.5, 144.5, 141.6, 139.7, 128.8, 128.6, 128.3, 123.9, 122.0, 118.4, 117.6, 52.2, 45.0, 34.6, 24.5, 11.1, −3.7 ppm. ^29^Si-NMR (79 MHz, DMSO-*d_6_*): 3.13 ppm. IR: 3336; 3287; 3135; 2952; 2927; 1713; 1310; 1155 cm^−1^. HRMS (ESIpos) calc for [C_42_H_52_N_12_O_6_S_2_SiNa]^+^: 935.3235, found 935.3233, [M + Na]^+^, calc for [C_42_H_53_N_12_O_6_S_2_Si]^+^: 913.3416, found 913.3412, [M + H]^+^.

Receptor **12**

The receptor **12** was prepared form the reaction mixture of intermediates **9a** and **9b** following the general procedure. The crude product mixture was purified using preparative TLC (ethyl acetate/hexane 1/3) giving the receptor **12** as a less polar fraction in the form of orange syrup in 42% yield.

^1^H-NMR (400 MHz, DMSO-*d*_6_): 10.60 (s, 2H), 10.14 (s, 2H), 8.01 (s, 2H), 7.72–7.66 (m, 8H), 7.51 (d, *J* = 8.5 Hz, 4H), 7.27 (dd, *J* = 8.5, 7.3 Hz, 4H), 6.96 (tt, *J* = 7.3, 1.2 Hz, 2H), 4.27 (t, *J* = 7.1 Hz, 4H), 4.20 (s, 4H), 2.58 (s, 6H), 1.80–1.73 (m, 4H), 0.42–0.37 (m, 4H), 0.00 (s, 12H) ppm. ^13^C-NMR (100 MHz, DMSO-*d*_6_): 152.69, 144.70, 141.61, 139.82, 128.75, 128.64, 128.10, 123.94, 121.87, 118.16, 117.36, 51.99, 44.97, 34.61, 24.06, 14.41, 0.20 ppm. ^29^Si-NMR (79 MHz, DMSO-*d*_6_): 8.09 ppm. IR: 3341; 3284; 3196; 2950; 2926; 1710; 1309; 1153 cm^−1^. HRMS (ESIpos) calc for [C_44_H_59_N_12_O_7_S_2_Si_2_]^+^: 987.3604, found 987.3598, [M + H]^+^, calc for [C_44_H_58_N_12_O_7_S_2_Si_2_Na]^+^: 1009.3423 found 1009.3418, [M + Na]^+^.

Receptor **13**

The receptor **13** was prepared following the general procedure, except form extraction. Due to low solubility of **13** in majority of organic solvents, after pouring the reaction mixture to aq. HCl, the solids were filtered off. The filtrate was depleted and the filtration obtained solids were sonicated in DMSO for 15 min. The insoluble matter was filtered off and to the filtrate was added Chelex^®^ 100 sodium (Sigma Aldrich) to remove the residual Cu. After stirring overnight, Chelex^®^ was removed by filtration and the solution was concentrated by lyophilization. The crude product was purified by nanofiltration (MWCO 1 kDa, MeOH) giving the receptor **13** as a brownish syrup in 84% yield. The receptor can be recovered from the complexes with anions by nanofiltration (MWCO 1 kDa, MeOH) in 60–70% yields.

^1^H-NMR (400 MHz, DMSO-*d*_6_): 9.16 (s, 4H), 8.80 (s, 4H), 8.01 (s, 4H), 7.73–7.60 (m, 16H), 7.46 (d, *J* = 8.0 Hz, 8H), 7.28 (dd, *J* = 8.0, 7.3 Hz, 8H), 6.99 (t, *J* = 7.3 Hz, 4H), 4.24 (t, *J* = 5.7 Hz, 8H), 4.19 (s, 8H), 2.58 (s, 12H), 1.77–1.73 (m, 8H), 1.31–1.22 (m, 8H), 0.52–0.48 (m, 16H), 0.39–0.35 (m, 8H), −0.09 (s, 24H) ppm. ^13^C-NMR (100 MHz, DMSO-*d*_6_): 152.1, 144.0, 141.7 (from HMBC), 139.1, 128.7, 128.6, 123.9, 122.2, 118.4, 117.6, 52.2, 44.9, 34.5, 24.5, 19.2, 18.0, 16.8, 11.5, −3.6 ppm. ^29^Si-NMR (79 MHz, DMSO-*d*_6_): 2.04 (4Si), 0.78 (1Si) ppm. IR: 3344; 3298; 3136; 2954; 2922; 1711; 1311; 1155 cm^−1^. HRMS (ESIpos) calc for [C_100_H_141_N_24_O_12_S_4_Si_5_]^+^: 2138.8907, found 2138.8898, [M + H]^+^.

Receptor **14**

The receptor **14** was prepared following the general procedure, except from extraction. Due to low solubility of **14** in majority of organic solvents, after pouring the reaction mixture to aq. HCl, the solids were filtered off. The filtrate was depleted and the filtration obtained solids were sonicated in DMSO for 15 min. The insoluble matter was filtered off and to the filtrate was added Chelex^®^ 100 sodium (Sigma Aldrich) to remove the residual Cu. After stirring overnight, Chelex^®^ was removed by filtration and the solution was concentrated by lyophilization. The crude product was purified by nanofiltration (MWCO 3 kDa, MeOH) giving the receptor **14** as a brownish syrup in 75% yield. The receptor can be recovered from the complexes with anions by nanofiltration (MWCO 3 kDa, MeOH) in 60–70% yields.

^1^H-NMR (400 MHz, DMSO-*d*_6_): 9.76 (s, 8H), 9.34 (s, 8H), 7.97 (s, 8H), 7.69–7.64 (m, 32H), 7.48 (d, *J* = 8.0 Hz, 16H), 7.26 (dd, *J* = 8.0, 7.2 Hz, 16H), 6.96 (t, *J* = 7.2 Hz, 8H), 4.22 (t, *J* = 5.7 Hz, 16H), 4.17 (s, 16H), 2.56 (s, 24H), 1.73 (br s, 16H), 1.29–1.25 (m, 24H), 0.48 (br s, 48H), 0.38–0.36 (m, 16H), −0.11 (s, 48H), −0.15 (s, 12H) ppm. ^13^C-NMR (100 MHz, DMSO-*d*_6_): 152.3, 144.3, 141.5, 139.4, 128.8, 128.7, 128.4, 123.8, 122.1, 118.3, 117.5, 52.2, 45.0, 34.5, 24.6, 19.2, 18.5, 18.3, 18.2, 18.0, 17.2, 11.6, −3.5, −4.9 ppm. ^29^Si-NMR (79 MHz, DMSO-*d*_6_): 2.00 (8Si), 1.03 (4Si) ppm, Si^0^ not detected. IR: 3349; 3275; 2950; 2913 1709; 1309; 1155 cm^−1^. HRMS (ESIpos) calc for [C_216_H_316_N_48_O_24_S_8_Si_13_Na_2_]^2+^: 2317.9790, 100%, found 2317.9803, [M + 2Na]^2+^, calc for [C_216_H_317_N_48_O_24_S_8_Si_13_Na]^2+^: 2306.9881, 100%, found 2306.9904 [M + H + Na]^2+^.

## Figures and Tables

**Table 1:** Association constants of **10** with a series of TBA^+^ salts of anions ^1^.

Anion	^1^H-NMR K_Ass_ [M^−1^] (Error %)	UV-Vis K_Ass_ [M^−1^] (Error %)
H_2_PO_4_^−^	5700 (36)	6100 (20)
AcO^−^	1300 (13)	1730 (7)
BzO^−^	2060 (14)	2200 (6)
HSO_4_^−^	190 (1.5)	-
Cl^−^	70 (4)	-
Br^−^	9 (0.8)	-

^1^ UV-Vis spectra were measured in DMSO, ^1^H-NMR in DMSO-*d*_6_.

## Data Availability

Data not contained within the article can be found in the attached Supplementary Material.

## Supplementary Materials

The following are available online: Spectral characterization of building blocks **7** and **9** and of receptors **10**–**14**, UV-Vis and ^1^H-NMR titration data, X-ray single crystal diffraction analysis.

## Sample Availability

Samples of the reported compounds are available from the authors.

## Appendix A

The triazole moiety can in some cases play a role of a donor of hydrogen bond. In the case of the receptor **10**, due to the presence of more efficient complexation site, this moiety did not exhibit any complexation induced changes in ^1^H-NMR spectra upon addition of anions. In the case of dendritic receptors **13** and **14**, upon addition of dihydrogen phosphate the signals of triazole proton and the nearby protons broaden and move to the higher field as the dendritic branches come closer in the 1:2 complexation mode (Figure S48, Supplementary Material).
