# Supplementary material for: Controlled Anchoring of (Phenylureido)sulfonamide-Based Receptor Moieties: An Impact of Binding Site Multiplication on Complexation Properties
Source: Molecules. 2021 Sep 18;26(18):5670. doi: 10.3390/molecules26185670 (PMC8468139; doi:10.3390/molecules26185670)
Supplement: Supplementary file 1 [file molecules-26-05670-s001.zip › molecules-1366588-supplementary.pdf]

Supporting information

Controlled Anchoring of (Phenylureido)sulfonamide-Based Receptor Moieties: An Impact of Binding Site Multiplication on Complexation Properties

Karolína Salvadori [1,2], Alena Krupková [1], Lucie Červenková Šťastná [1], Monika Müllerová [1], Václav Eigner [2], Tomáš Strašák [1] and Petra Cuřínová [1,*]

[1] Institute of Chemical Process Fundamentals of CAS, v.v.i., Rozvojová 135, 16502 Prague 6, Czech Republic; lottesalvadori@gmail.com (K.S.); krupkova@icpf.cas.cz (A.K.); stastna@icpf.cas.cz (L.Č.Š.); mullerovam@icpf.cas.cz (M.M.); strasak@icpf.cas.cz (T.S.)
[2] Department of Solid State chemistry, University of Chemistry and Technology Prague, Technická 5, 16828 Prague 6, Czech Republic; vaclav.eigner@vscht.cz
* Correspondence: curinova@icpf.cas.cz

Contents

Spectral characterization of building blocks 7 and 9 and of receptors 10-14 2
UV and ^{1}H NMR titration data 23
X-Ray single crystal diffraction analysis 32

Spectral characterization of building blocks 7 and 9 and of receptors 10-14.

Figure S1. Compound **7**, ^{1}H NMR (400 MHz, chloroform-*d*).

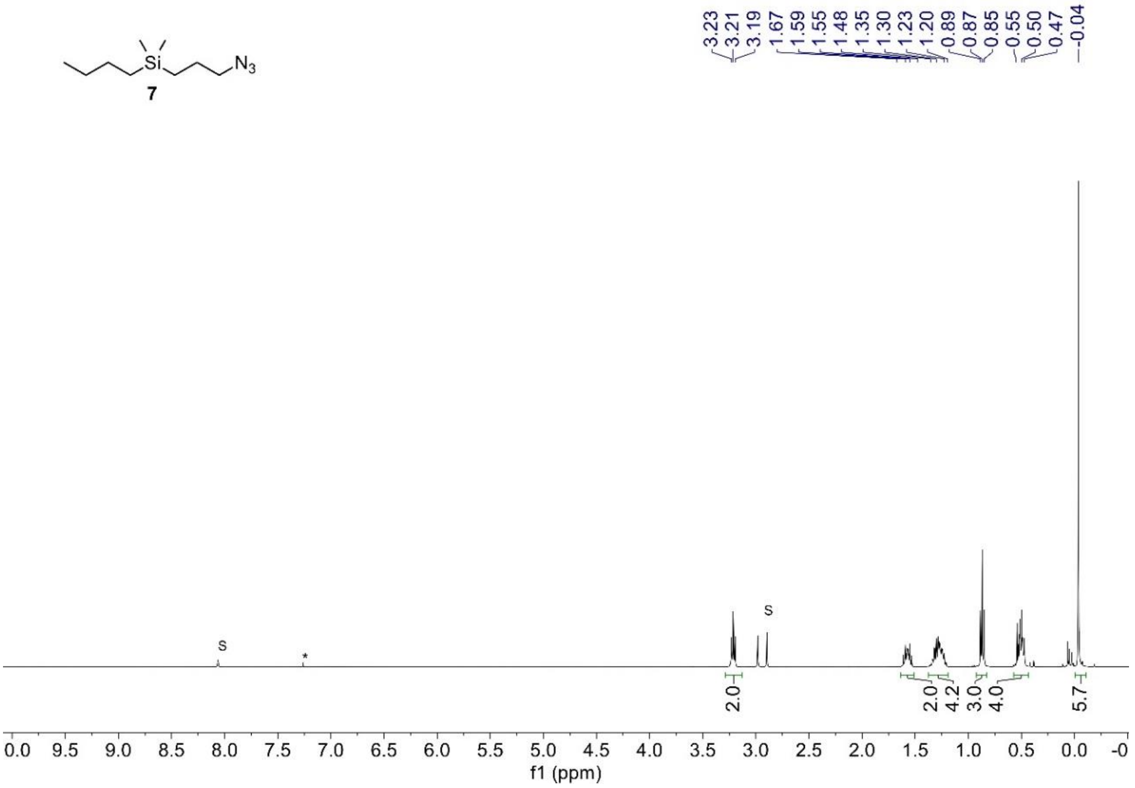

Figure S2. Compound **7**, ^{13}C {^{1}H} NMR (100 MHz, chloroform-*d*)

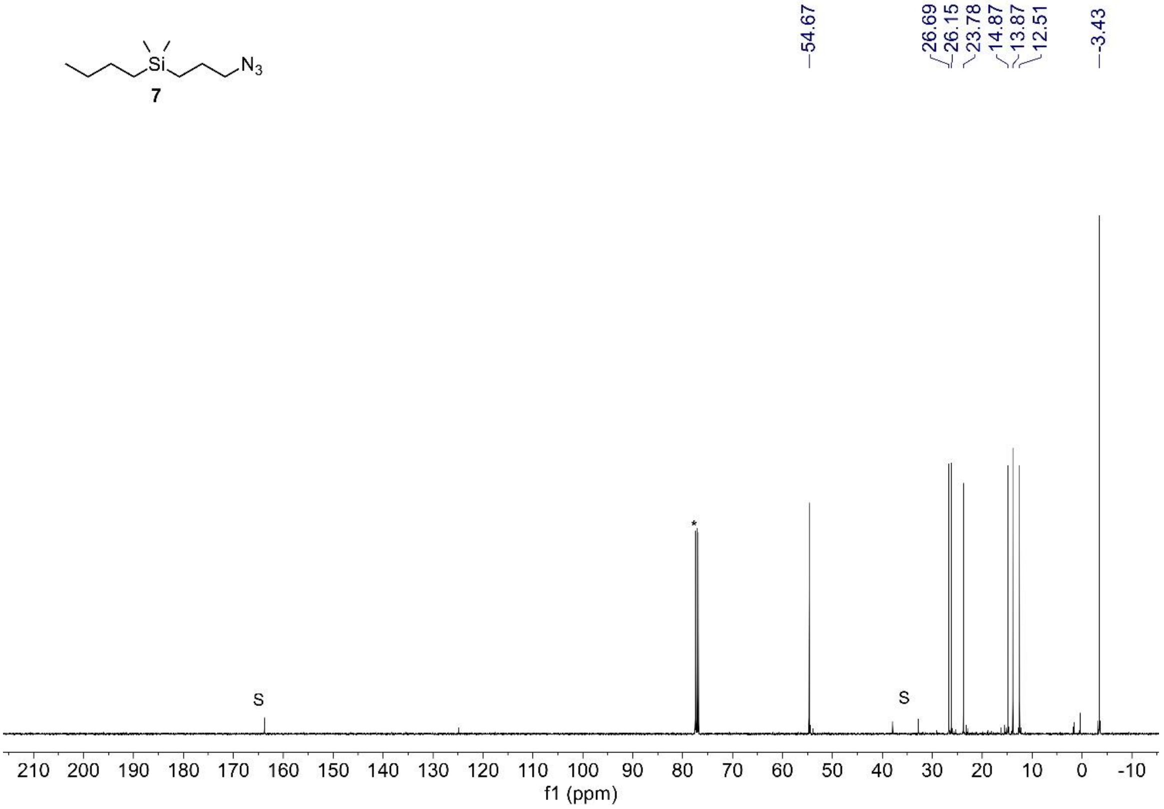

Figure S3. Compound **7**, ^{29}Si {^{1}H} NMR (79 MHz, chloroform-*d*).

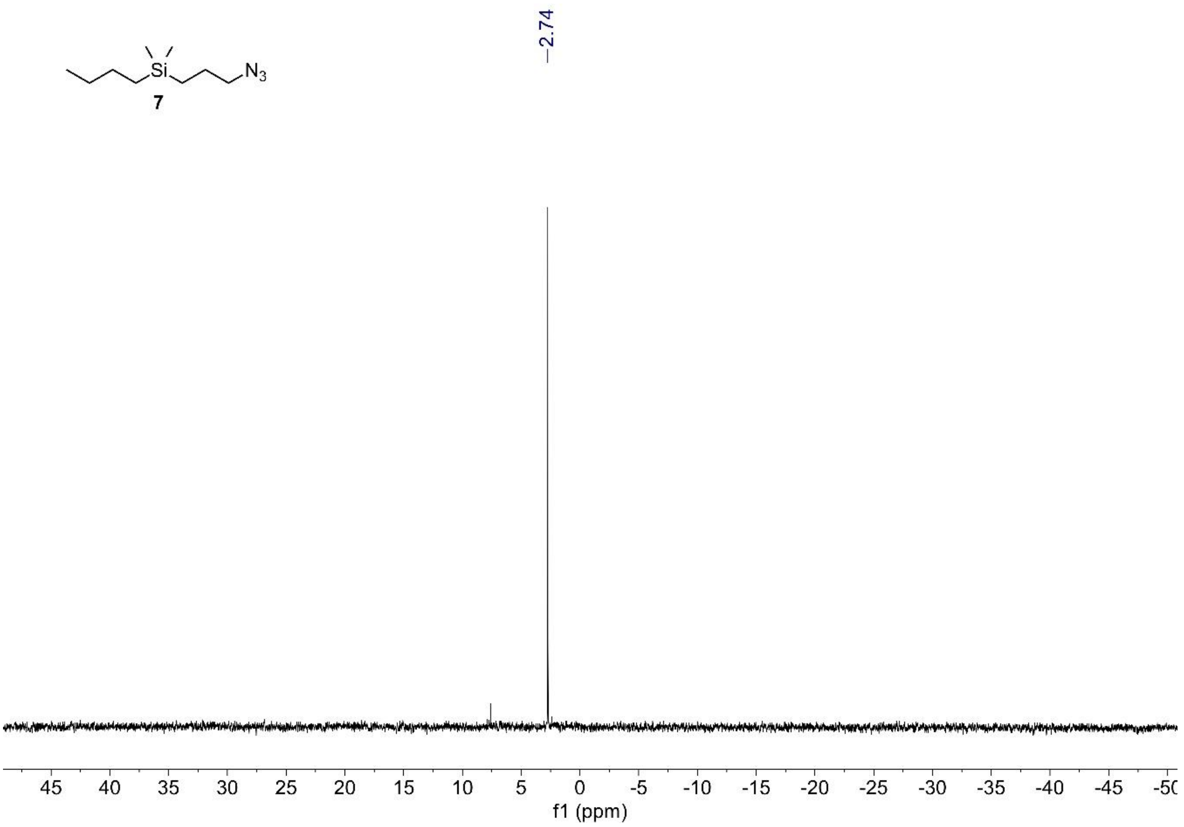

Figure S4. Compound **7**, ^{1}H-^{1}H COSY NMR, (400 MHz, chloroform-*d*$_6$).

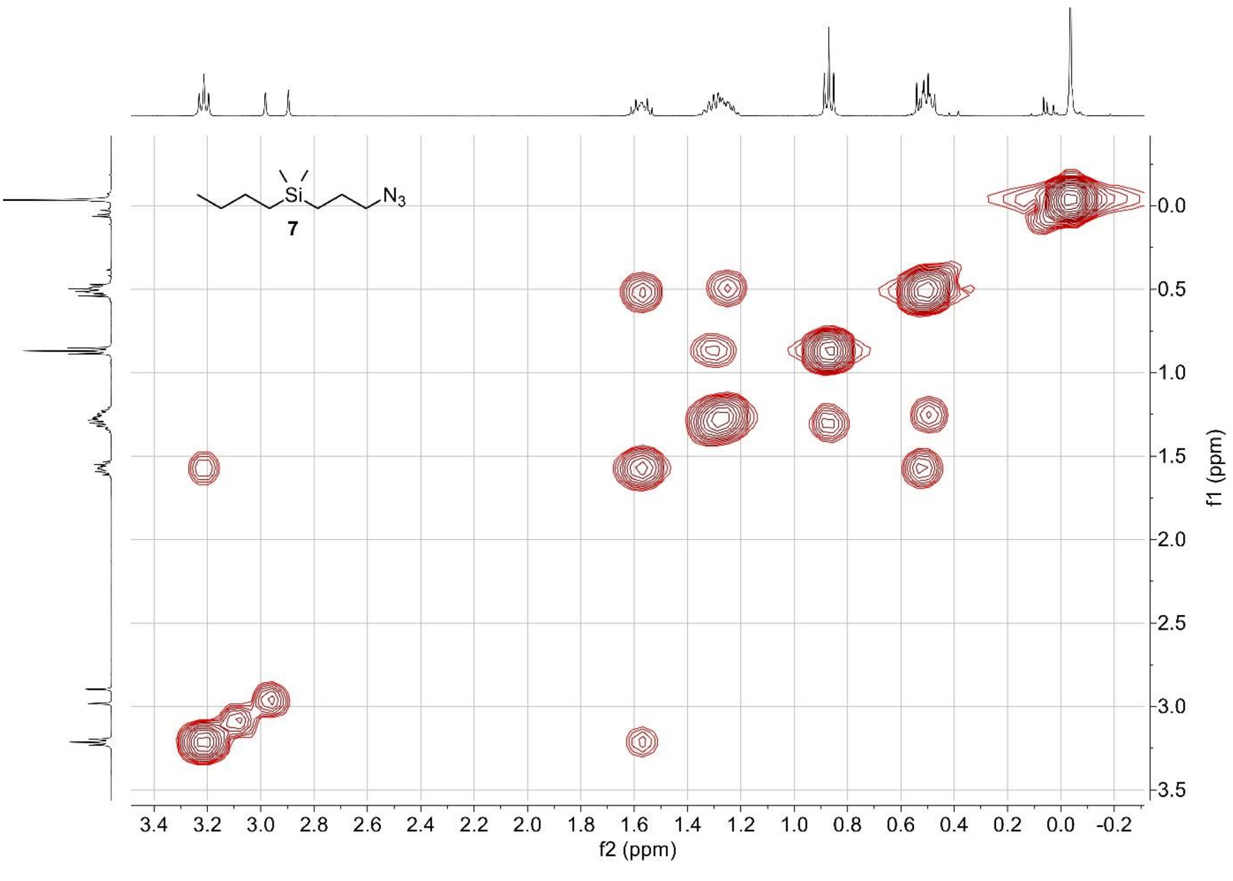

Figure S5. Compound **7**, ^{1}H-^{13}C HSQC NMR, (400/100MHz, chloroform-*d*).

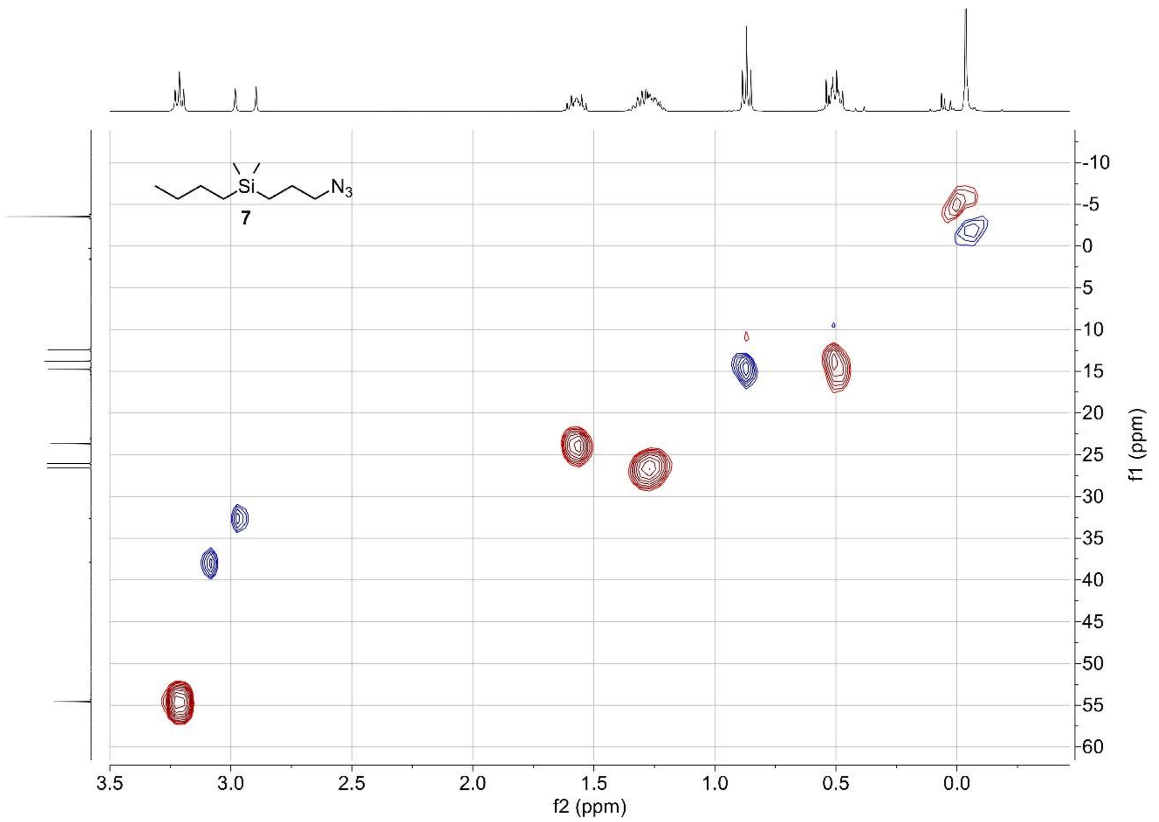

Figure S6. Mixture of compounds **9a, 9b**, ^{1}H NMR (400 MHz, chloroform-*d*).

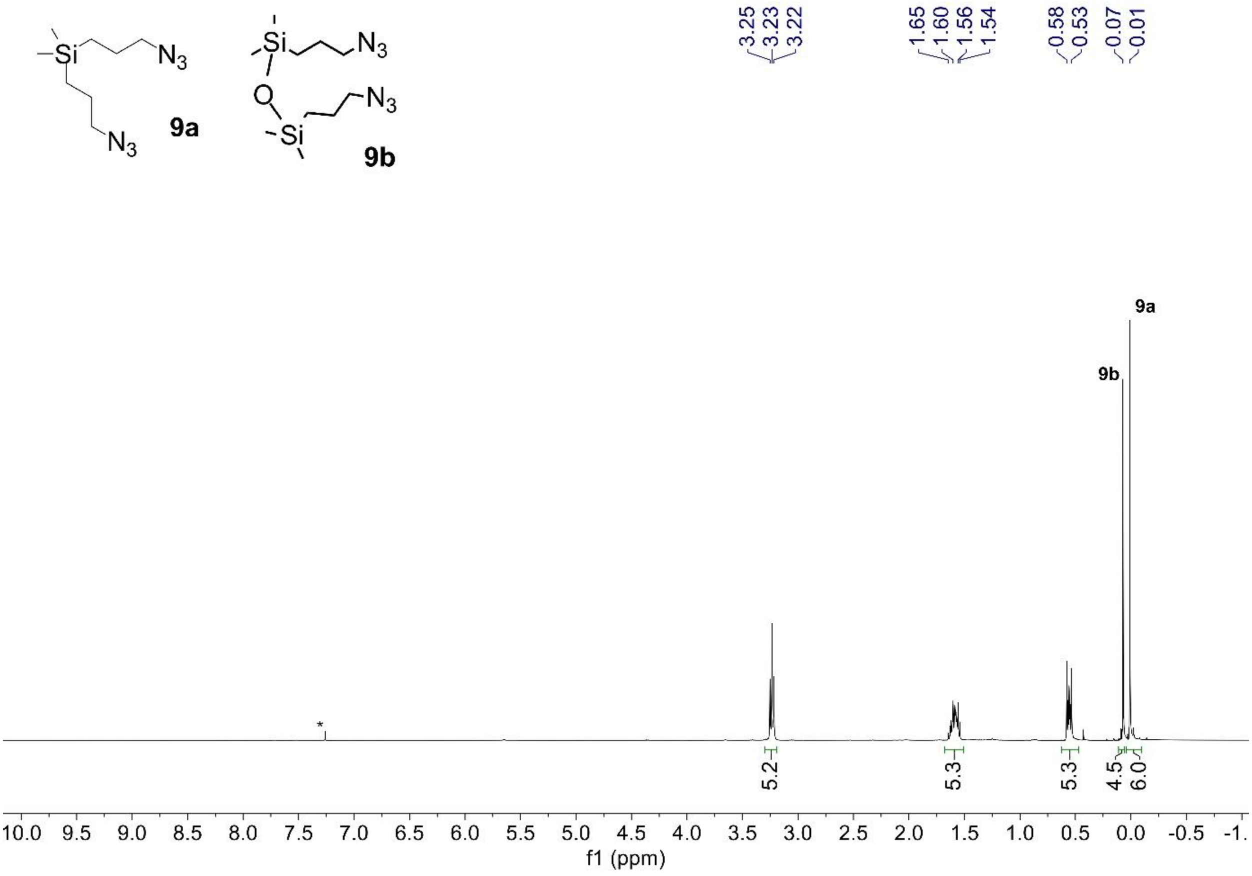

Figure S7. Mixture of compounds **9a, 9b**, $^{13}C\ \{^{1}H\}$ NMR (100 MHz, chloroform-*d*).

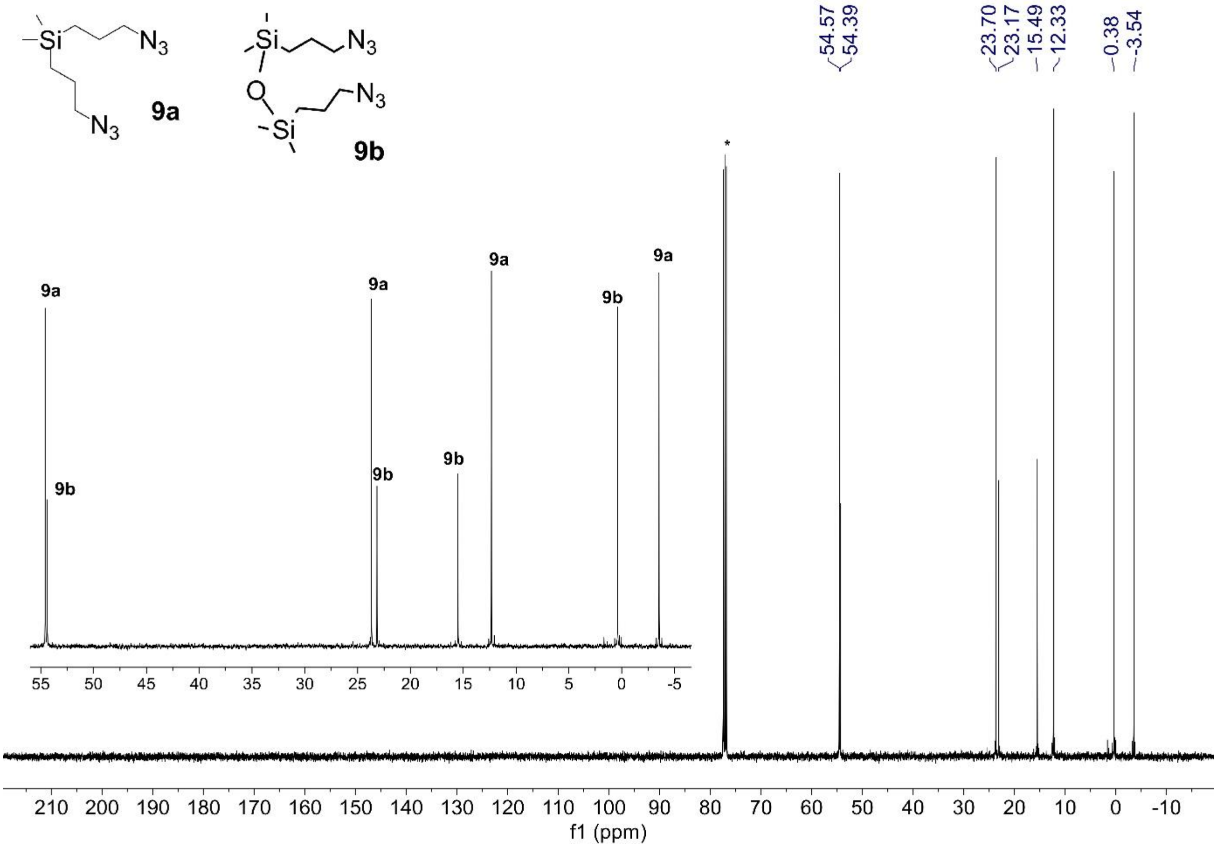

Figure S8. Mixture of compounds **9a, 9b**, $^{29}Si\ \{^{1}H\}$ NMR (79 MHz, chloroform-*d*).

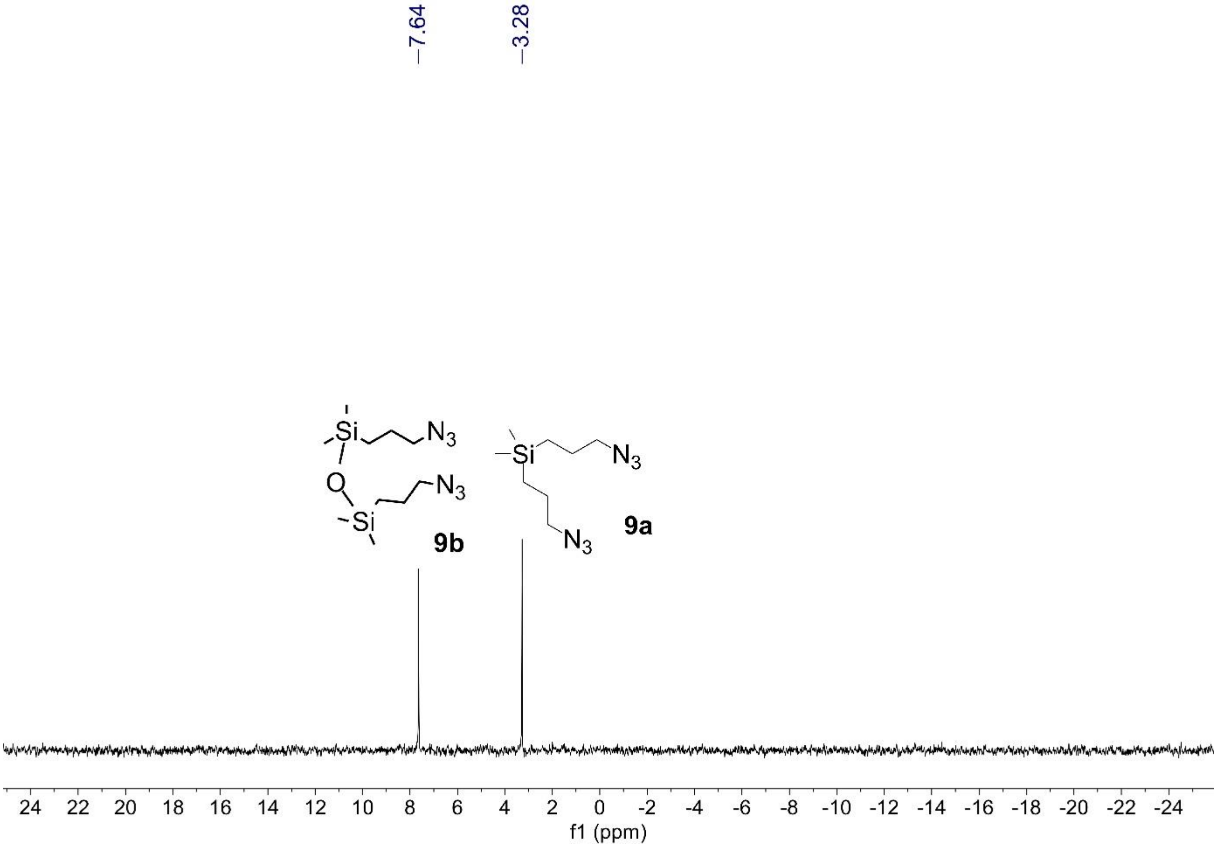

Figure S9. Mixture of compounds **9a, 9b**, ^{1}H-^{1}H COSY NMR (400 MHz, chloroform-*d*).

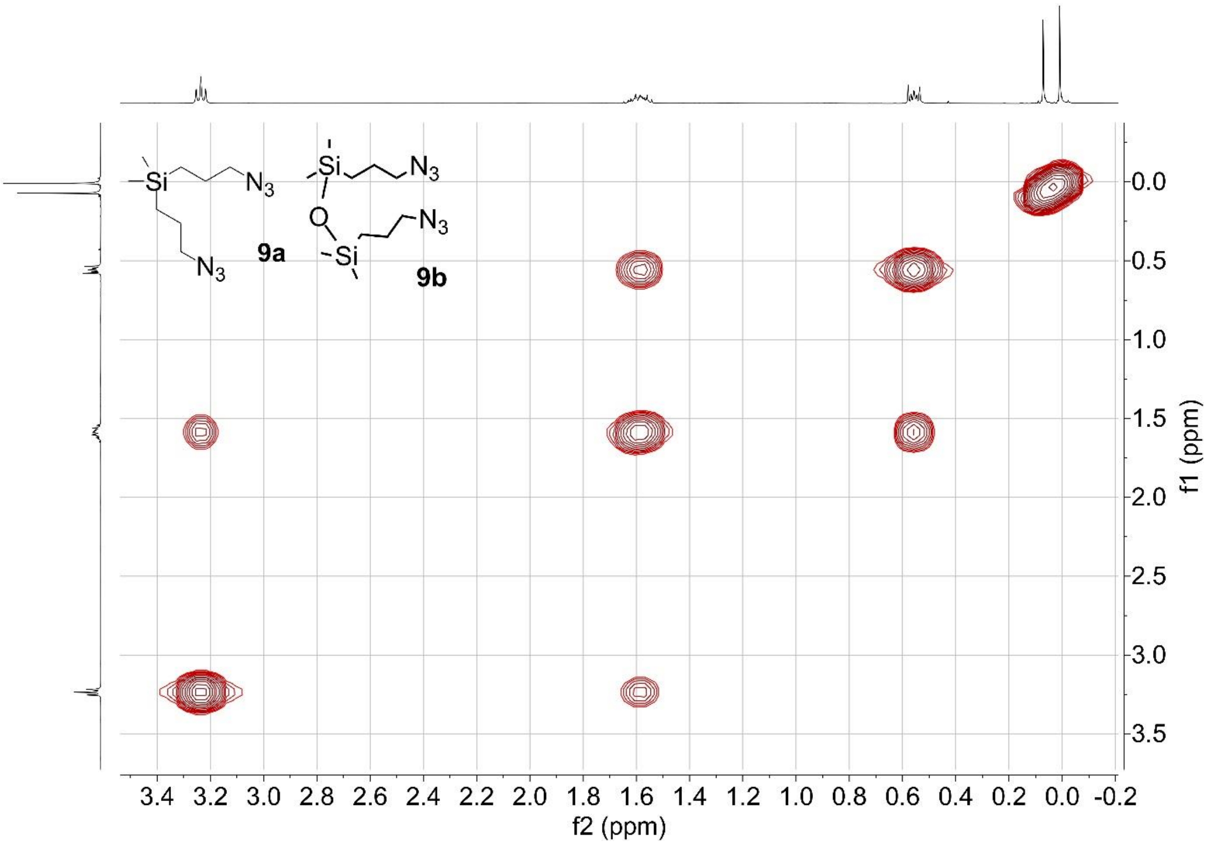

Figure S10. Mixture of compounds **9a, 9b**, ^{1}H-^{13}C HSQC NMR (400/100 MHz chloroform-*d*).

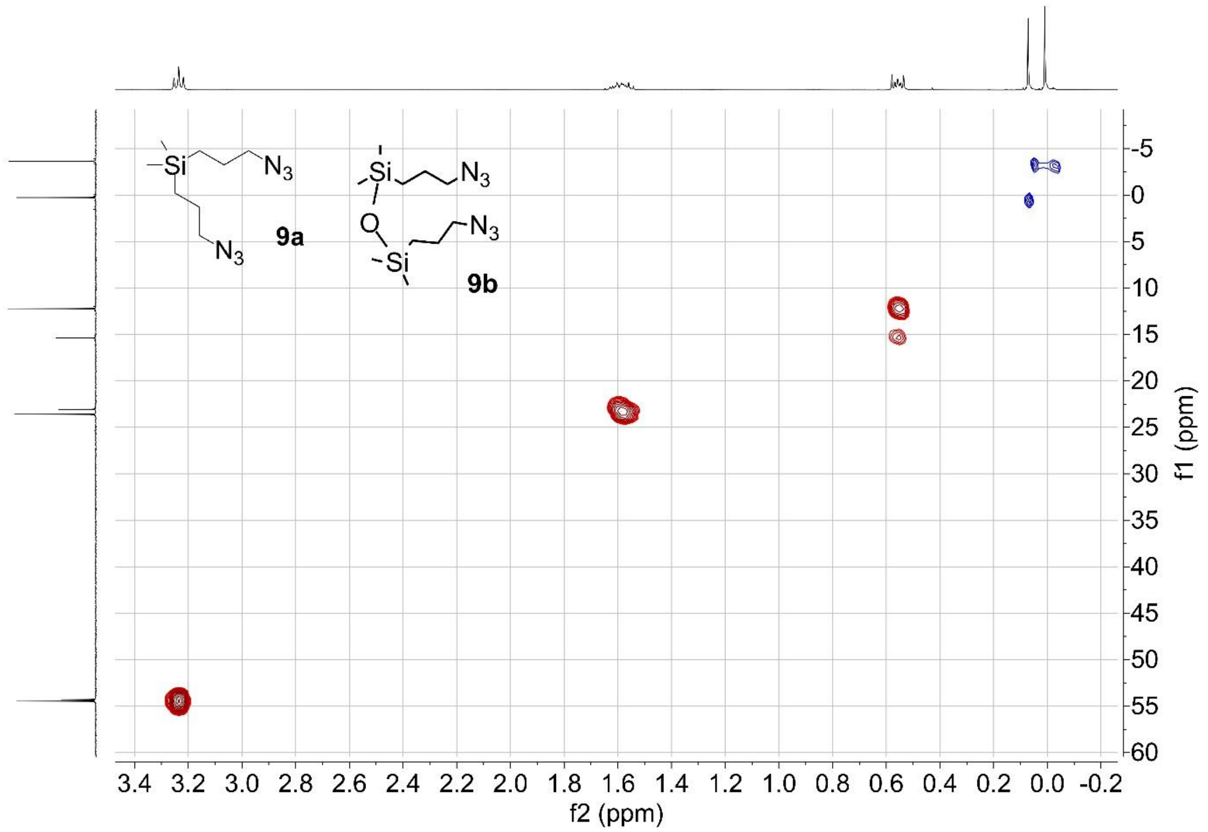

Figure S11. Compound **9b**, ^{1}H NMR (400 MHz, chloroform-*d*).

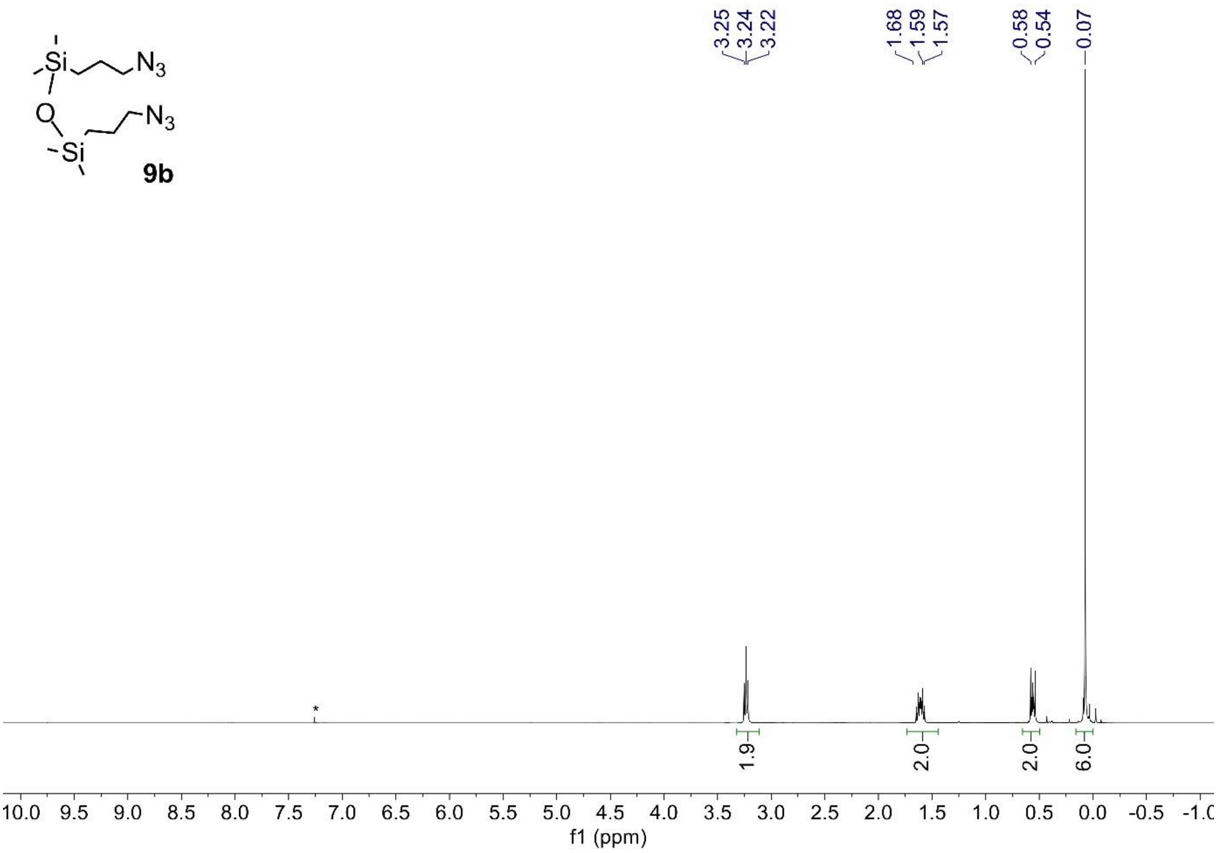

Figure S12. Compound **9b**, ^{13}C {^{1}H} NMR (100 MHz, chloroform-*d*).

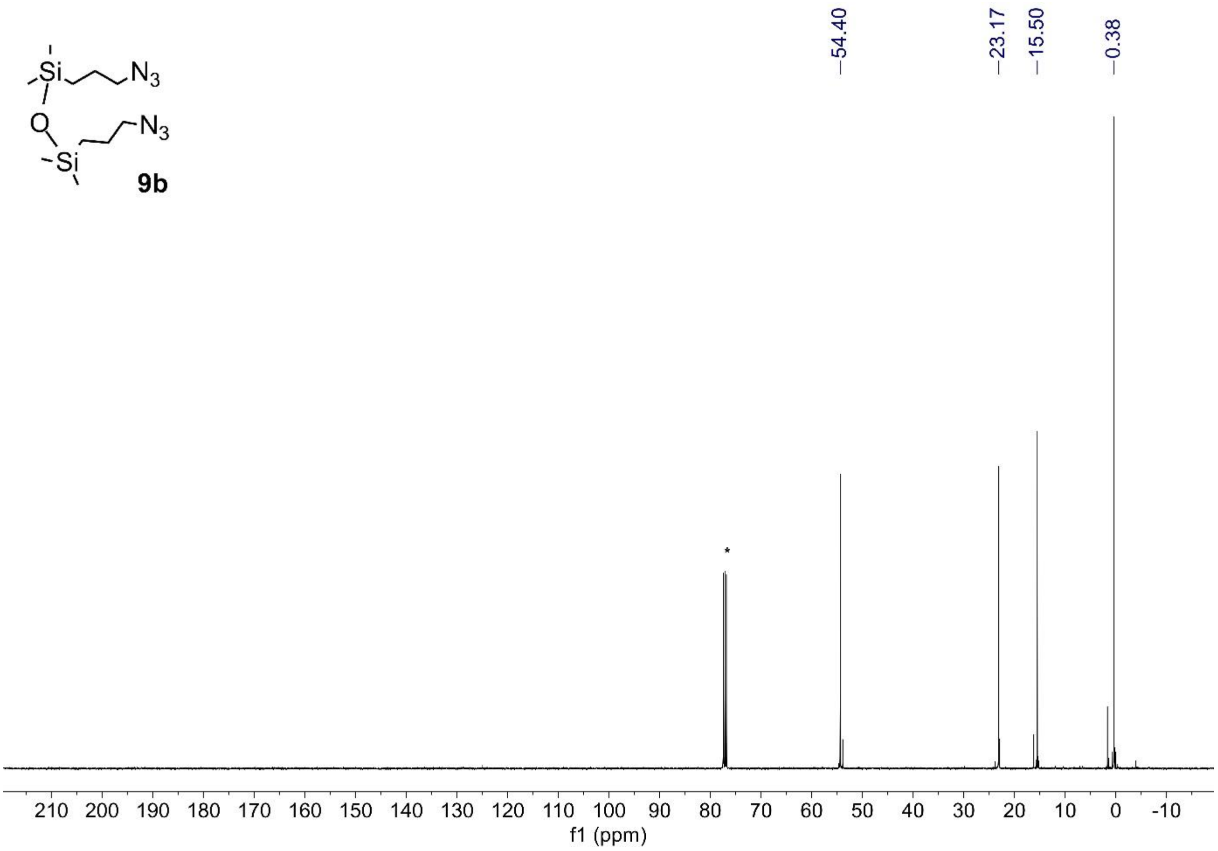

Figure S13. Compound **9b**, $^{29}Si\ \{^{1}H\}$ NMR (79 MHz, chloroform-*d*).

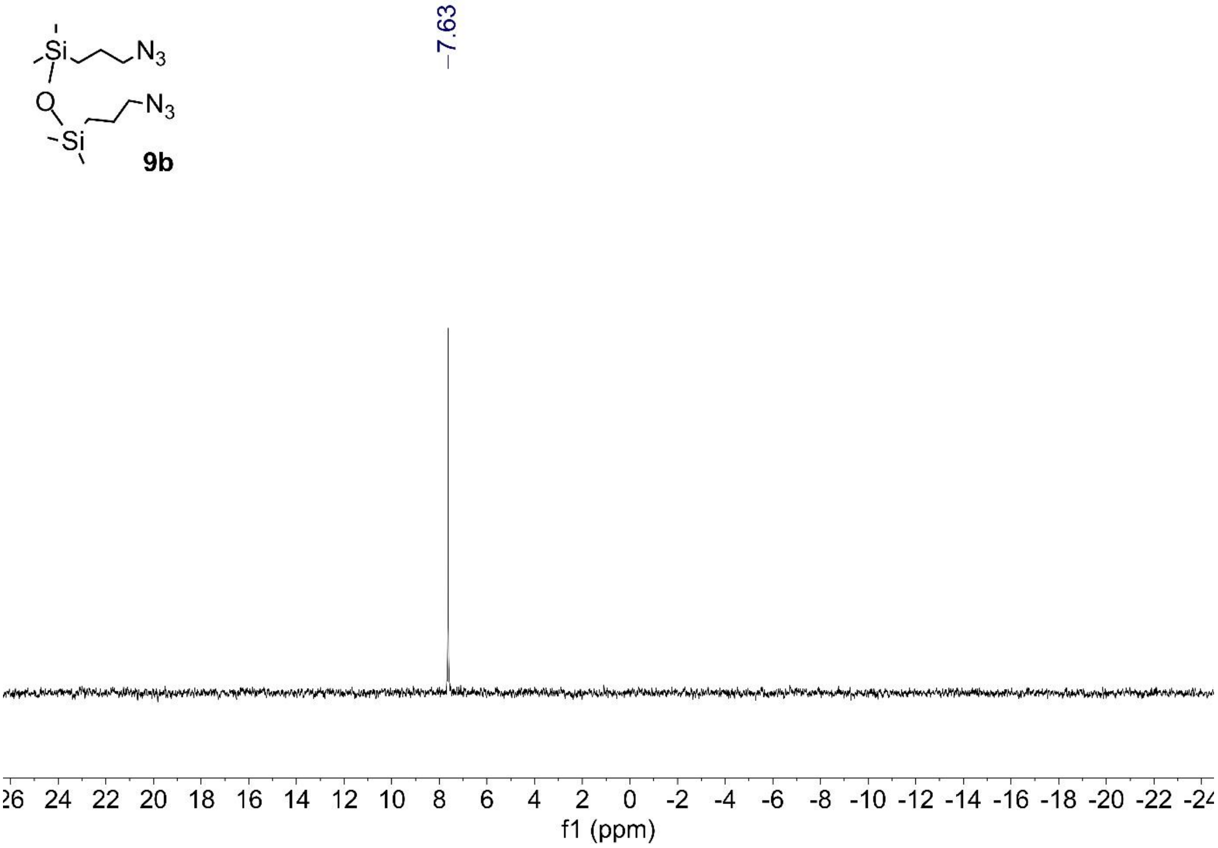

Figure S14. Compound **10**, ^{1}H NMR (400 MHz, DMSO-d_6).

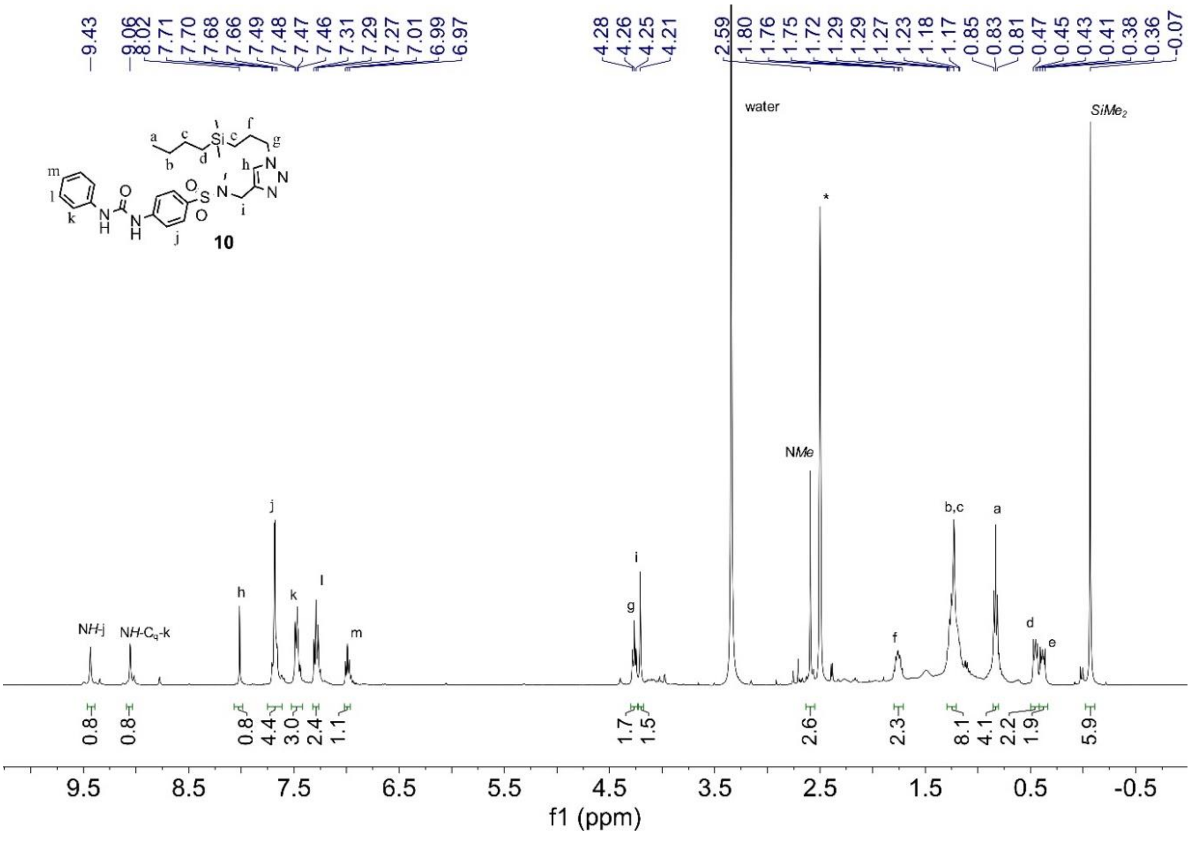

Figure S15. Compound **10**, $^{13}C\{^1H\}$ NMR (100 MHz, DMSO-d_6).

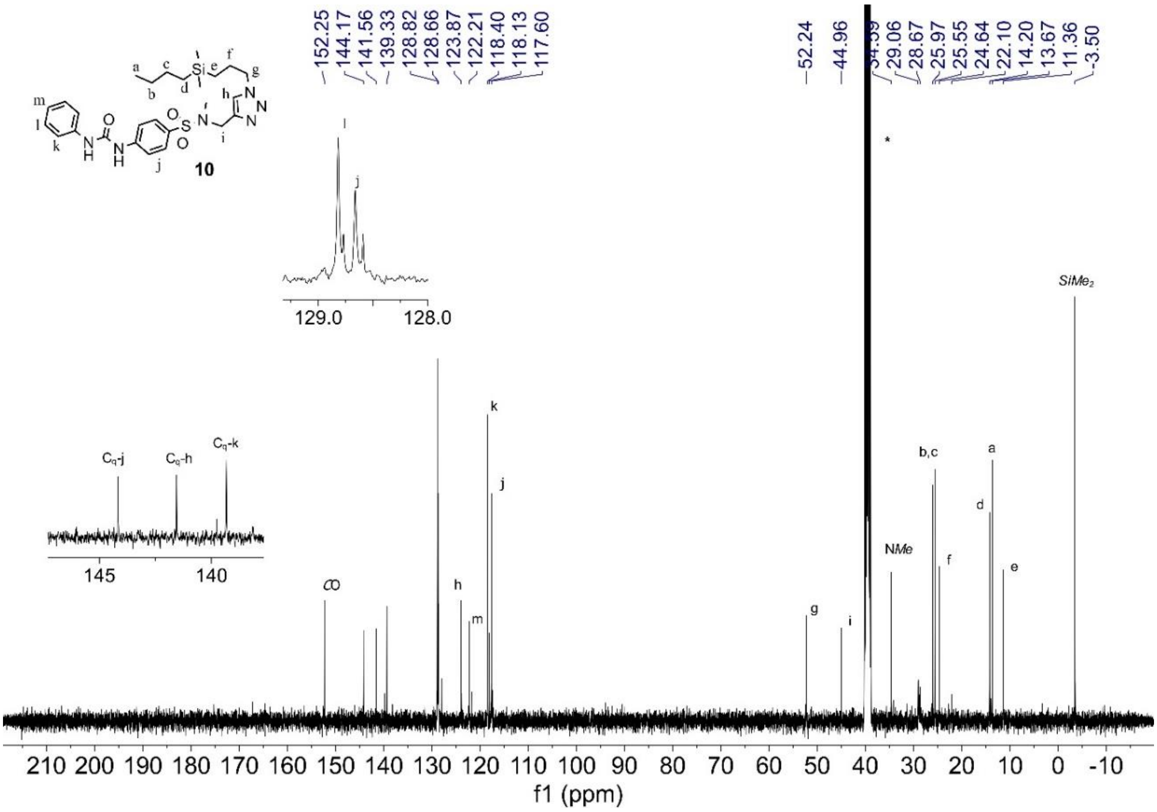

Figure S16. Compound **10**, $^{29}Si\{^1H\}$ NMR (79 MHz, DMSO-d_6).

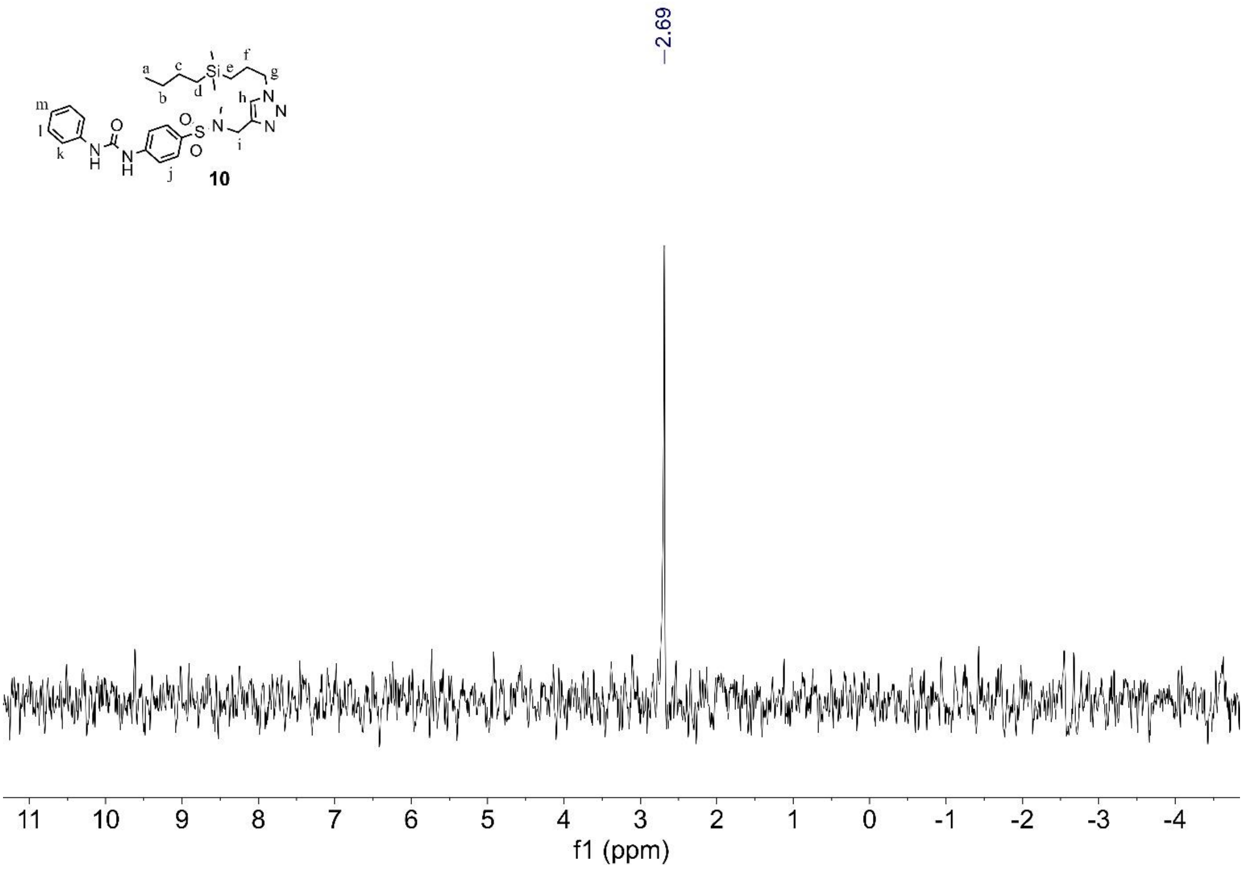

Figure S17. Compound **10**, ^{1}H-^{1}H COSY NMR, (400 MHz, DMSO-d_6).

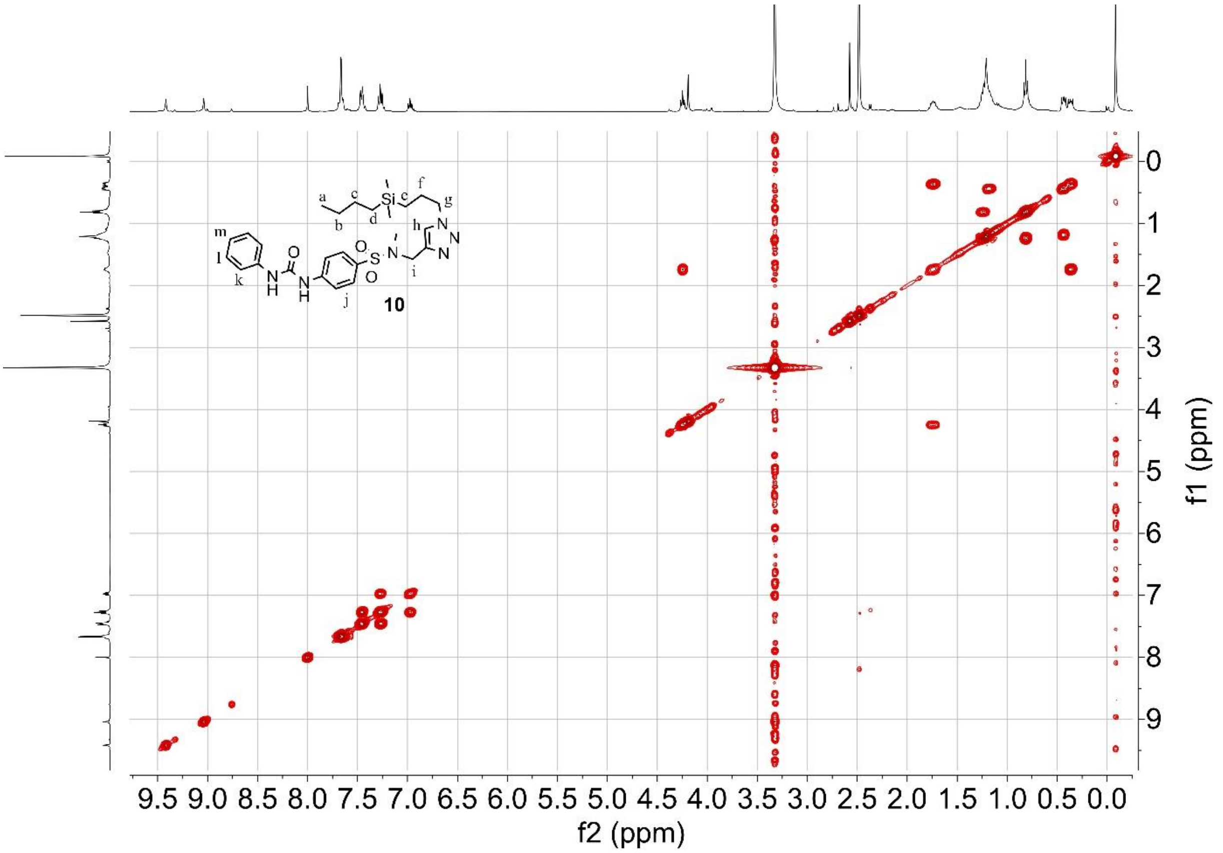

Figure S18. Compound **10**, ^{1}H-^{13}C HSQC NMR (400/100 MHz, DMSO-d_6).

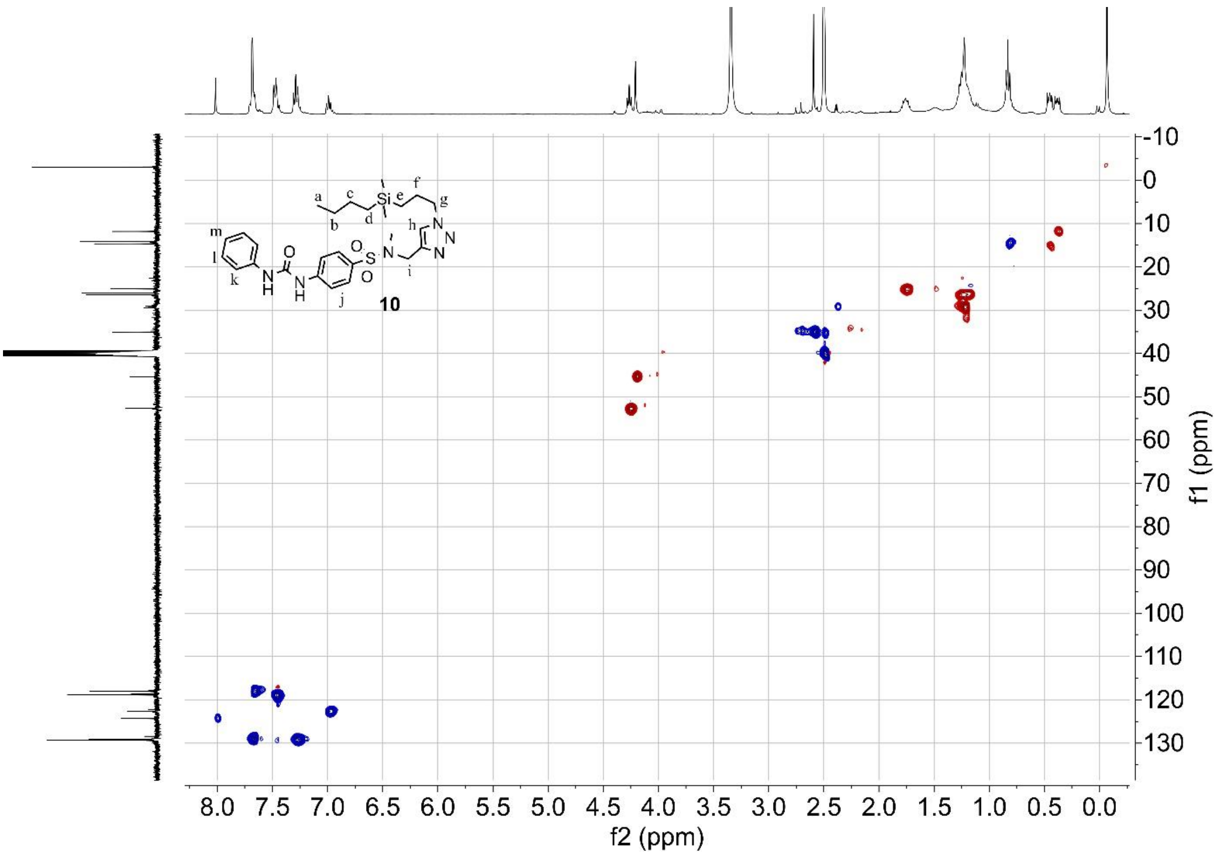

Figure S19. Compound **10**, ^{1}H-^{13}C HMBC NMR (400/100 MHz, DMSO-d_6).

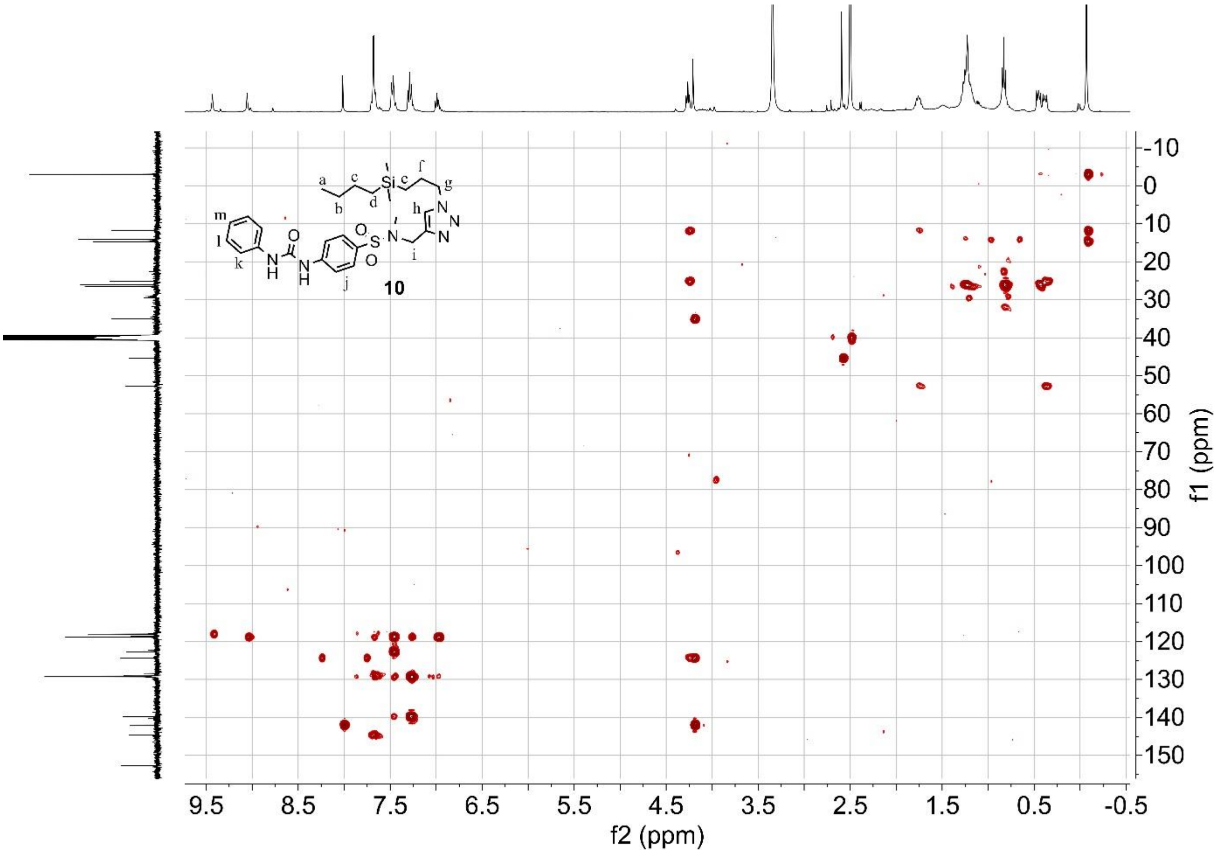

Figure S20. Compound **11**, ^{1}H NMR (400 MHz, DMSO-d_6).

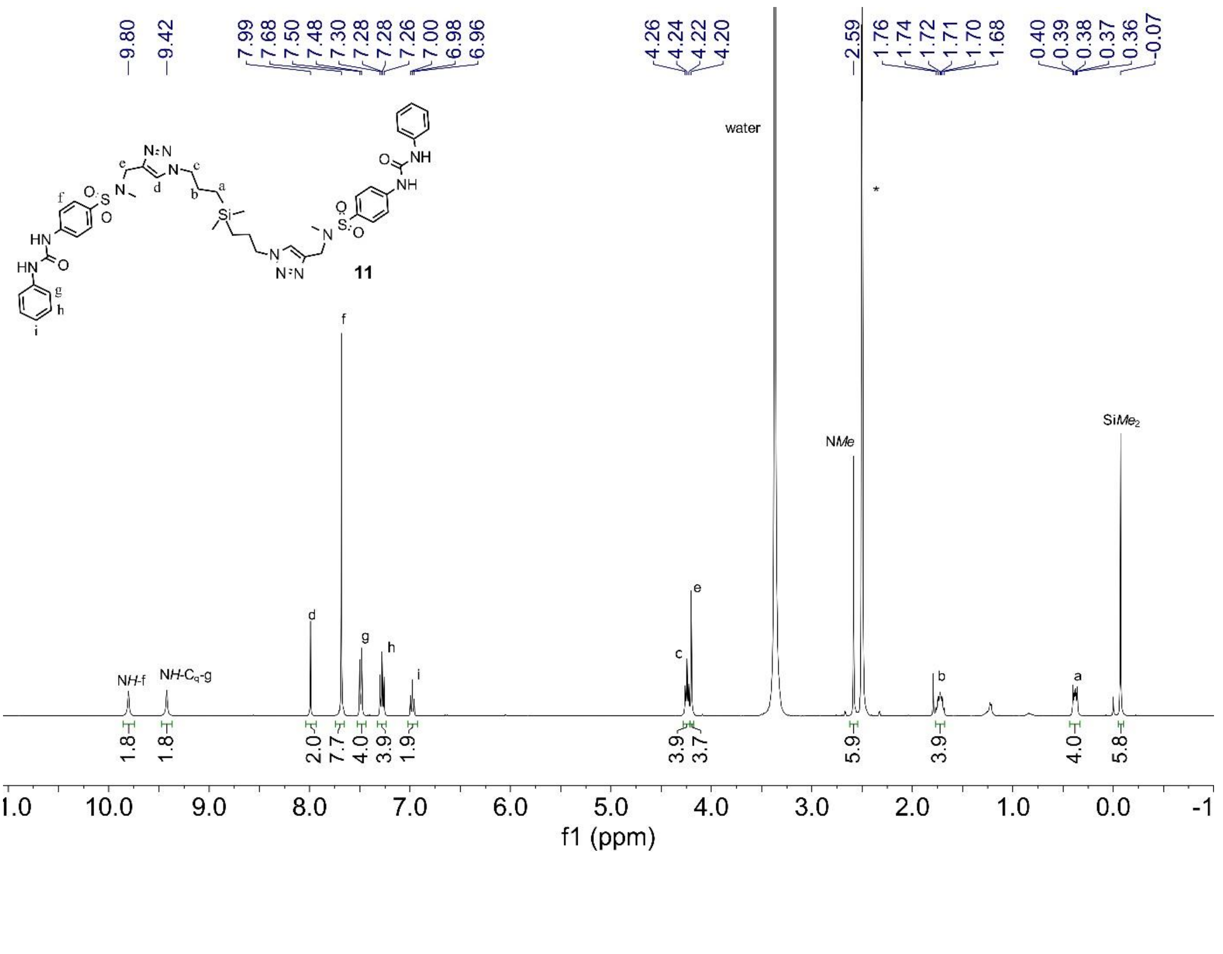

Figure S21. Compound **11**, ^{13}C {^{1}H} NMR (100 MHz, DMSO-d_6).

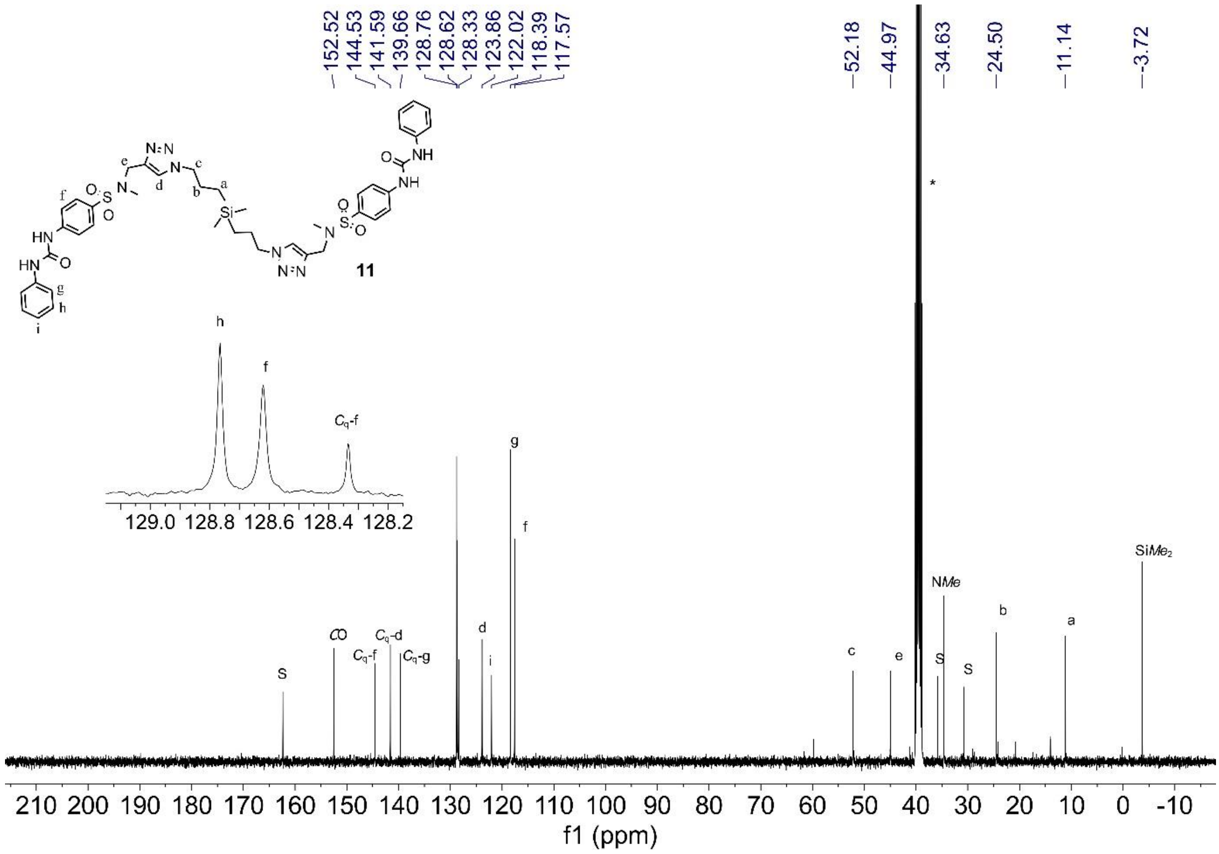

Figure S22. Compound **11**, ^{29}Si {^{1}H} NMR (79 MHz, DMSO-d_6).

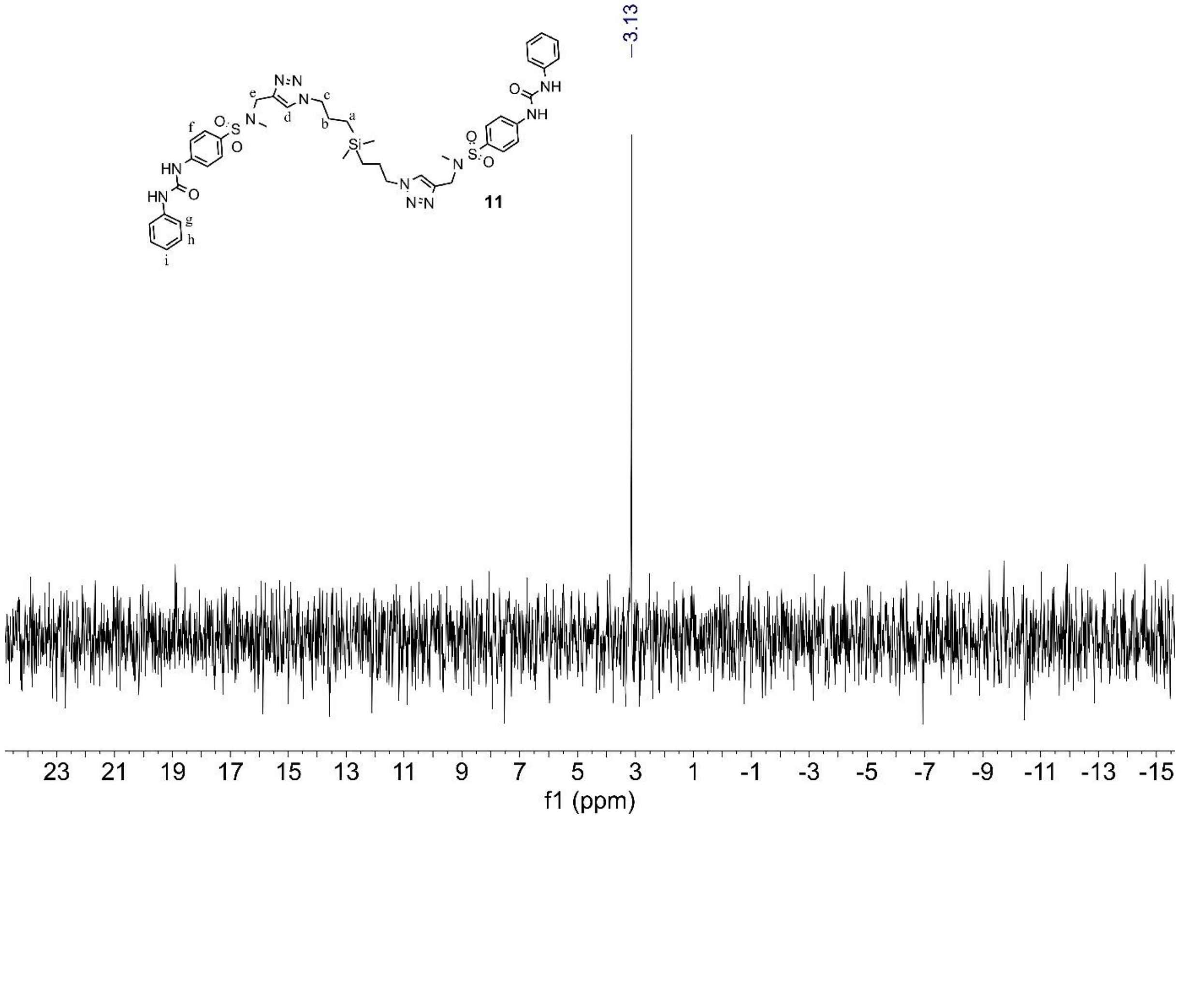

Figure S23. Compound **11**, ^{1}H-^{13}C HSQC NMR, (400/100 MHz, DMSO-d_6).

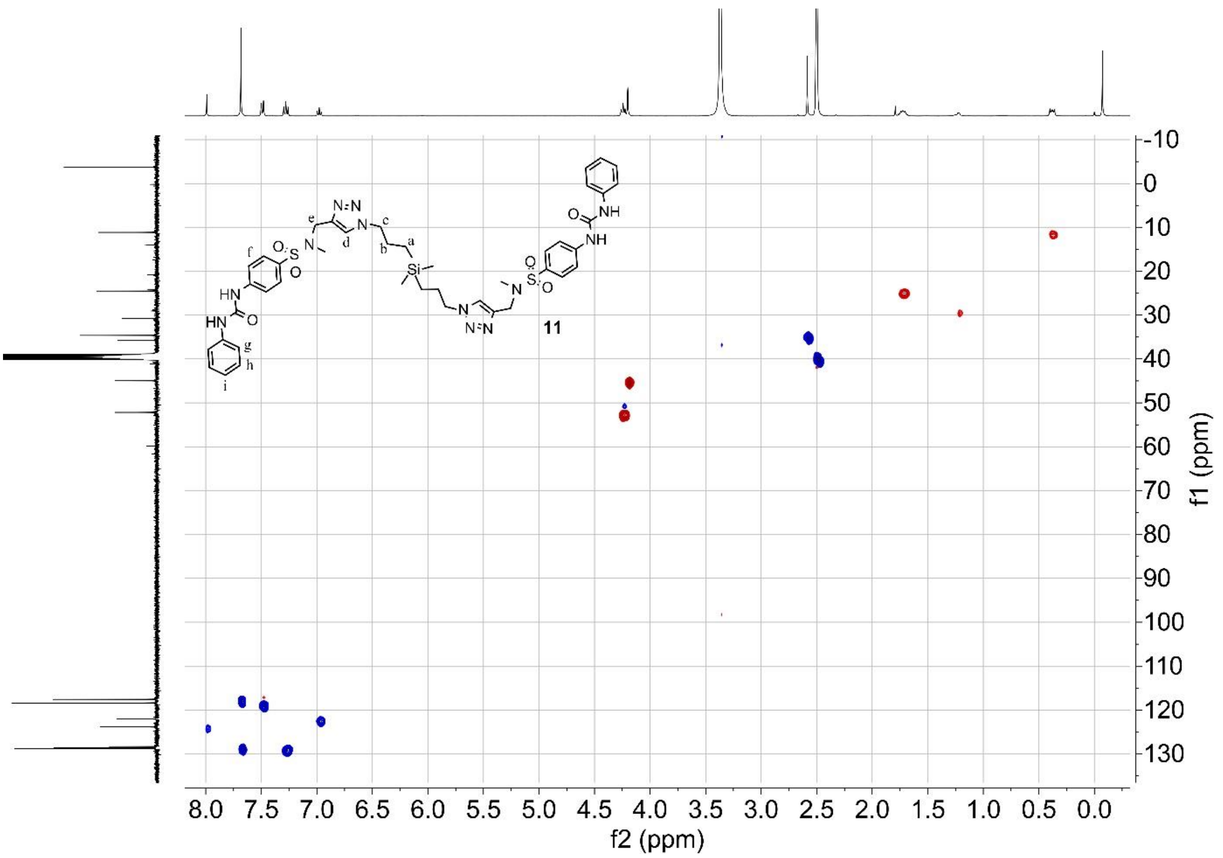

Figure S24. Compound **11**, ^{1}H-^{13}C HMBC NMR (400/100 MHz DMSO-d_6).

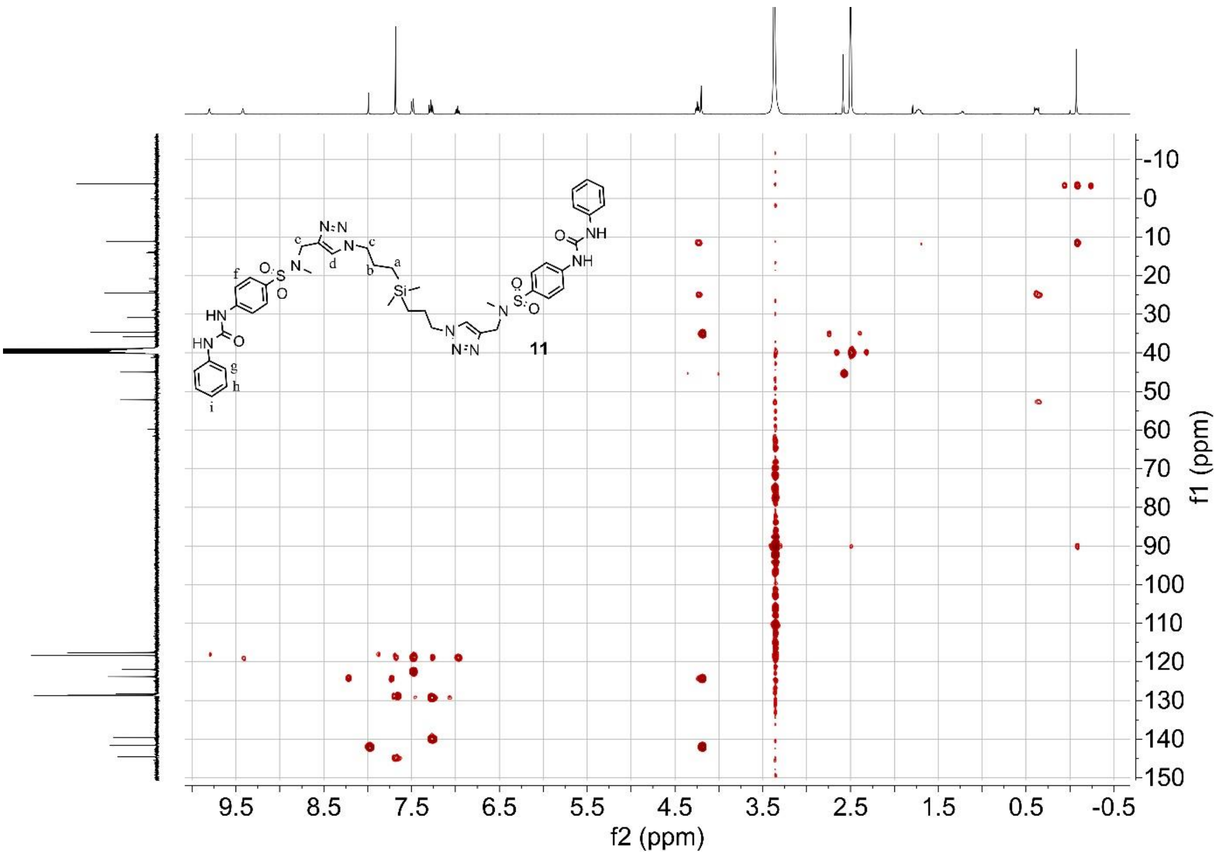

Figure S25. Compound **12**, ^{1}H NMR (400 MHz, DMSO-d_6).

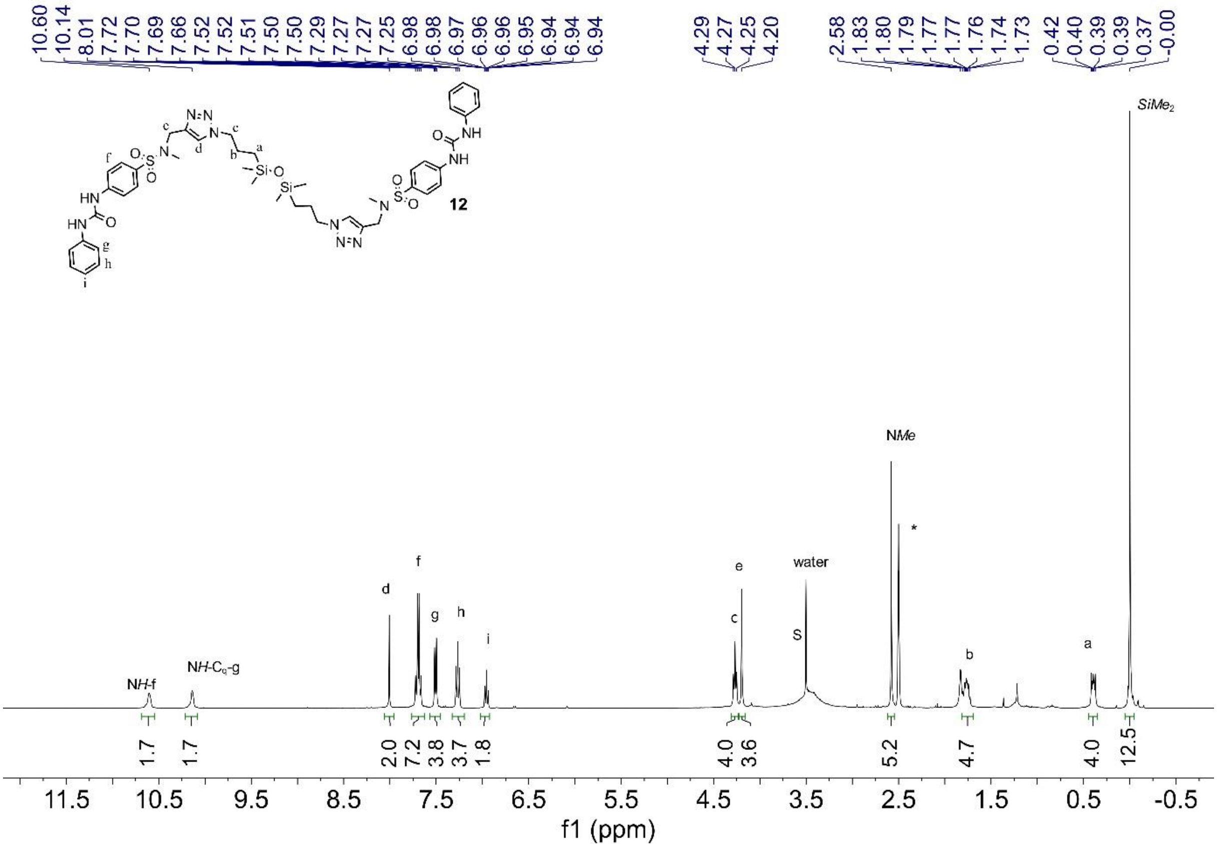

Figure S26. Compound **12**, ^{13}C {^{1}H} NMR (100 MHz, DMSO-d_6).

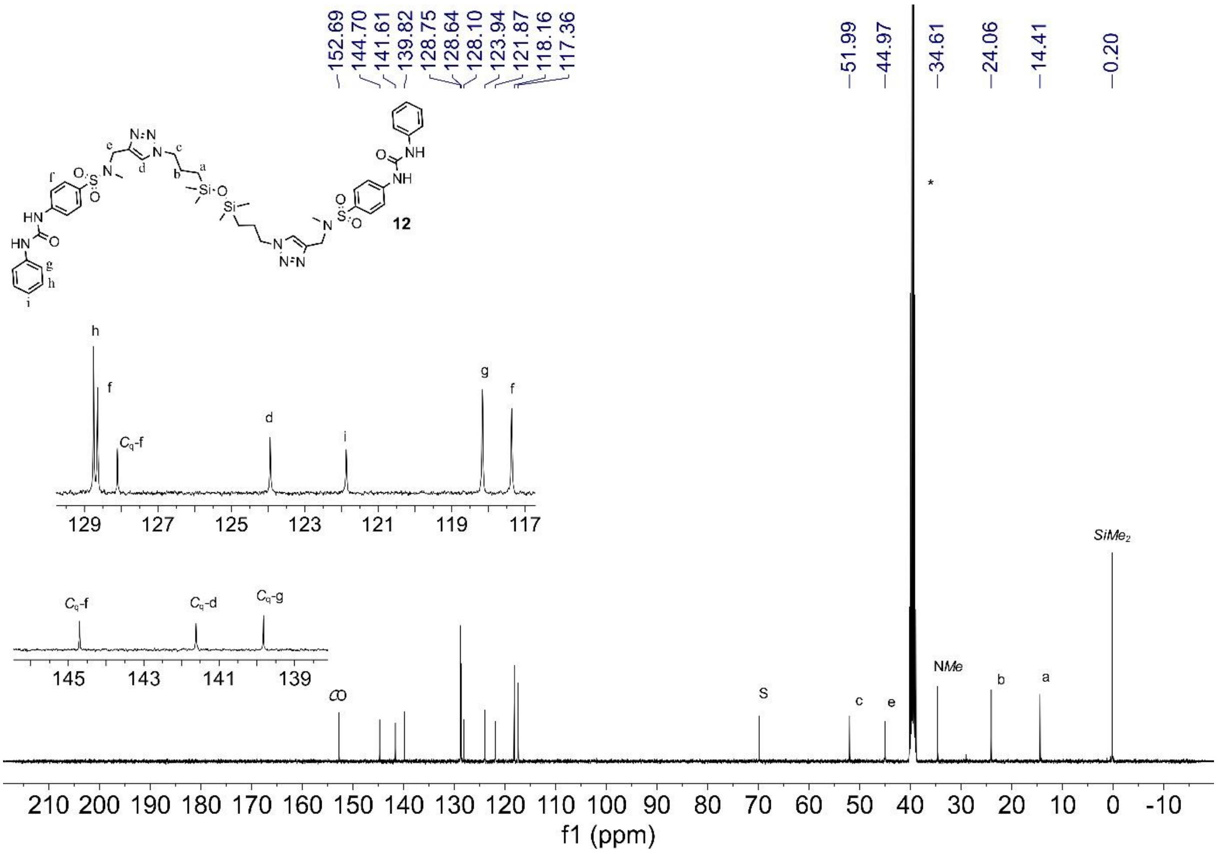

Figure S27. Compound **12**, ^{29}Si {^{1}H} NMR (79 MHz, DMSO-d_6).

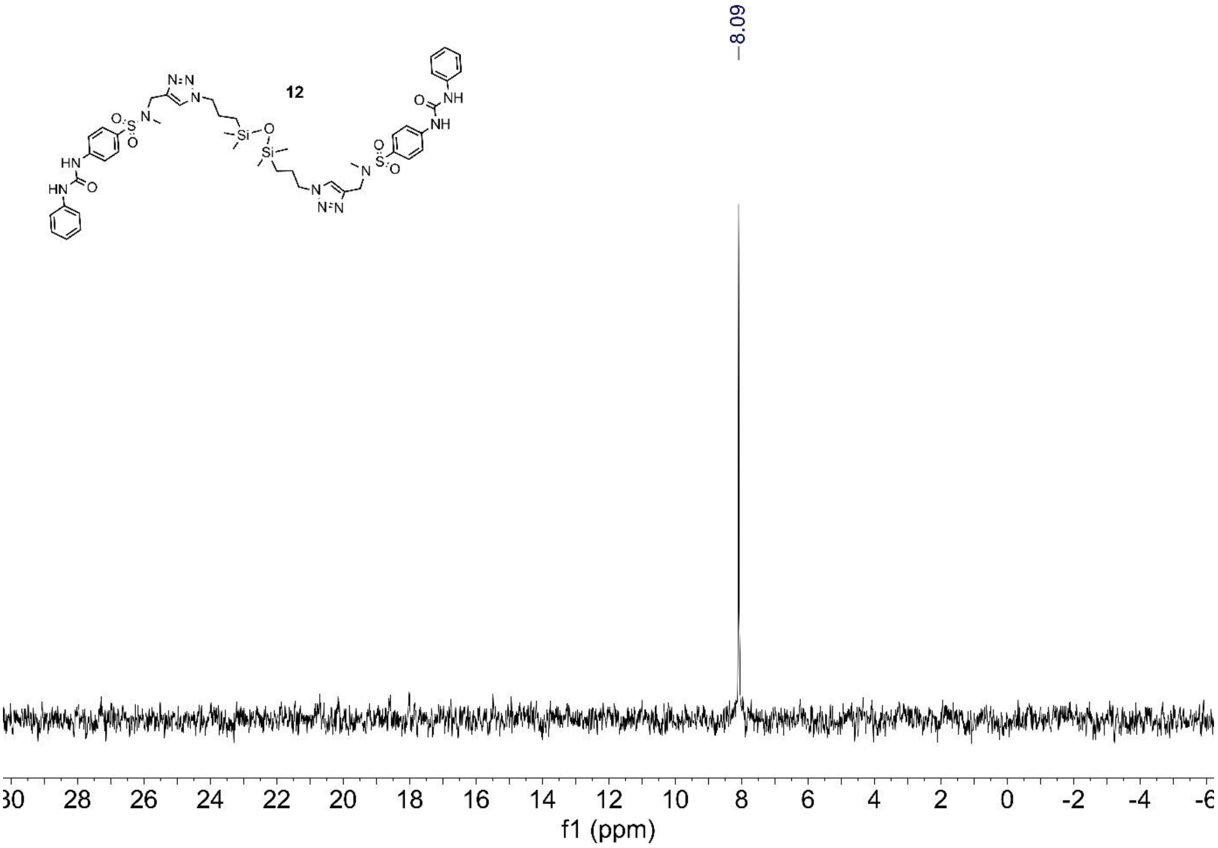

Figure S28. Compound **12**, ^{1}H-^{1}H COSY NMR (400 MHz, DMSO-d_6).

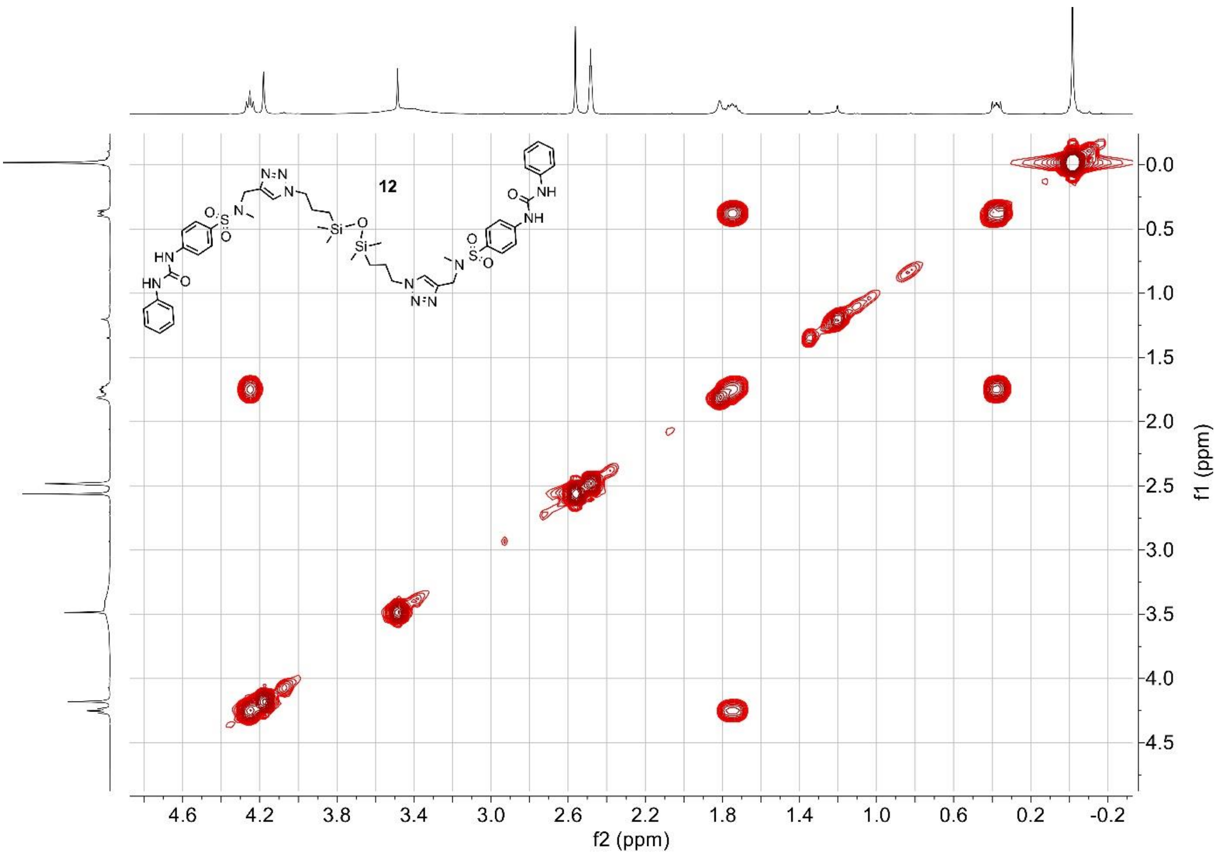

Figure S29. Compound **12**, ^{1}H-^{13}C HSQC NMR (400/100 MHz, DMSO-d_6).

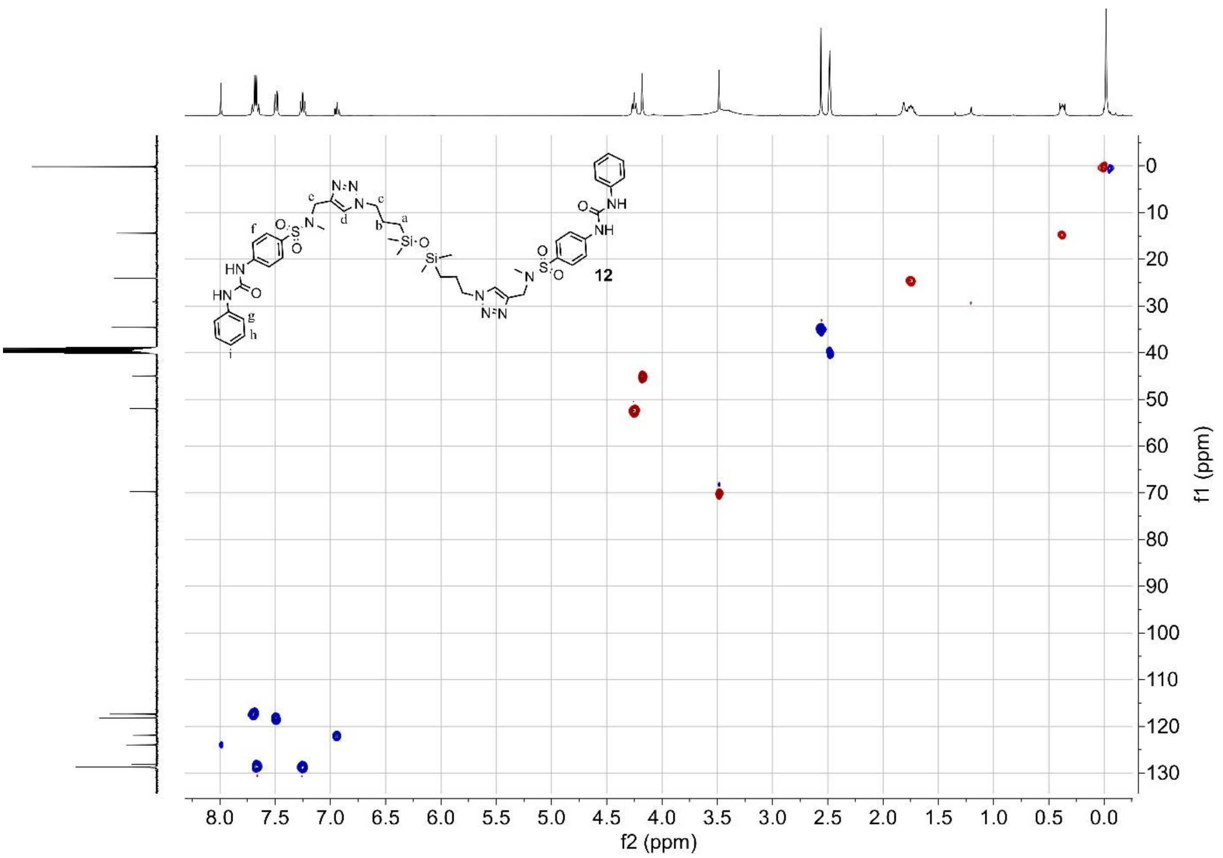

Figure S30. Compound **12**, ^{1}H-^{13}C HMBC NMR, (400/100 MHz, DMSO-d_6).

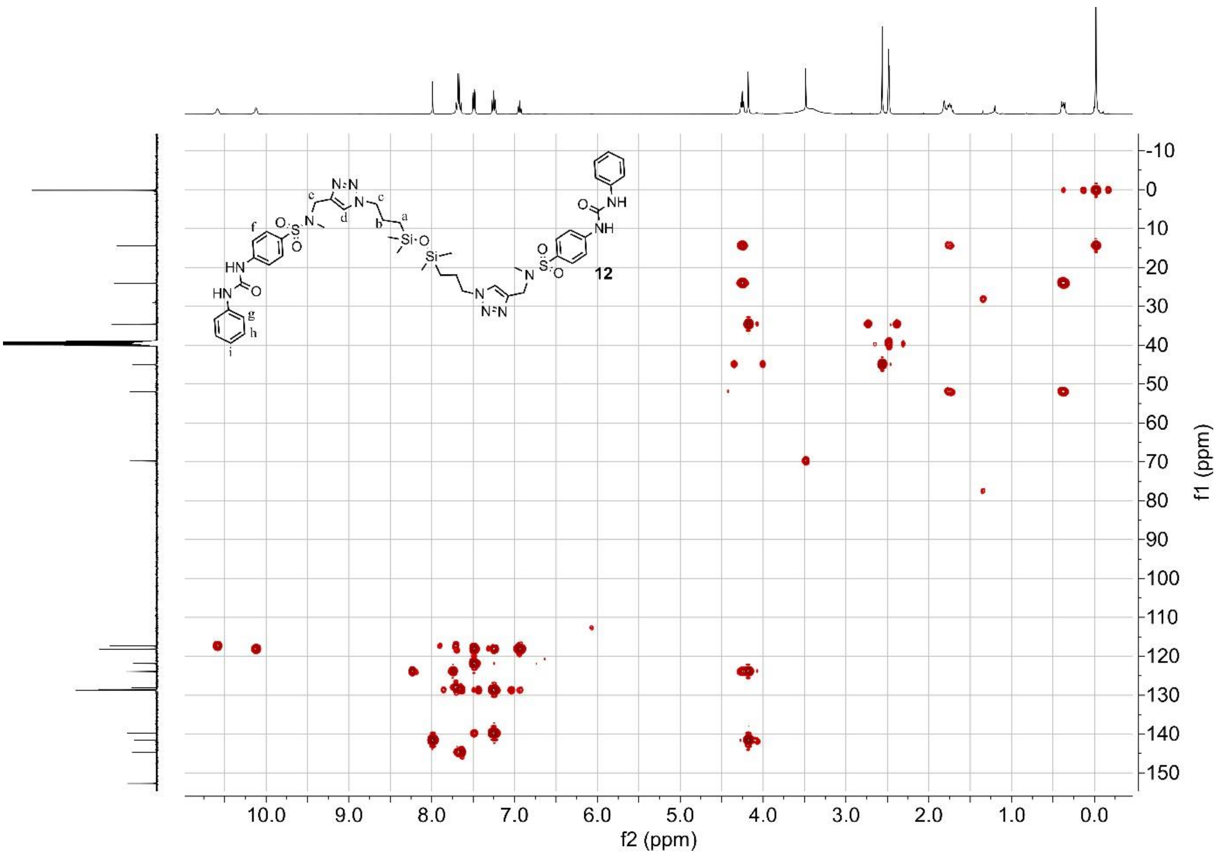

Figure S31. Compound **13**, ^{1}H NMR (400 MHz, DMSO-d_6).

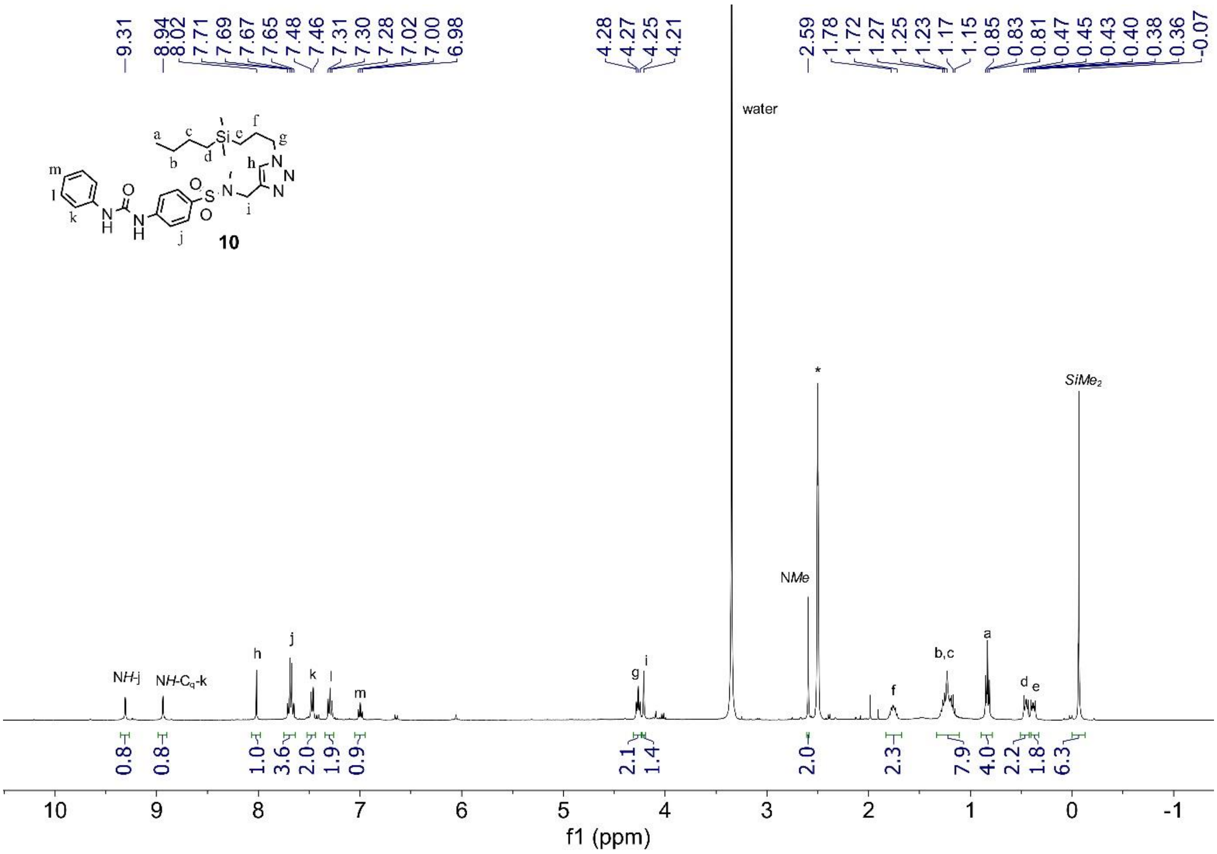

Figure S32. Compound **13**, ^{13}C {^{1}H} NMR (100 MHz, DMSO-d_6).

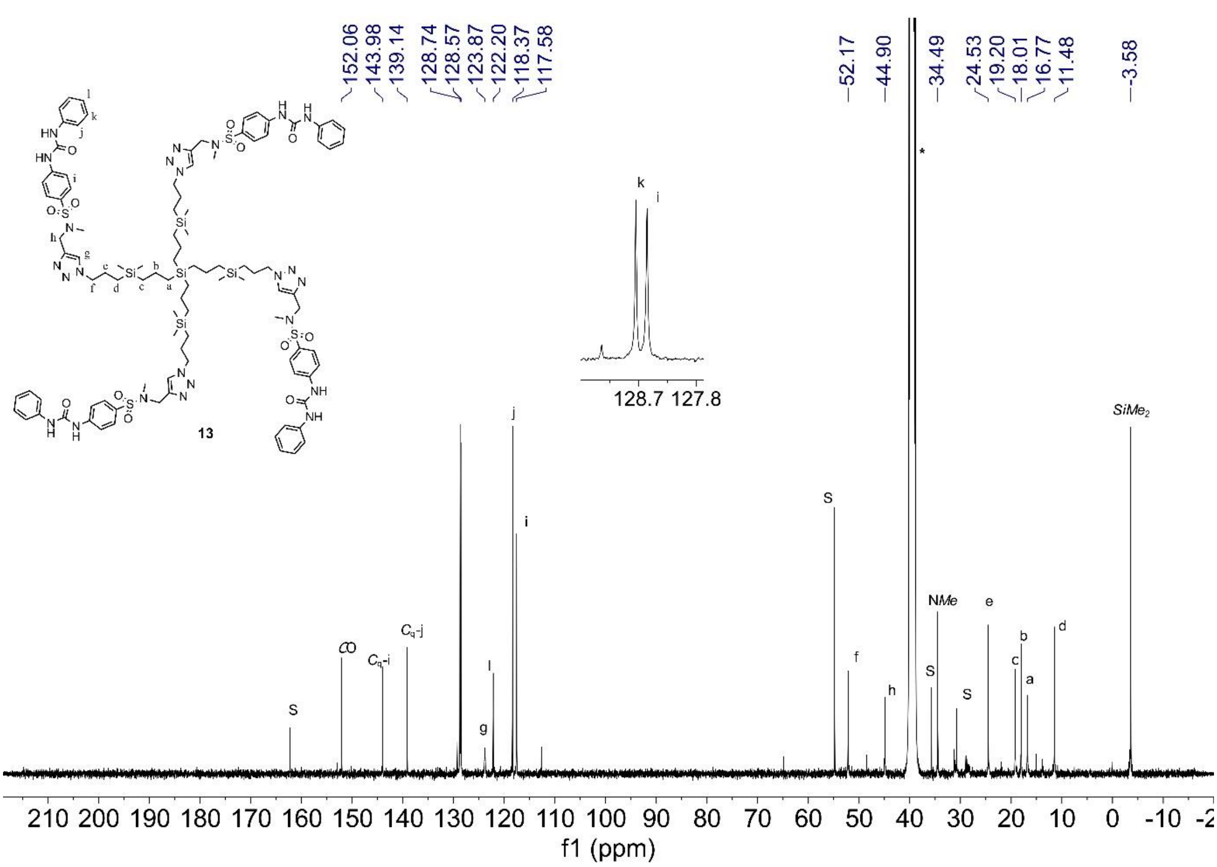

Figure S33. Compound **13**, $^{29}Si\ \{^{1}H\}$ NMR (79 MHz, DMSO-d_6).

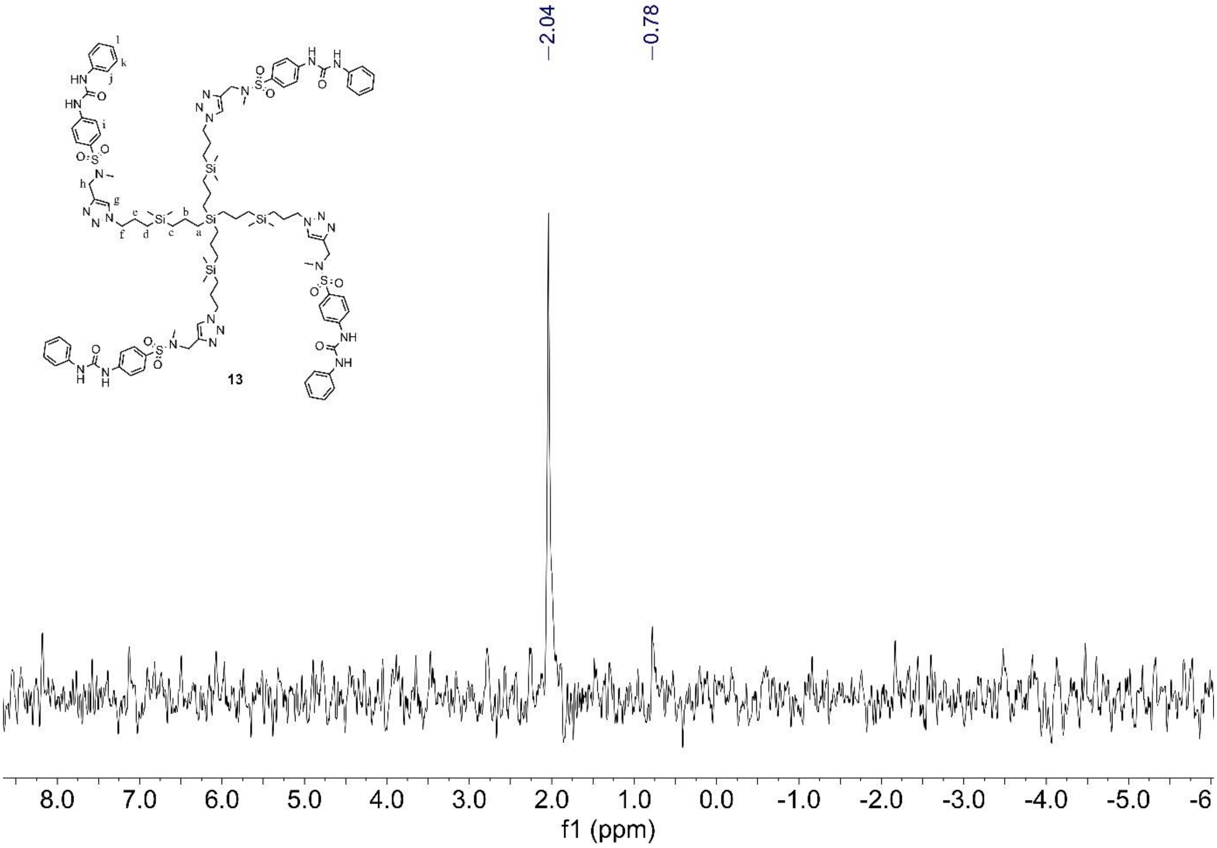

Figure S34. Compound **13**, ^{1}H-^{1}H COSY NMR, (400 MHz, DMSO-d_6).

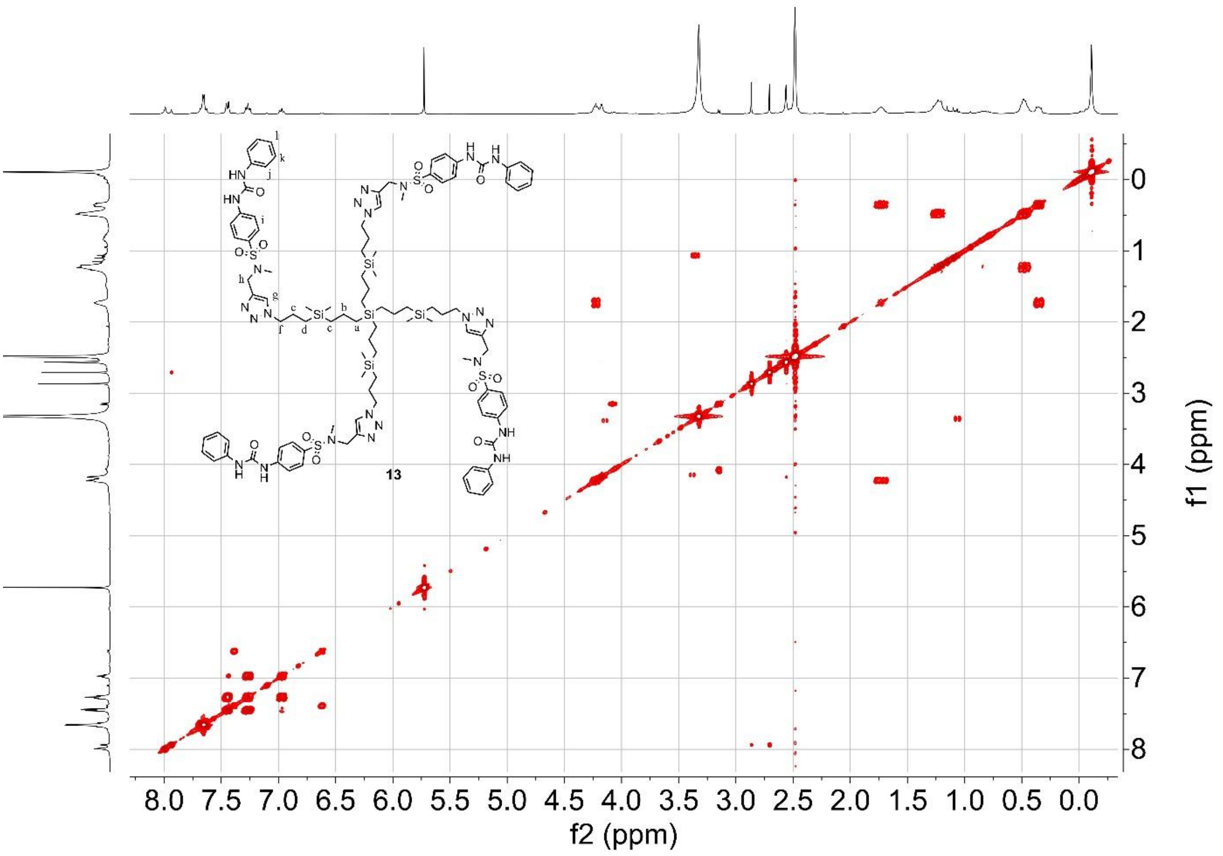

Figure S35. Compound **13**, ^{1}H-^{13}C HSQC NMR (400/100 MHz, DMSO-d_6).

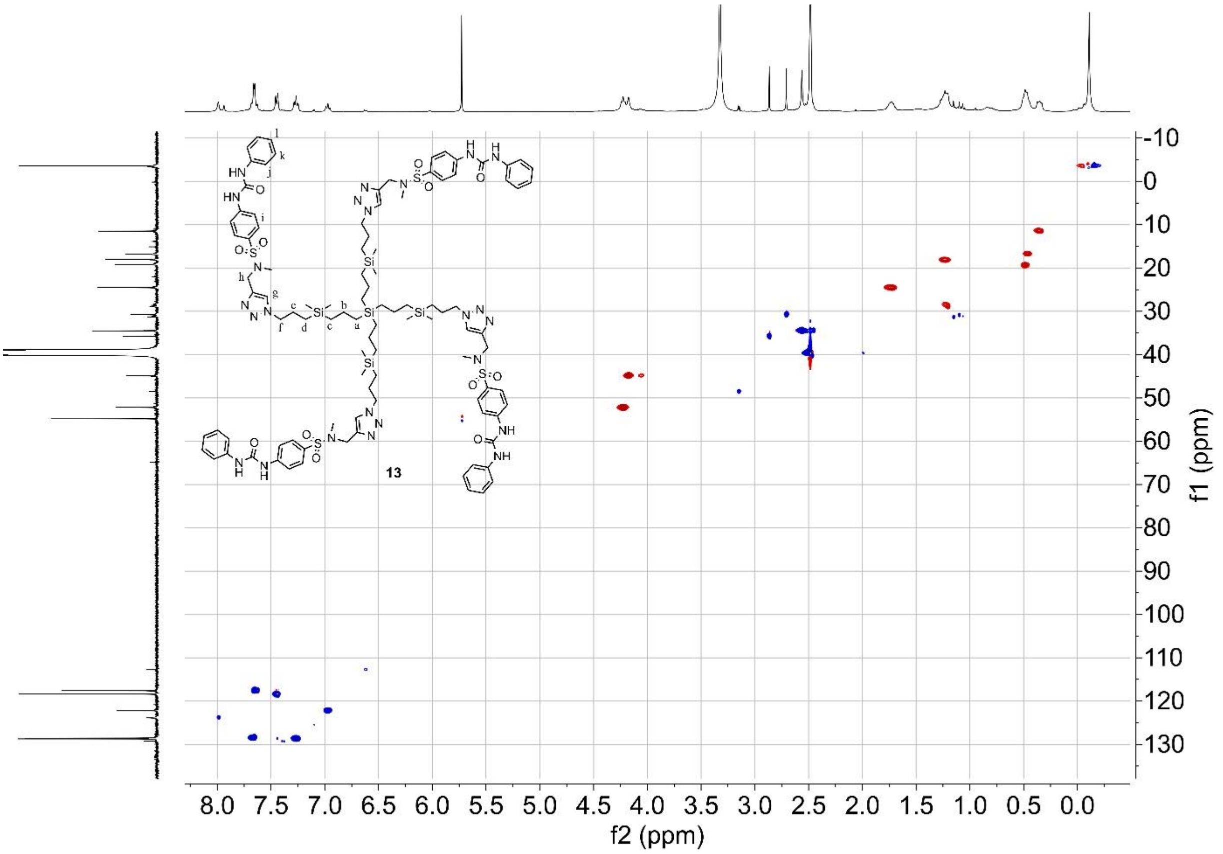

Figure S36. Compound **13**, ^{1}H-^{13}C HMBC NMR (400/100 MHz, DMSO-d_6).

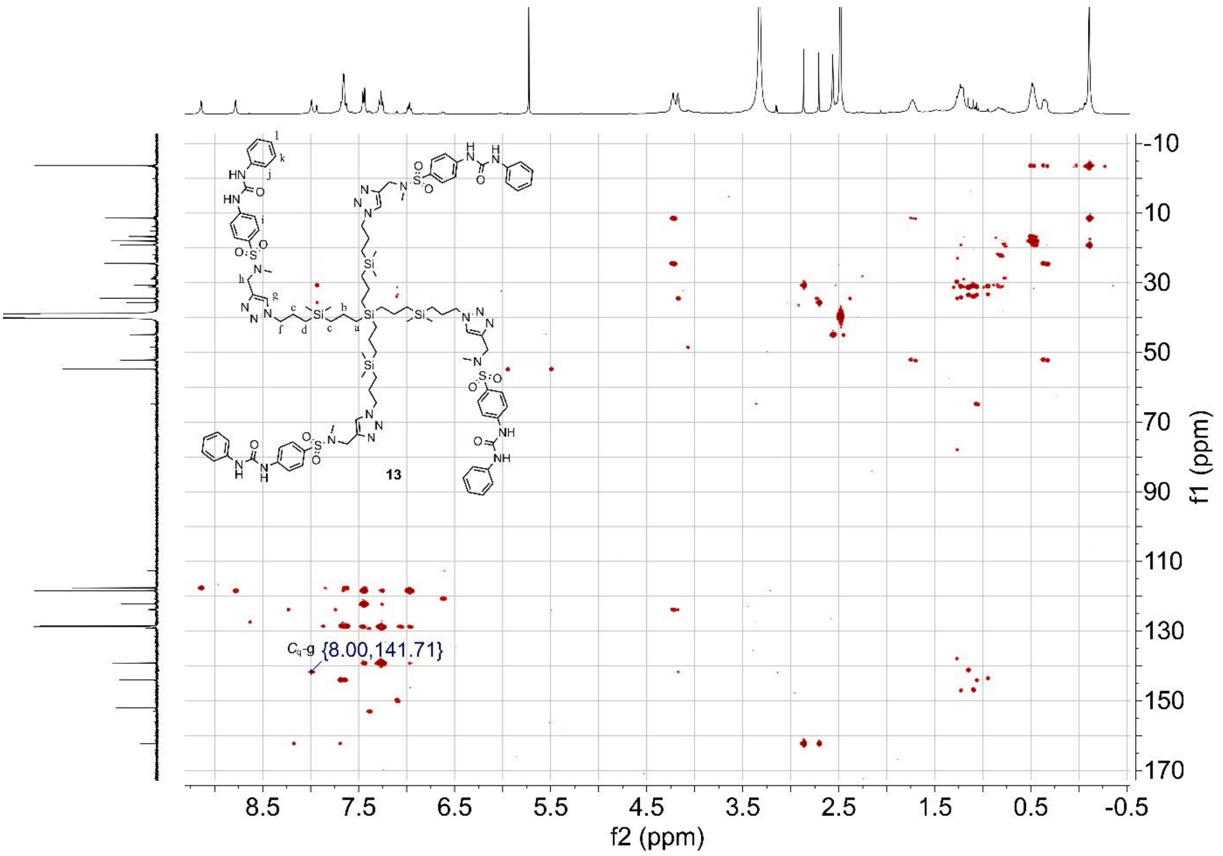

Figure S37. Compound **14**, ^{1}H NMR (400 MHz, DMSO-d_6).

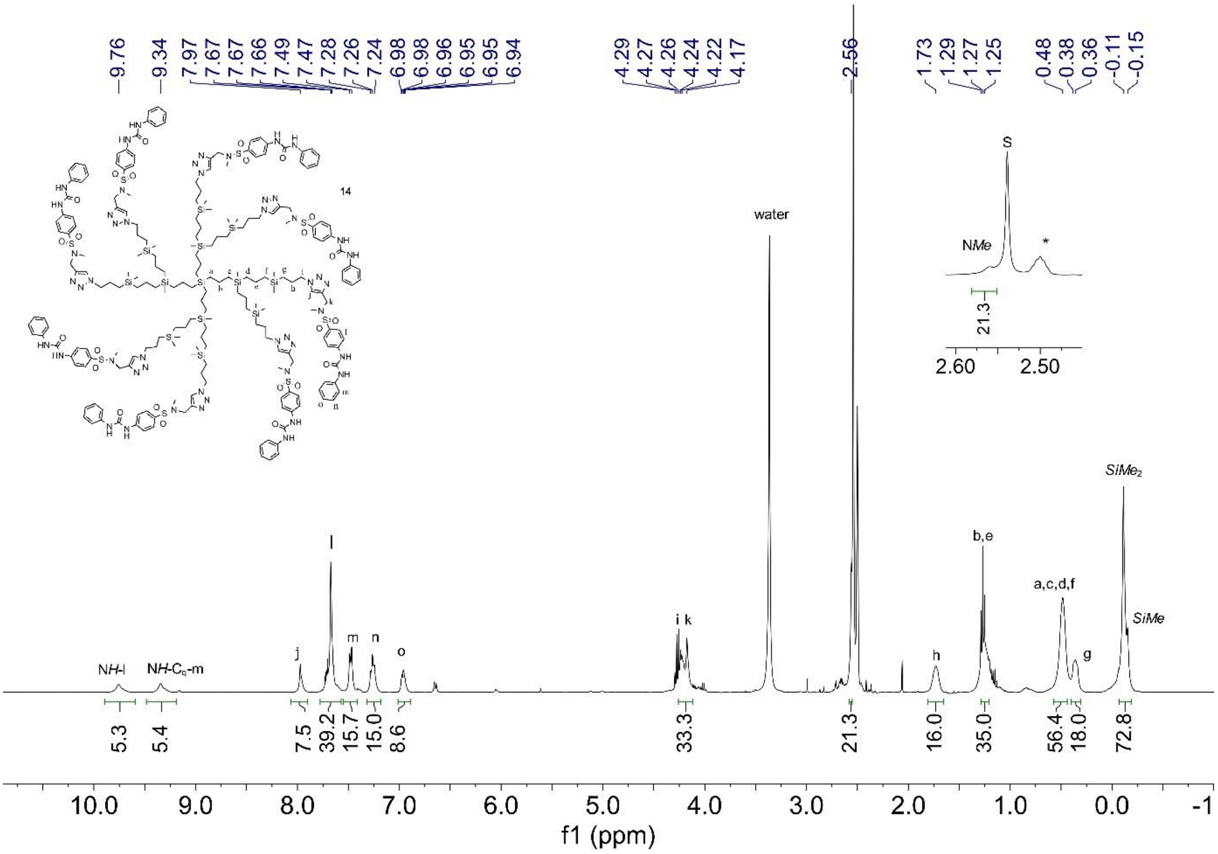

Figure S38. Compound **14**, ^{13}C {^{1}H} NMR (100 MHz, DMSO-d_6).

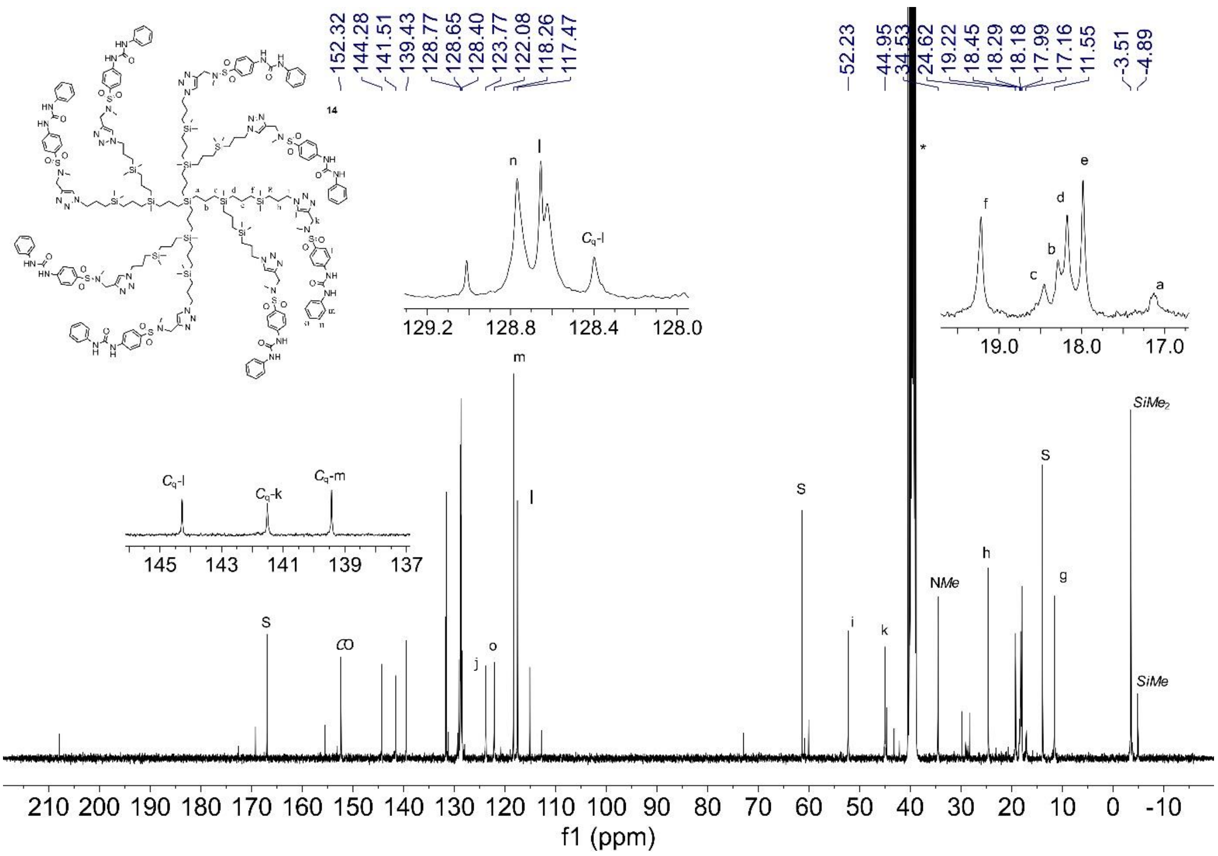

Figure S39. Compound **14**, ^{29}Si {^{1}H} NMR (79 MHz, DMSO-d_6).

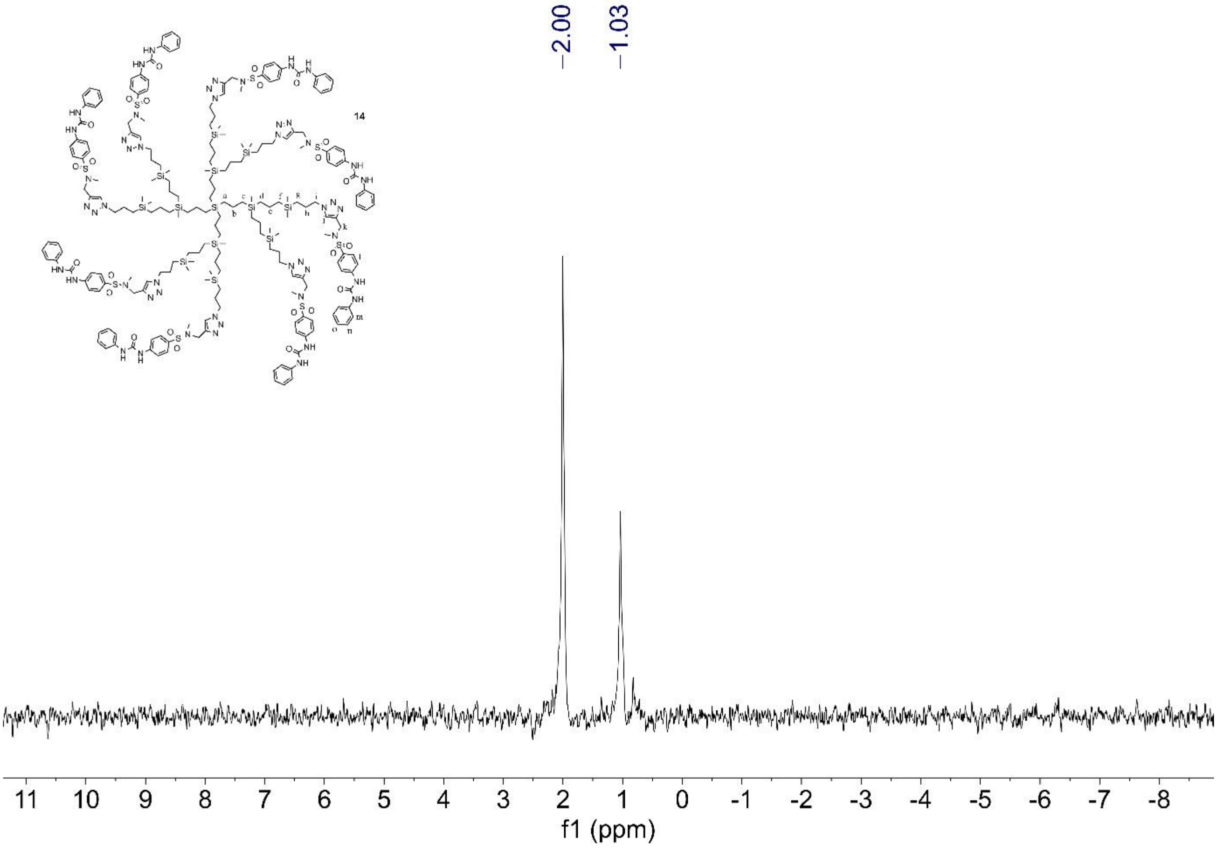

Figure S40. Compound **14**, ^{1}H-^{1}H COSY NMR, (400 MHz, DMSO-d_6).

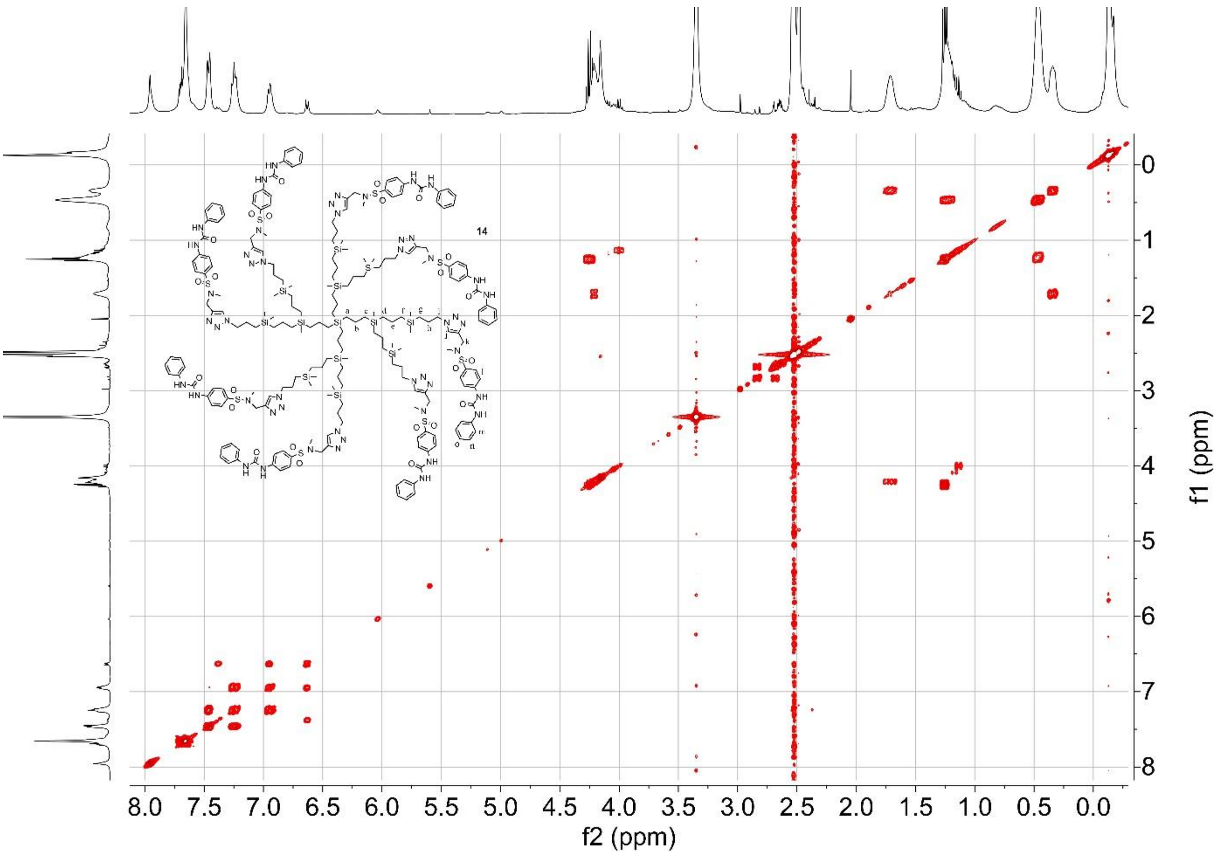

Figure S41. Compound **14**, ^{1}H-^{13}C HSQC NMR (400/100 MHz, DMSO-d_6).

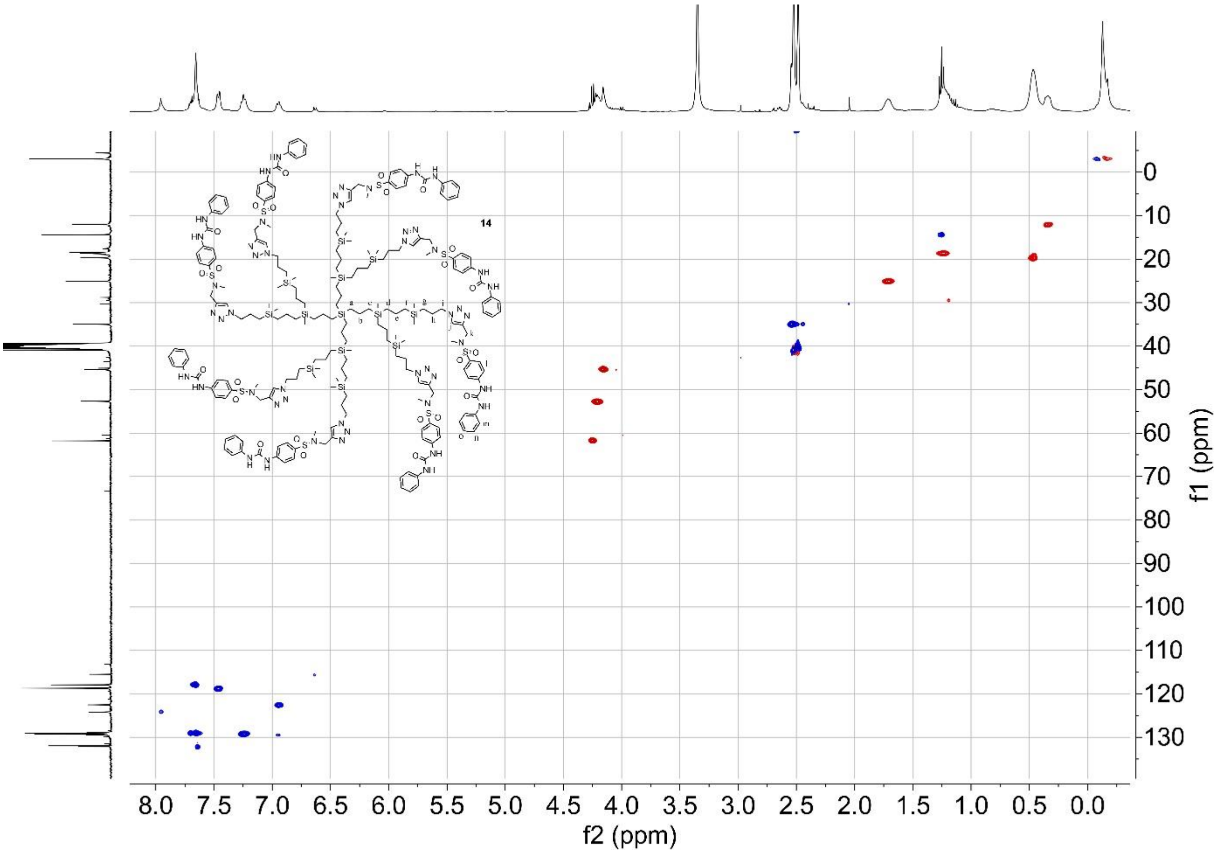

Figure S42. Compound **14**, ^{1}H-^{13}C HMBC NMR, (400/100 MHz, DMSO-d_6).

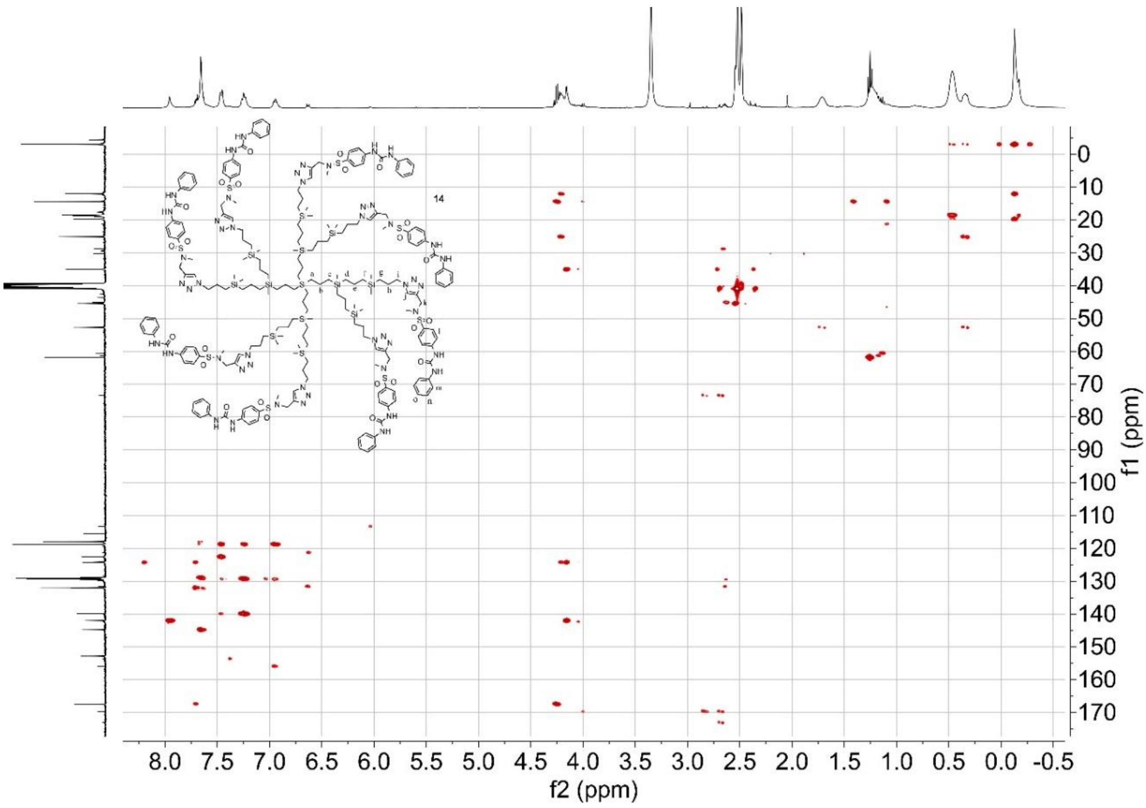

UV and ^{1}H NMR titration data with links to on-line saved fits.

c 10, [mol/L]	c anion [mol/L]	A λ = 300 nm [AU]
0.0004	0	0.220
0.0004	0.0001	0.316
0.0004	0.0003	0.401
0.0004	0.0004	0.456
0.0004	0.0006	0.531
0.0004	0.0008	0.586
0.0004	0.0011	0.630
0.0004	0.0015	0.681
0.0004	0.0018	0.719
0.0004	0.0024	0.748
0.0004	0.0028	0.771
0.0004	0.0032	0.782

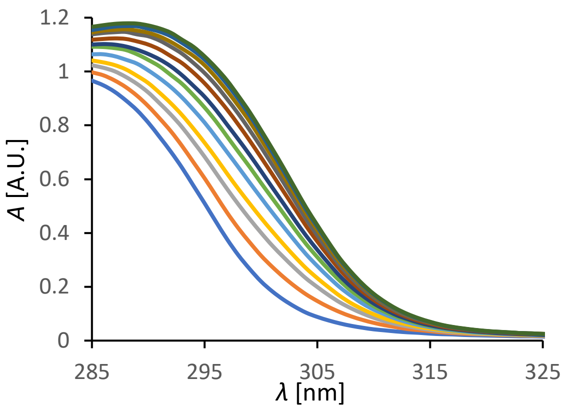

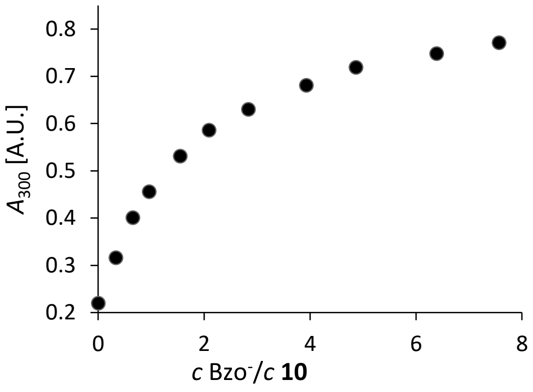

http://app.supramolecular.org/bindfit/view/3afbb4ea-5219-4686-b379-62f8c79ffa40

Figure S43. Receptor **10**, UV-Vis titration by $TBA^{+}BzO^{-}$ in DMSO.

c 10, [mol/L]	c anion [mol/L]	A λ = 300 nm [AU]
0.0003	0	0.149
0.0003	0.0001	0.183
0.0003	0.0002	0.220
0.0003	0.0003	0.249
0.0003	0.0004	0.294
0.0003	0.0006	0.328
0.0003	0.0008	0.360
0.0003	0.0011	0.393
0.0003	0.0013	0.416
0.0003	0.0017	0.443
0.0003	0.0020	0.441
0.0003	0.0023	0.458

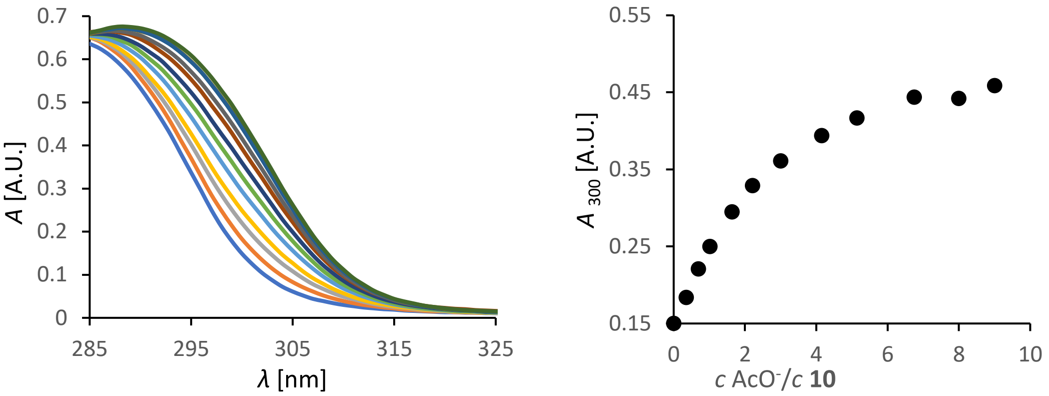

http://app.supramolecular.org/bindfit/view/7a8d506c-48d4-4f42-a0bb-c4761ed51715

Figure S44. Receptor **10**, UV-Vis titration by TBA^+AcO^- in DMSO.

**c 10, [mol/L]**	**c anion [mol/L]**	**A *λ* = 300 nm [AU]**
0.0004	0	0.222
0.0004	0.0001	0.357
0.0004	0.0003	0.448
0.0004	0.0004	0.505
0.0004	0.0006	0.567
0.0004	0.0008	0.596
0.0004	0.0011	0.638
0.0004	0.0015	0.674
0.0004	0.0019	0.685
0.0004	0.0025	0.705
0.0004	0.0029	0.715
0.0004	0.0033	0.717

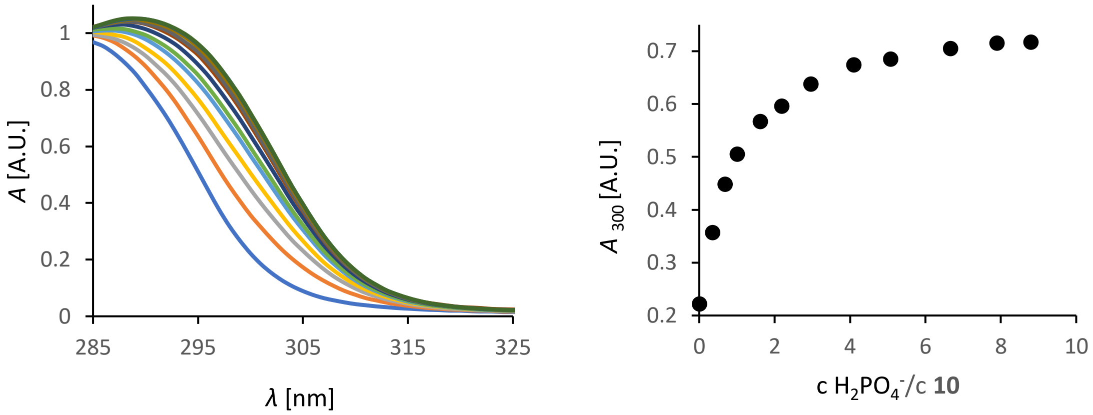

http://app.supramolecular.org/bindfit/view/160e65a4-6e1f-4356-8535-fabcca234f52

Figure S45. Receptor **10**, UV-Vis titration by TBA^+ $H_2PO_4^-$ in DMSO.

c 10, [mol/L]	c anion [mol/L]	Δδ ArH [Hz]
0.0042	0	0
0.0042	0.0005	9.0
0.0042	0.0010	19.2
0.0042	0.0014	30.1
0.0042	0.0023	43.4
0.0042	0.0031	56.2
0.0042	0.0043	65.8
0.0042	0.0059	74.9
0.0042	0.0073	79.0
0.0042	0.0096	79.6
0.0042	0.0114	80.9
0.0042	0.0256	80.9

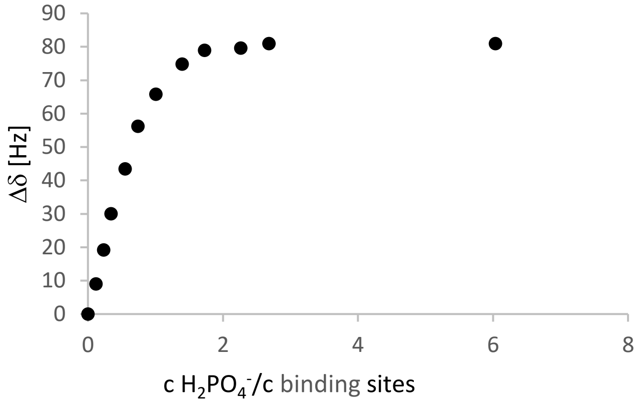

http://app.supramolecular.org/bindfit/view/95a8ac42-cf0e-487e-8785-48bec7d46cdd

Figure S46. Receptor **10,** ^{1}H NMR titration by $TBA^+H_2PO_4^-$ in DMSO-d_6, Ar*H*.

c 10, [mol/L]	c anion [mol/L]	Δδ ArH [Hz]
0.004545	0	0
0.004545	0.000552	9.45
0.004545	0.001084	18.3
0.004545	0.001595	26.1
0.004545	0.002561	39.9
0.004545	0.00346	52.3
0.004545	0.004695	61.1
0.004545	0.006501	73.1
0.004545	0.008049	77.0
0.004545	0.010565	77.9
0.004545	0.012521	78.2
0.004545	0.028173	84.8

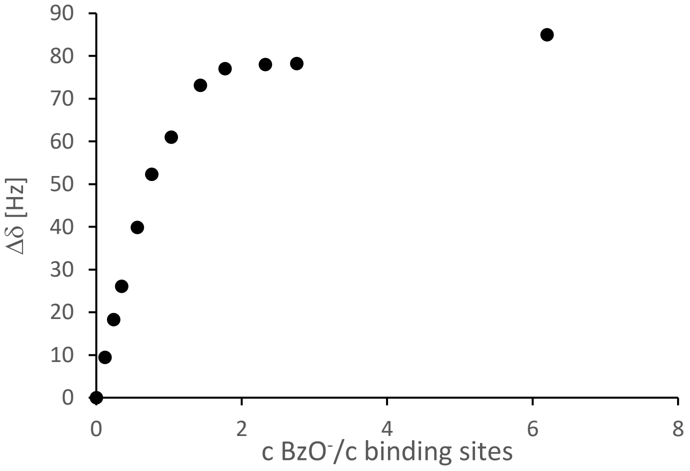

http://app.supramolecular.org/bindfit/view/b743ab76-5f6e-4aa9-9852-4c9d3ffa4d8d

Figure S47. Receptor **10,** ^{1}H NMR titration by TBA^+BzO^- in DMSO-d_6, Ar*H*

c 10, [mol/L]	c anion [mol/L]	Δδ NH [Hz]
0.003483	0	0
0.003483	0.000522	9.0
0.003483	0.001024	17.1
0.003483	0.001507	27.9
0.003483	0.00242	40.1
0.003483	0.00327	53.1
0.003483	0.004437	61.2

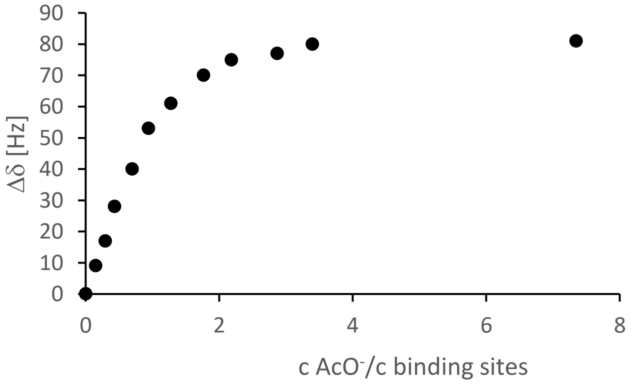

0.003483	0.006144	70.0
0.003483	0.007607	75.1
0.003483	0.009984	77.4
0.003483	0.011833	80.1
0.003483	0.025593	80.9

http://app.supramolecular.org/bindfit/view/3d4a8ae0-6699-470d-8126-6cfe884a56c5

Figure S48. Receptor **10,** ^{1}H NMR titration by $TBA^{+}AcO^{-}$ in DMSO-d_6, Ar*H*.

**c 10, [mol/L]**	**c anion [mol/L]**	**Δδ NH [Hz]**
0.0053	0	0
0.0053	0.0004	16.1
0.0053	0.0008	27.7
0.0053	0.0012	38.8
0.0053	0.0019	59.9
0.0053	0.0026	75.4
0.0053	0.0035	100.0
0.0053	0.0049	128.9
0.0053	0.0061	153.1
0.0053	0.0080	175.4
0.0053	0.0094	197.6
0.0053	0.0213	333.0
0.0053	0.0426	438.0

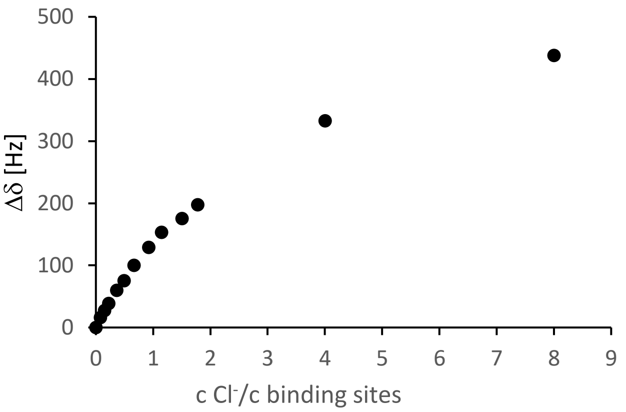

http://app.supramolecular.org/bindfit/view/b455ac26-8c28-4b87-942c-de90af77ba71

Figure S49. Receptor **10,** ^{1}H NMR titration by $TBA^{+}Cl^{-}$ in DMSO-d_6, N*H*.

**c 10, [mol/L]**	**c anion [mol/L]**	**Δδ NH [Hz]**
0.0044	0	0
0.0044	0.0004	0.9
0.0044	0.0009	2.2
0.0044	0.0013	3.5
0.0044	0.0020	5.7
0.0044	0.0031	8.6
0.0044	0.0047	12.3
0.0044	0.0059	15.9
0.0044	0.0081	21.5
0.0044	0.0097	24.7
0.0044	0.021	49
0.0044	0.043	87.7

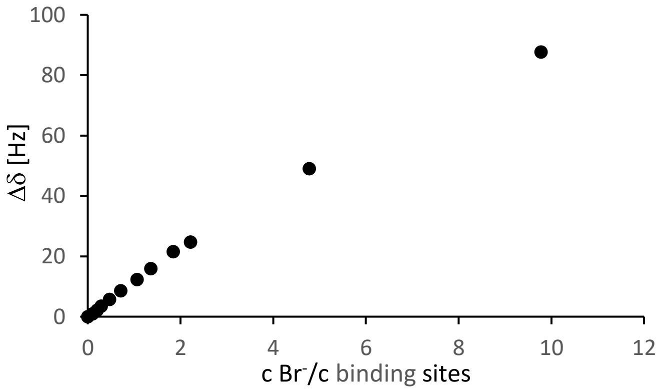

http://app.supramolecular.org/bindfit/view/30129ef1-03e7-4f67-84b5-1ab69134b36a

Figure S50. Receptor **10,** ^{1}H NMR titration by $TBA^{+}Br^{-}$ in DMSO-d_6, N*H*.

c 10, [mol/L]	c anion [mol/L]	Δδ NH [Hz]
0.0044	0	0
0.0044	0.0004	2.3
0.0044	0.0008	4.4
0.0044	0.0012	5.7
0.0044	0.0019	8.1
0.0044	0.0026	10.4
0.0044	0.0036	13.5
0.0044	0.0049	16.8
0.0044	0.0061	19.8
0.0044	0.0080	23.6
0.0044	0.0095	26.2
0.0044	0.02	35
0.0044	0.045	41.1

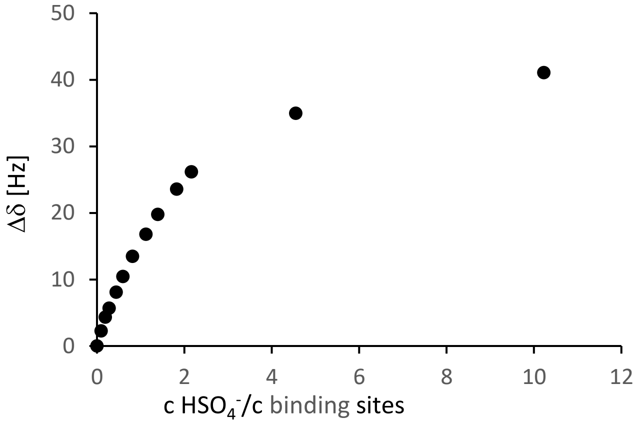

http://app.supramolecular.org/bindfit/view/42f209d2-ea26-4cd7-b833-b6cbf6c34c6a

Figure S51. Receptor **10,** ^{1}H NMR titration by $TBA^+HSO_4^-$ in DMSO-*d*$_6$, Ar*H*.

c 11, [mol/L]	c anion [mol/L]	Δδ NH [Hz]
0.0029	0	0
0.0029	0.0004	-34.4
0.0029	0.0008	36.1
0.0029	0.0012	66.8
0.0029	0.0019	156.3
0.0029	0.0025	245.8
0.0029	0.0034	352
0.0029	0.0047	481
0.0029	0.0058	542.7
0.0029	0.0077	609.8
0.0029	0.0091	630.5

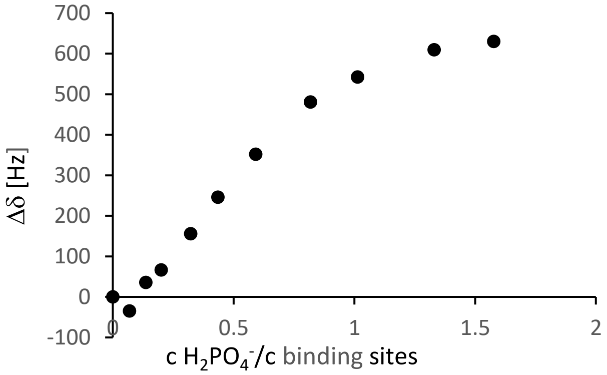

Figure S52. Receptor **11,** ^{1}H NMR titration by $TBA^+H_2PO_4^-$ in DMSO-*d*$_6$, N*H*.

c 12, [mol/L]	c anion [mol/L]	Δδ NH [Hz]
0.0025	0	0
0.0025	0.0004	-92
0.0025	0.0008	-104
0.0025	0.0012	-40
0.0025	0.0019	88
0.0025	0.0025	168
0.0025	0.0034	252
0.0025	0.0047	384
0.0025	0.0058	496
0.0025	0.0077	624

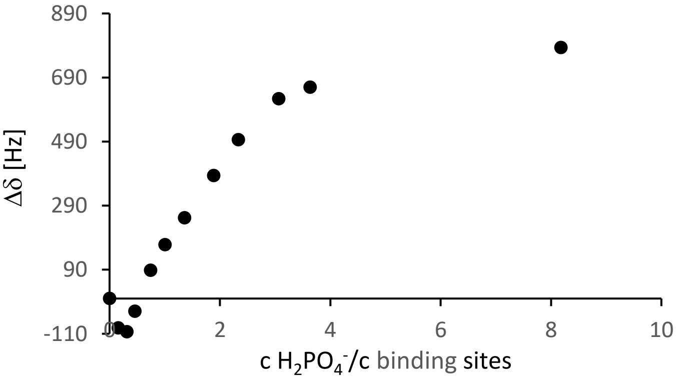

0.0025	0.0091	660
0.0025	0.0204	784

Figure S53. Receptor **12,** ^{1}H NMR titration by $TBA^+H_2PO_4^-$ in DMSO-d_6, N*H*.

c 13, [mol/L]	c anion [mol/L]	Δδ ArH [Hz]
0.0011	0	0
0.0011	0.0004	10.4
0.0011	0.0009	24.9
0.0011	0.0013	38.7
0.0011	0.0021	51
0.0011	0.0028	62.6
0.0011	0.0039	69.3
0.0011	0.0054	72.9
0.0011	0.0067	76.2
0.0011	0.0087	78.9
0.0011	0.0104	80.5
0.0011	0.0234	80.6

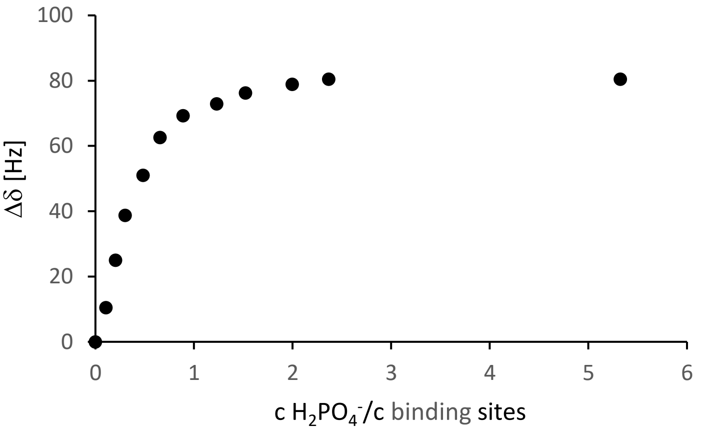

Figure S54. Receptor **13,** ^{1}H NMR titration by $TBA^+H_2PO_4^-$ in DMSO-d_6, Ar*H*.

c 13, [mol/L]	c anion [mol/L]	Δδ NH [Hz]
0.0011	0	0
0.0011	0.0004	14.3
0.0011	0.0008	28.9
0.0011	0.0012	41.0
0.0011	0.0019	63.0
0.0011	0.0026	82.8
0.0011	0.0035	104.1
0.0011	0.0049	135.2
0.0011	0.0061	156.5
0.0011	0.0079	186.2
0.0011	0.0094	208.2
0.0011	0.0212	375.8
0.0011	0.0420	440

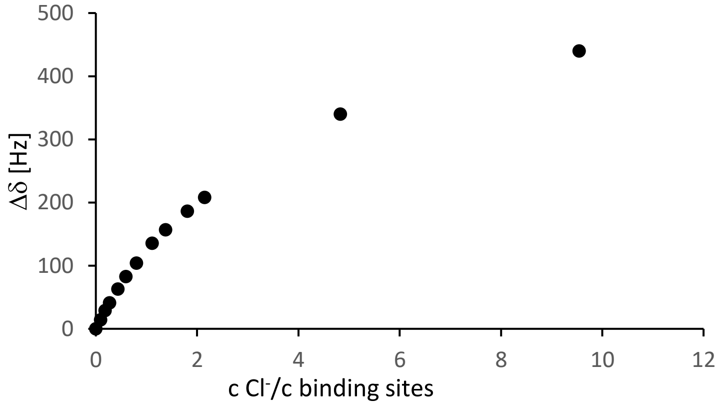

Figure S55. Receptor **13,** ^{1}H NMR titration by TBA^+Cl^- in DMSO-d_6, NH..

c 14, [mol/L]	c anion [mol/L]	Δδ ArH [Hz]
0.0005	0	0
0.0005	0.0004	16.6
0.0005	0.0008	36.4
0.0005	0.0012	50.2
0.0005	0.0019	55.9
0.0005	0.0026	65.8
0.0005	0.0035	70.6
0.0005	0.0049	71.6
0.0005	0.0061	74.5
0.0005	0.0080	76.7
0.0005	0.0095	77.7
0.0005	0.0218	81.9

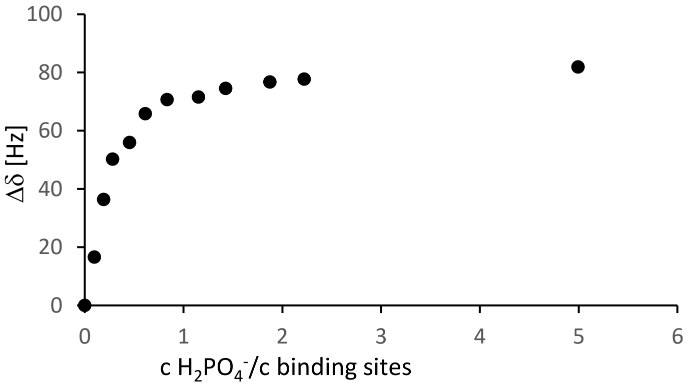

Figure S56. Receptor **14,** ^{1}H NMR titration by $TBA^+H_2PO_4^-$ in DMSO-d_6, Ar*H*.

c 14, [mol/L]	c anion [mol/L]	Δδ NH [Hz]
0.0005	0	0
0.0005	0.0004	17.4
0.0005	0.0009	31.8
0.0005	0.0013	44.9
0.0005	0.0020	62.8
0.0005	0.0028	81
0.0005	0.0037	104.3
0.0005	0.0052	133.9
0.0005	0.0064	154.9
0.0005	0.0084	180.8
0.0005	0.0100	201.7
0.0005	0.0225	337.2
0.0005	0.0420	430

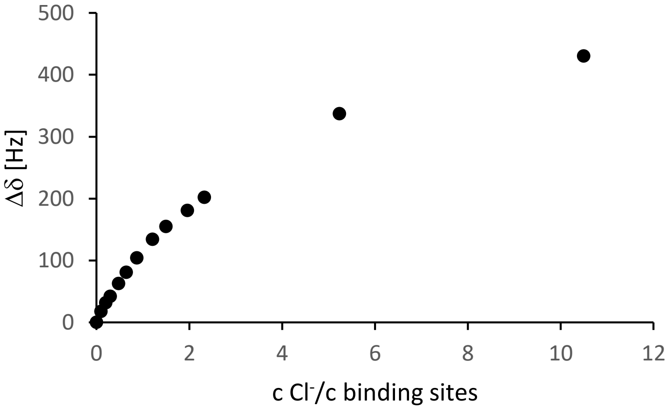

Figure S57. Receptor **14,** ^{1}H NMR titration by TBA^+Cl^- in DMSO-d_6, N*H*.

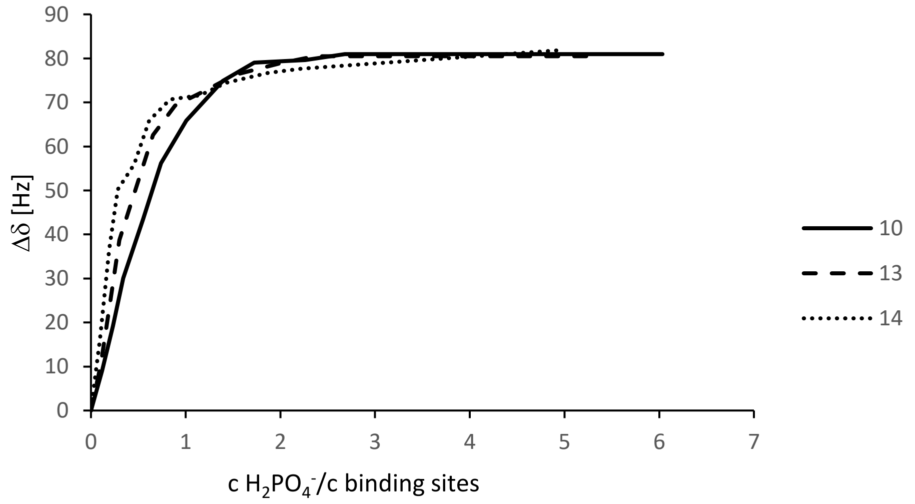

Figure S58. ^{1}H NMR titration by $TBA^{+}H_2PO_4^{-}$ in DMSO-d_6, comparison for receptor **10, 13** and **14**, Ar*H*. An overlay of Figures S46, S54 and S56.

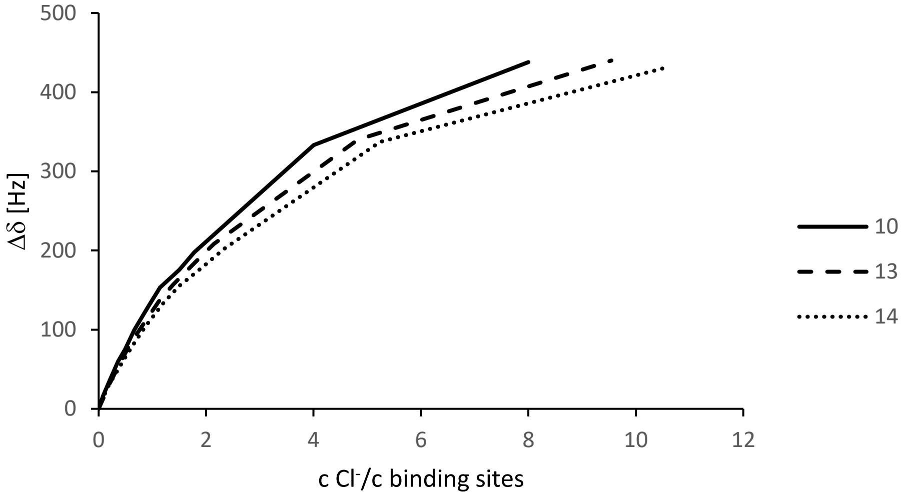

Figure S59. ^{1}H NMR titration by $TBA^{+}Cl^{-}$ in DMSO-d_6, comparison for receptor **10, 13** and **14,** Ar*H*. An overlay of Figures S49, S55 and S57.

**c 10, [mol/L]**	**c anion [mol/L]**	**Δδ ArH - 10 [Hz]**
0.004	0	0
0.004	0.0004	6.8
0.004	0.0009	16.4
0.004	0.0013	27.8
0.004	0.0020	43.2
0.004	0.0028	57.1
0.004	0.0037	70.7
0.004	0.0052	84.0
0.004	0.0064	87.4

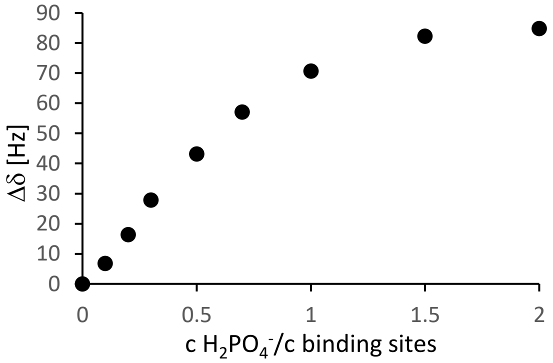

Figure S60. Receptor **10,** ^{1}H NMR titration by $TBA^{+}H_2PO_4^{-}$ in DMSO-d_6, signal of **10** corresponding to signal selected for competitive experiments with **13** and **14,** Ar*H***.**

**c 10, [mol/L]**	**c 13, [mol/L]**	**c anion [mol/L]**	**Δδ ArH - 10 [Hz]**
0.004	0.001	0	0
0.004	0.001	0.0008	4.7
0.004	0.001	0.0018	12.8
0.004	0.001	0.0026	26.2
0.004	0.001	0.0040	48.1
0.004	0.001	0.0056	68.0
0.004	0.001	0.0074	73.9
0.004	0.001	0.0104	83.1
0.004	0.001	0.0128	84.0

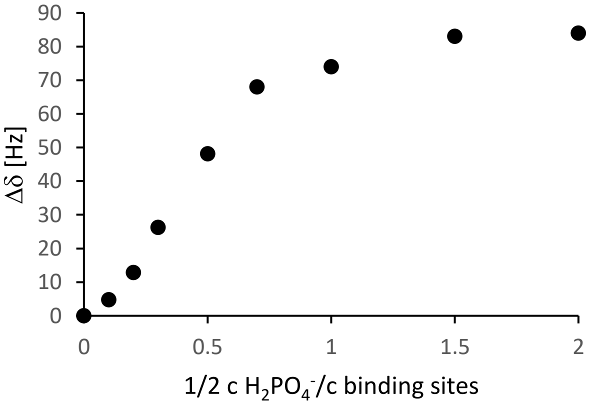

Figure S61. Mixture of receptor **10** and **13,** selected signal of **10,** ^{1}H NMR titration by $TBA^{+}H_2PO_4^{-}$ in DMSO-d_6**,** Ar*H.*

c 10, [mol/L]	c 14, [mol/L]	c anion [mol/L]	Δδ ArH - 10 [Hz]
0.004	0.0005	0	0
0.004	0.0005	0.0008	5.6
0.004	0.0005	0.0018	12.9
0.004	0.0005	0.0026	17.5
0.004	0.0005	0.0040	28.4
0.004	0.0005	0.0056	39.6
0.004	0.0005	0.0074	68.9
0.004	0.0005	0.0104	81.0
0.004	0.0005	0.0128	83.9

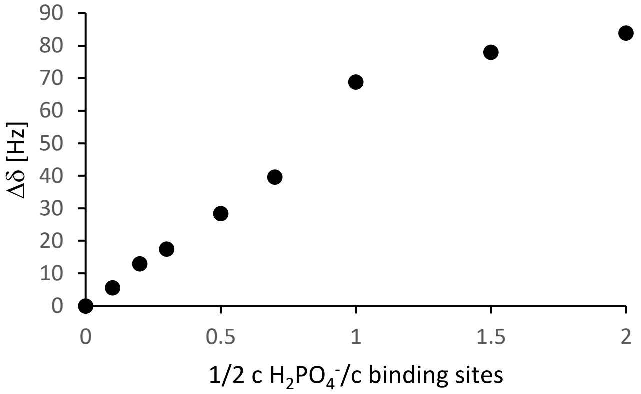

Figure S62. Mixture of receptor **10** and **13,** selected signal of **10,** ^{1}H NMR titration by $TBA^{+}H_2PO_4^{-}$ in DMSO-d_6, Ar*H.*

Equiv $H_2PO_4^-$/group	**10** $\Delta\delta$ [Hz]	**13** $\Delta\delta$ [Hz]
0	0	0
0.1	0	6.92
0.3	0	16.64
0.5	0	23.424
0.7	1.28	29.8
1	1.96	33.12
1.5	1.84	34.16
2	2.28	34.16
2.5	2.76	34.16
3	3.08	34.16
4	3.08	34.16

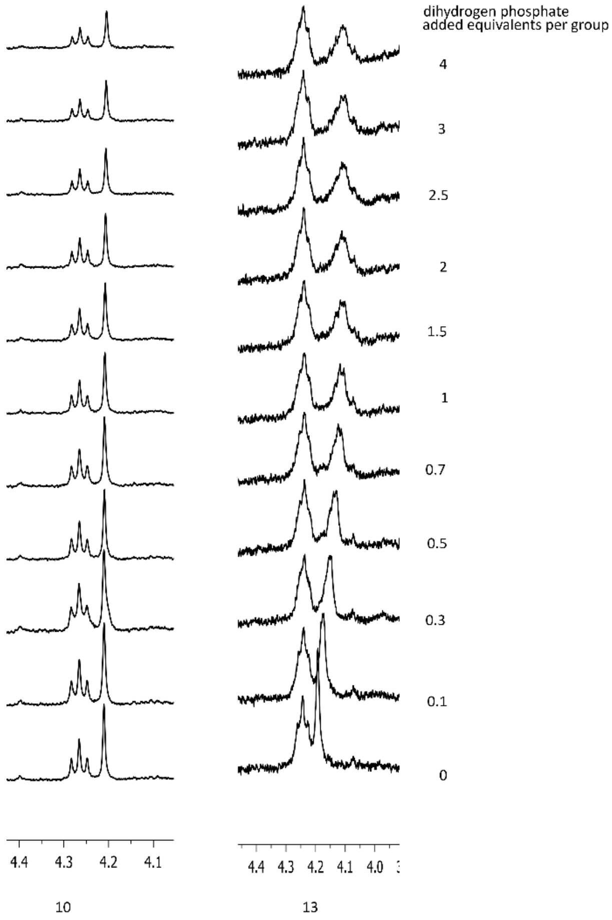

Figure S63. Receptor **10** and **13,** ^{1}H NMR titration by $TBA^+H_2PO_4^-$ in DMSO-*d₆*, complexation induced changes of triazole proton.

X-Ray single crystal diffraction analysis

Crystal data: $C_{17}H_{17}N_3O_3S \cdot C_2H_6OS$, M = 421.54 g.mol^{-1}, triclinic system, space group *P*-1, a = 8.4301 (3) Å, b = 11.0724 (4) Å, c = 12.3965 (4) Å, α = 102.0283 (8) °, β = 97.5228 (8) °, γ = 111.6707 (7) °, Z = 2, V = 1023.84 (6) Å^3, D_c = 1.367 g.cm^{-3}, μ(Cu-Kα) = 2.62 mm^{-1}, crystal dimensions of 0.69 × 0.60 × 0.30 mm, T = 293 (2) K, R = 0.030, wR = 0.0832, using 4023 independent reflections (ϑ_{max} = 72.2°), 262 parameters and 8 restrains, CCDC code 2092890.

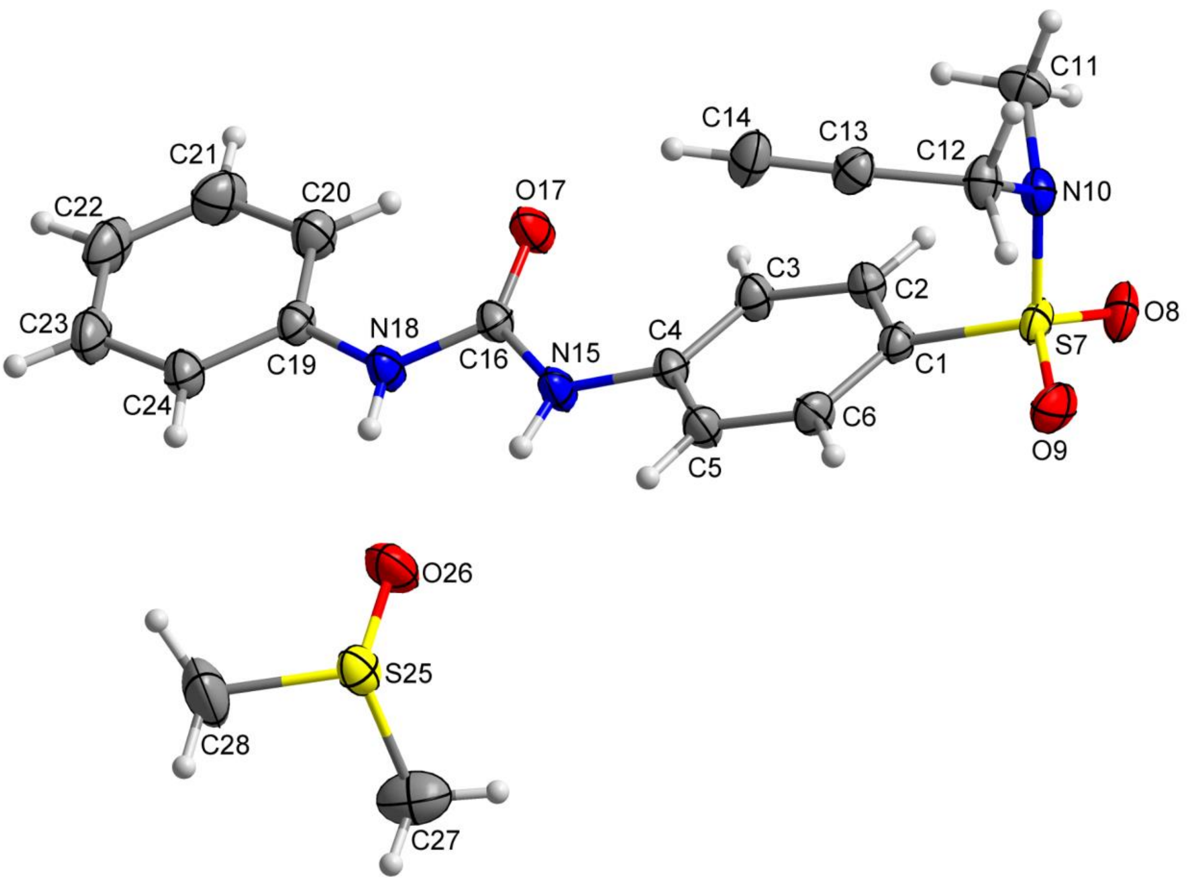

Figure S64. The numbering scheme of structure **4**, with ADPs drawn at 50% probability level
